# Structure–Activity Relationship Study of Splicing Modulators on Hsh155/SF3B1 through Chemical Synthesis and Yeast Genetics

**DOI:** 10.1021/acsmedchemlett.4c00510

**Published:** 2024-11-25

**Authors:** Jacob P. Beard, Sierra L. Love, John C. Schmitz, Aaron A. Hoskins, Kazunori Koide

**Affiliations:** † Department of Chemistry, University of Pittsburgh, 219 Parkman Avenue, Pittsburgh, Pennsylvania 15260, United States; ‡ Department of Biochemistry, University of Wisconsin—Madison, 433 Babcock Drive, Madison, Wisconsin 53706, United States; § Genetics Training Program, University of Wisconsin—Madison, 425 Henry Mall, Madison, Wisconsin 53706, United States; ∥ Division of Hematology-Oncology, Department of Medicine, University of Pittsburgh School of Medicine, 5150 Centre Avenue, Pittsburgh, Pennsylvania 15232, United States; ⊥ Cancer Therapeutics Program, UPMC Hillman Cancer Center, 5117 Centre Ave, Pittsburgh, Pennsylvania 15232, United States; # Department of Chemistry, University of Wisconsin—Madison, 1101 University Avenue, Madison, Wisconsin 53706, United States

**Keywords:** structure−activity relationship, splicing, SF3B1, alternative splicing, anticancer activity

## Abstract

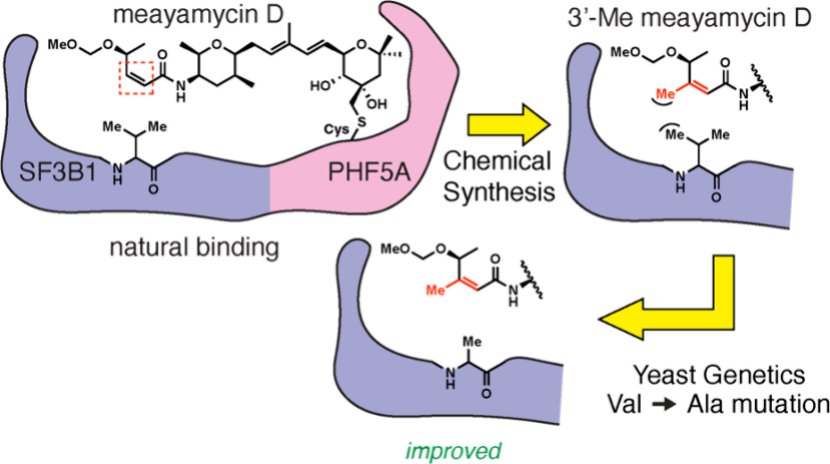

Meayamycins are synthetic analogs of the natural product FR901464 and exhibit potent anticancer activity against human cancers. They bind SF3B1 and PHF5A, components of the human spliceosome, and they alter pre-mRNA splicing. Detailed analysis of the active site led us to investigate a narrow pocket within the binding site that surrounds the α,β-unsaturated amide portion of meayamycin. We describe the synthesis and biological activity of two new analogs bearing a methyl substituent on the α or β position of the amide. With these analogs, we investigated the discrete interactions within the narrow region of SF3B1 using a human/yeast chimeric SF3B1 protein and found that the V1078 residue of SF3B1 affects compound binding at the amide moiety.

FR901464 was isolated from *Burkholderia* sp. FERM BP-3421 as an anticancer agent with half-maximal growth inhibition (GI_50_) values of 1–2 nM against human cancer cells.^1−4^ The molecule binds to human splicing factor 3B subunit 1 (SF3B1), a component of the human spliceosome, to inhibit precursor mRNA splicing.^5^ Pladienolide B,^6−8^ herboxidiene,^9−13^ and other similar natural products^14,15^ were also discovered from natural sources and found to bind to SF3B1 and inhibit splicing.^16,17^ The isolations and biological activities of these natural products have sparked broad interest in the development of therapeutically useful pre-mRNA splicing inhibitors. FR901464 and closely related analogs have been synthesized and biologically evaluated by many groups.^18−27^ The Kitahara group synthetically prepared spliceostatin A (SSA), a more stable 1-methoxy derivative of FR901464 (Figure 1).^20^ After the initial discovery of SF3B1 as the relevant target of FR901464, the Pena group reported the cryo-EM structure of an SSA-bound SF3B complex, a large protein assembly that contains SF3B1 and other SF3B subunits. This structure revealed how the majority of the SSA molecule is bound by SF3B1 near the protein’s interface with another SF3B subunit, plant homeodomain-finger domain 5A (PHF5A).^28^ The structure showed that SSA forms a covalent adduct between the epoxide of SSA and the C26 of PHF5A.

**Figure 1:**
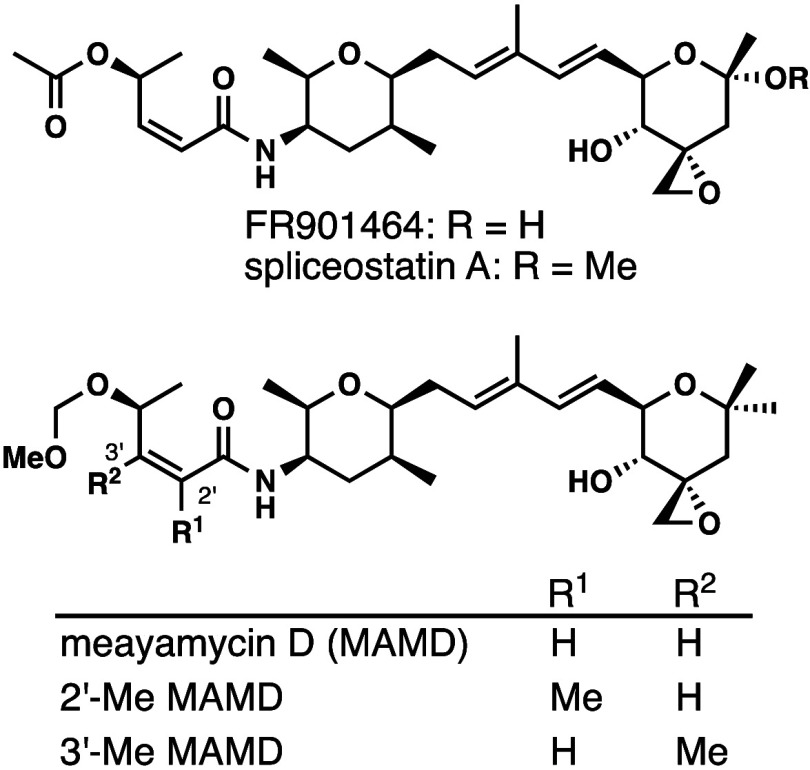
Structures of FR901464 (natural product) and synthetic analogs.

Structure–activity relationship (SAR) studies of FR901464 have primarily focused on the two tetrahydropyran rings, diene moiety, and the C4’ position.^18,25−27,29−37^ In the cryo-EM structure of the SSA-SF3B1 complex, the enamide occupied the narrow neck region of the protein pocket (Figure 2). To gain additional insights, we decided to study the SAR around the C2’ and C3′ positions of FR901464 with our more metabolically stable analog, meayamycin D.^38^ The region between L1066 and V1078 residues of human SF3B1 appear to interact with the C2’ and C3′ positions of SSA. We previously compared the Z-enamide (naturally occurring) to the E-enamide and the C2′-C3′ saturated equivalent.^39^ The latter two compounds were found to be significantly less potent, indicating a possible steric constraint on the C3′ position as well as preference for a rigid C2′-C3′ bond.

**Figure 2:**
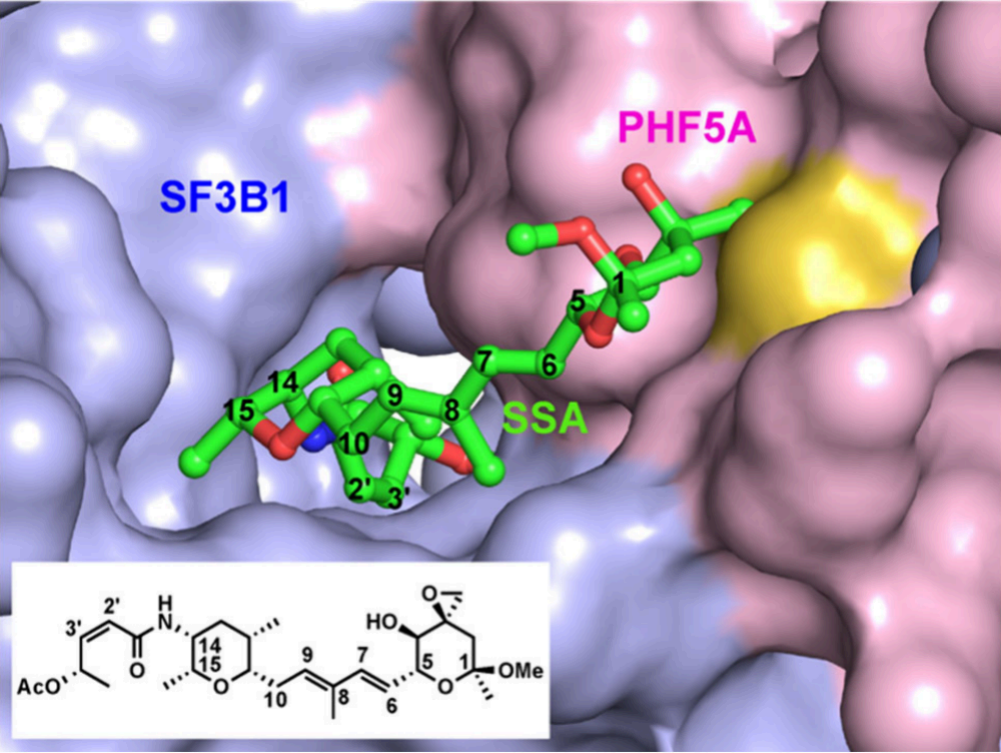
SF3B1 binding pocket occupied by SSA (PDB: 7B9C).

Herein, we report the synthesis and biological evaluation of two new analogs with an additional methyl group at the C2’ or C3′ position. The analogs were evaluated for their antiproliferative activity, splicing activity, and in vitro plasma stability. We reveal that the C3′ substitution is tolerated and leads to a compound with comparable activity to meayamycin D. Lastly, we introduced several mutations into the SF3B1 binding site to understand the discrete interactions at the C2’ and C3′ site that may be required for compound activity.

The synthesis of 2′-Me meayamycin D began with **1**, which was prepared in a single step from commercially available ethyl-(*S*)-lactate (Scheme 1).^38^ Sequentially, lactate **1** was reduced with diisobutylaluminum hydride (DIBALH), and the in situ-generated aldehyde was submitted to an Ando-Horner-Wadsworth-Emmons reaction^40^ with phosphonate **9**(41) to yield the α-methylated enoate **2** in 52% yield, with an E/Z ratio of 6:94. Enoate **2** was hydrolyzed to acid **3** in a quantitative yield. To confirm the correct configuration, we reduced enoate **2** using DIBALH to allylic alcohol **8** in 68% yield. One-dimensional nuclear Overhauser effect (NOE) signals were detected for the C1’ and C4’ positions (Figure S1), confirming the Z-olefin geometry. Acid **3** was coupled with amine **10**(42) to afford amide **4** as an inseparable mixture of isomers. The desired compound could be purified after olefin cross-metathesis with methacrolein, using the nitro-Grela catalyst, to give aldehyde **5** in 39% yield over two steps. Wittig olefination of aldehyde **5** with Ph_3_P=CH_2_ gave diene **6** in 78% yield. Finally, this diene was united with fragment **7** to afford 2′-Me meayamycin D in 8% yield.

**Scheme 1:**
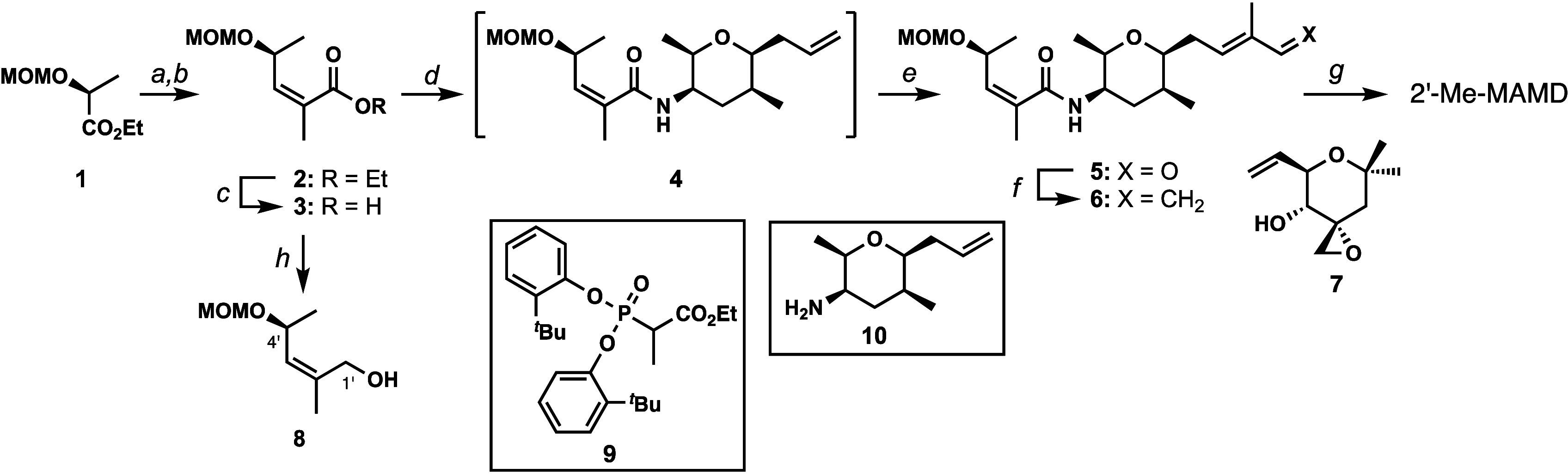
Synthesis of 2′-Me Meayamycin D Conditions: (a) diisobutylaluminum hydride (DIBALH), CH_2_Cl_2_, −78 °C, 2 h; then (b) **9**, KO^*t*^Bu, tetrahydrofuran (THF), −78 °C to rt, 20 h, 52% (E:Z = 6:94); (c) NaOH, MeOH, 0 °C to rt, 16 h, quant.; (d) **10**, HATU, diisopropylethylamine, CH_2_Cl_2_, 0 °C to rt, 42 h, inseparable mixture; (e) methacrolein, nitro-Grela catalyst, 50 °C, 20 h, 39% for 2 steps; (f) Ph_3_PCH_3_Br, KO^*t*^Bu, THF, 0 °C to rt, 18 h, 78%; (g) **7**, nitro-Grela catalyst, dichloroethane (DCE), 50 °C, 8 h, 8%; (h) DIBALH, THF, −78 °C, 1.5 h, 68%.

The synthesis of 3′-Me meayamycin D started with the hydrolysis of **1** to acid **11** in 84% yield (Scheme 2). Acid **11** was treated with trimethylacetyl chloride followed by *N,O*-dimethylhydroxylamine to give Weinreb amide **12** in 83% yield. Attempts to directly convert ester **1** to amide **12** failed. Grignard addition of in situ-generated MeMgI to amide **12** gave ketone **13** in 88% yield, which was directly subjected to an Ando-Horner-Wadsworth-Emmons olefination with phosphonate **20** in the presence of KO^*t*^Bu to afford the β-methylated enoate **14** in 50% yield, with an E/Z ratio of 15:85. The olefin geometry of enoate **14** was confirmed using the same method as that for enoate **2**. Reduction of enoate **14** using DIBALH gave allylic alcohol **19** in 79% yield. Correlating NOE signals were observed between the C1’ and C4’ positions, indicating a Z-olefin geometry (Figure S2). Next, enoate **14** was hydrolyzed to acid **15** quantitatively, which was coupled with amine fragment **10** to afford amide **16** as an inseparable mixture of isomers. In a similar fashion, the desired compound was separated after olefin cross-metathesis with methacrolein, using nitro-Grela catalyst, to afford aldehyde **17** in 32% yield over two steps. Wittig olefination of aldehyde **17** with Ph_3_P = CH_2_ gave diene **18** in an 82% yield. Cross olefin metathesis of diene **18** with the right fragment **7** gave 3′-Me meayamycin D in 8% yield.

**Scheme 2:**
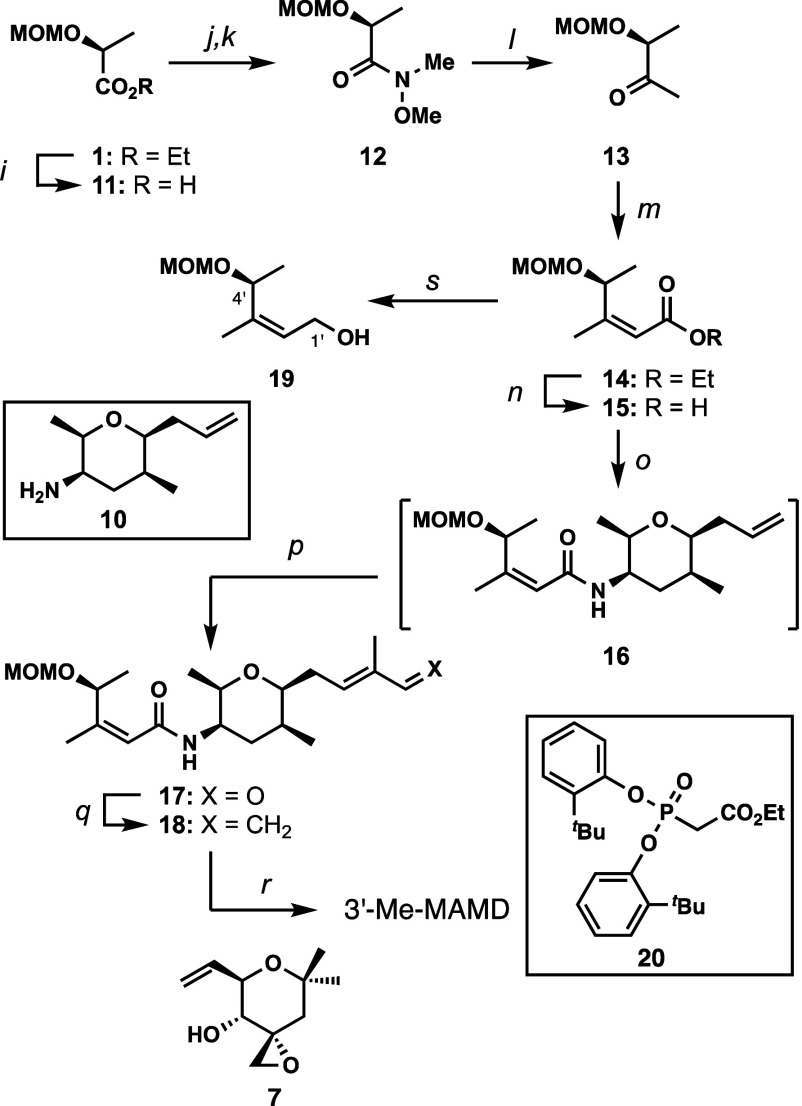
Synthesis of 3′-Me Meayamycin D Conditions: (i) LiOH, MeOH, H_2_O, 3 h, 0 °C, 84%; (j) trimethylacetyl chloride, triethylamine, CH_2_Cl_2_, 0 °C, 1.5 h; then (k) *N*,*O*-dimethylhydroxylamine hydrochloride, triethylamine, CH_2_Cl_2_, 0 °C to rt, 20 h, 83%; (l) Mg, MeI, Et_2_O, 0 °C, 2.5 h, 88%; (m) **20**, KO^*t*^Bu, THF, 0 °C to rt, 18 h, 50% (E:Z = 15:85); (n) NaOH, MeOH, 0 °C, 3.5 h, quant.; (o) **10**, HATU, diisopropylamine, CH_2_Cl_2_, 0 °C to rt, 40 h, inseparable mixture; (p) methacrolein, nitro-Grela catalyst, 50 °C, 18 h, 32% for 2 steps; (q) Ph_3_PCH_3_Br, KO^*t*^Bu, THF, 0 °C, 1.5 h, 82%; (r) **7**, nitro-Grela catalyst, DCE, 45 °C, 14 h, 8%; (s) DIBALH, THF, −78 °C, 2 h, 79%.

With 2′-Me meayamycin D and 3′-Me meayamycin D in hand, we evaluated the antiproliferative activity of the compounds in several human cancer cell lines (Table 1 and Figure S3). 2′-Me meayamycin D was significantly less potent with a GI_50_ of 127–240 nM. Compared to the nonsubstituted analog, meayamycin D, this is approximately 2 orders of magnitude less potent.^38^ During this work, the Arisawa group reported the synthesis and activity of a similar 2′-methylpentenamide derivative.^43^ In their study, they reported that the 2′-methylpentenamide derivative gave less inhibitory activity against androgen receptor splice variant 7 (AR-V7) expression, as compared to SSA. As mentioned above, the crystallographic data suggest that this position lies in a relatively narrow space within the protein binding site. Given this, it is possible that the lower antiproliferative activity is due to steric clash between the C2′-methyl and the protein binding pocket of SF3B1. 3′-Me meayamycin D, is significantly more potent with GI_50_ of 4.6–7.2 nM. This suggests that methylation at the C3′ position is tolerated while methylation at the C2’ position results in a significant loss in activity.

**Table 1:** Antiproliferative Activity of Meayamycin A, Meayamycin D, 2′-Me Meayamycin D, and 3′-Me Meayamycin D in Various Human Cancer Cell Lines^a^

	GI_50_ (nM)	
Cell lines	2′-Me meayamycin D	3′-Me meayamycin D
HCT116	129 ± 14	4.8 ± 0.9
SW48	127 ± 15	4.6 ± 0.7
A549	240 ± 48	7.2 ± 2.1
DMS53	169 ± 23	5.9 ± 1.0
DMS114	153 ± 20	5.5 ± 1.3

a Each value represents the average of *n* ≥ 3 replicates, SD.

We evaluated the ability of 2′-Me meayamycin D and 3′-Me meayamycin D to decrease the abundance of proteins whose expression is dependent on splicing of their respective pre-mRNAs (Figure 3). 3′-Me meayamycin D (GI_50_ = 5 nM) showed a comparable decrease in myeloid cell leukemia 1 (MCL-1) protein abundance to meayamycin D (GI_50_ = 2 nM),^38^ while 2′-Me meayamycin D (GI_50_ = 129 nM) showed only small changes in protein levels at concentrations up to 1 μM. Treatment with these analogs also led to a dose-dependent decrease of *MCL-1* alternative splicing mirroring the expression levels of MCL-1, which has been previously observed in other meayamycin analogs (Figure S4).^44,45^ These results corroborate the antiproliferative assay results and may serve as one explanation for the lower activity of 2′-Me meayamycin D. Interestingly, we observed a nearly negligible increase in a proteoform of p27 generated by alternative splicing of the coding pre-mRNA, as compared to meayamycin D. All compounds also lead to a decrease in SF3B1 phosphorylation, consistent with disruption of the splicing process.^46^ Next, we investigated the stability of 2′-Me meayamycin D and 3′-Me meayamycin D in mouse CD1 plasma (Figure S5). 3′-Me meayamycin D has comparable stability in plasma compared to meayamycin D (*t*_1/2_ = 13 h)^38^ with a half-life of 16 h. Meanwhile, 2′-Me meayamycin D has a higher half-life of 30 h, which may be attributed to steric shielding of the amide bond.

**Figure 3:**
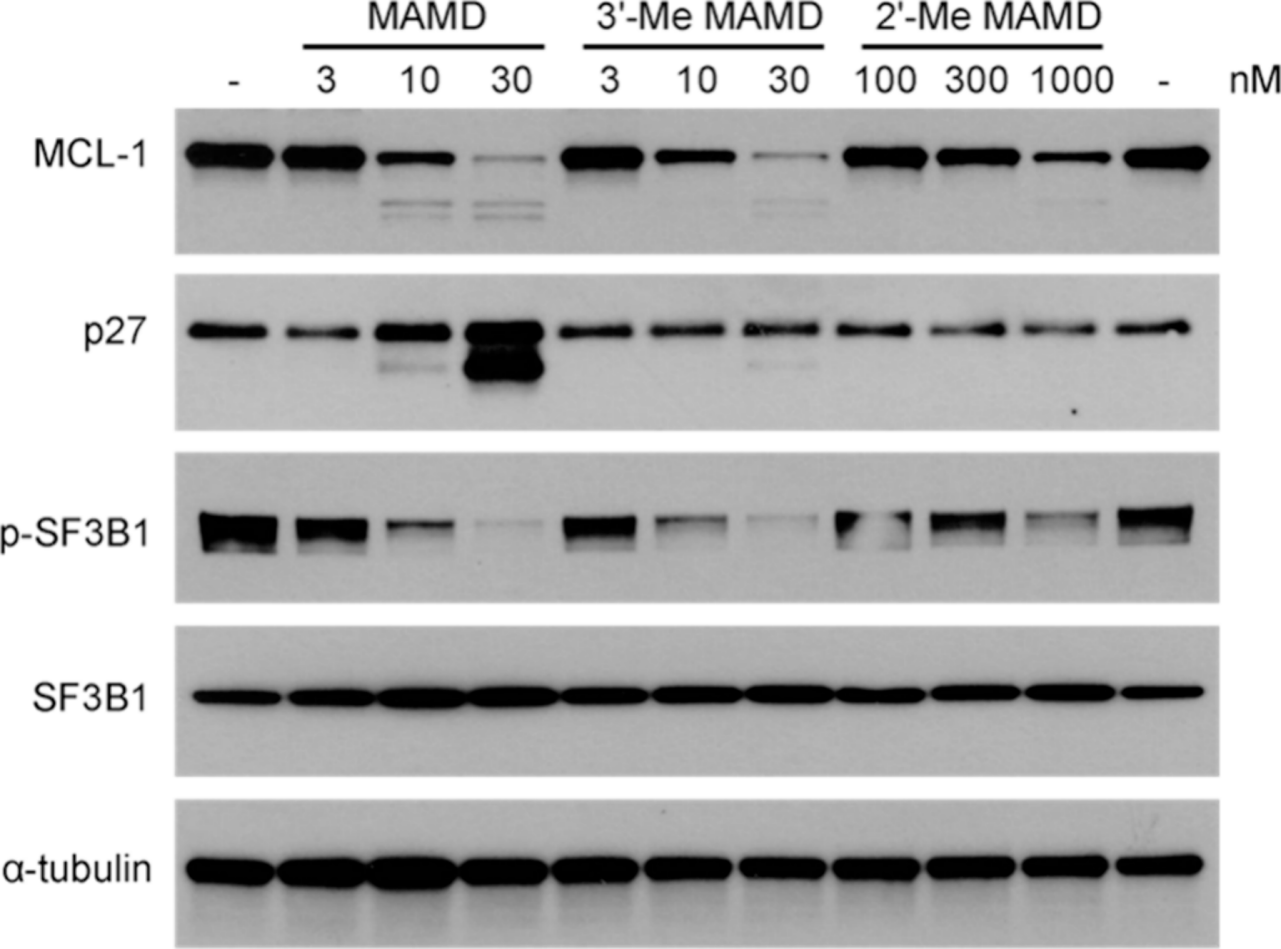
Western blot analysis of HCT116 cells treated with meayamycin D (MAMD), 3′-Me MAMD, and 2′-Me MAMD.

To better understand the binding pocket for meayamycin D on SF3B1, we analyzed the crystal structure^28^ and identified four residues that are near the C2’ and C3′ methyl group: L1066, R1074, T1077, and V1078 (Figure 4). We wondered whether the replacement of these residues with less bulky amino acids would improve the potency of the C2′-methylated analog. Using previously established methods,^47,48^ we generated a human/yeast chimeric SF3B1 protein in *S. cerevisiae*. The chimeric protein has HEAT domains 5–16 of the wild-type yeast SF3B1 (Hsh155) replaced with the human SF3B1 sequence (denoted as Hs5–16). We have previously shown these domains comprise the binding site for SSA and other small molecule splicing inhibitors and are responsible for the observed splicing effect of such compounds.^48^ This chimera model provides a genetically tractable and facile way to detect splicing inhibition, since pre-mRNA splicing is essential in yeast. With this model in hand, we replaced the residues (L1066, R1074, T1077, V1078) with either alanine or glycine and compared the growth inhibition between meayamycin D, 2′-Me meayamycin D, 3′-Me meayamycin D, and herboxidiene (control) in *S. cerevisiae* with Hs5–16 (Figures S6 and S7). Meayamycin D has an approximate GI_50_ of 108 nM in unmodified Hs5–16, while 2′-Me meayamycin D does not inhibit growth at concentrations up to 1 μM (Figure S6, black curve). 3′-Me meayamycin D has a GI_50_ of 405 nM in Hs5–16, which is a trend similar to the observed activity of these compounds in human cancer cells. Both the L1066A and T1077A mutants were still inhibited by meayamycin D, albeit with less potency. Additionally, none of the selected mutations improved the compound activity of 2′-Me meayamycin D. One possibility is that the steric clash of the 2′-methyl may occur primarily between the protein backbone rather than the specific amino acids. In the case of L1066G, this residue lies close to the beginning of the α-helical fold. Therefore, replacement with the more flexible glycine may destabilize the α-helix and lead to decreased compound activity (although yeast can tolerate this substitution in an essential protein). This flexibility may also explain the observed weaker toxicity in the T1077G mutant as well for meayamycin D and 3′-Me meayamycin D. Notably, however, herboxidiene did not lose toxicity against the T1077G mutation (Figure S7). Finally, the V1078A mutant showed enhanced effect with 3′-Me meayamycin D with a GI_50_ of 80 nM (Hs5–16 GI_50_ = 405 nM; Figure 5), indicating that the valine at this position may be closer in proximity to the added 3′-methyl group.

**Figure 4:**
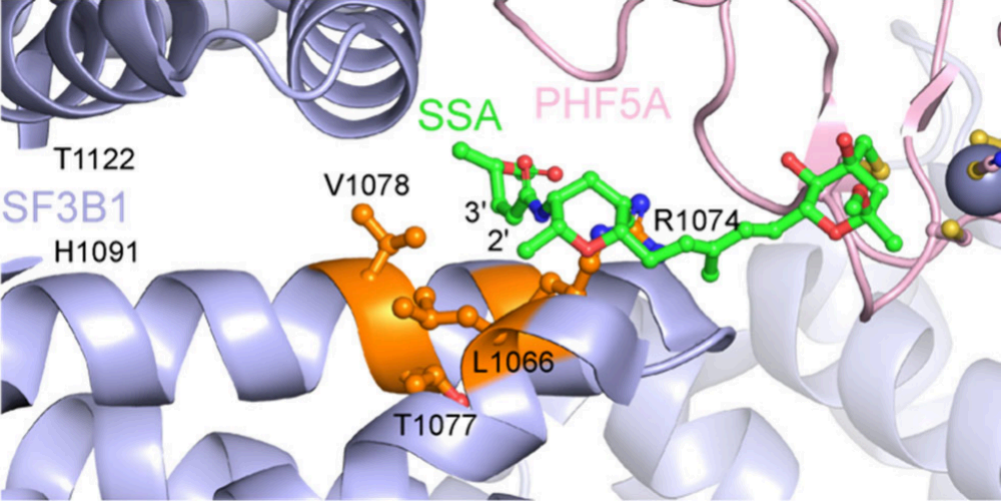
Proximal residues to the C2’ and C3′ position in the SSA-SF3B1 crystal structure (PDB: 7B9C, residues H1091–T1122 omitted for clarity).

**Figure 5:**
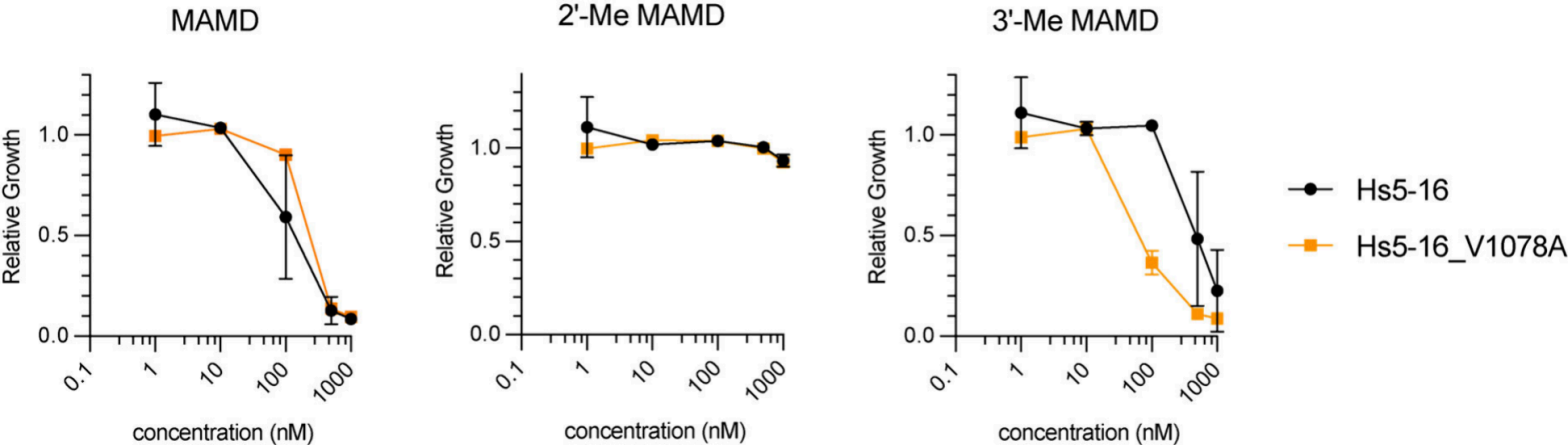
Growth inhibition of *S. cerevisiae* with chimeric Hs5–16 mutation V1078A in the presence of meayamycin D (MAMD), 2′-Me MAMD, and 3′-Me MAMD. Each point represents the average of *n* = 3 biological replicates, ± SD.

In conclusion, we designed and synthesized two new analogs bearing methyl groups on the C2’ and C3′ positions. Our biological evaluation revealed that the C2’ position is not tolerated for substitution. In contrast, the C3′ substitution still retains modest activity compared to meayamycin D. 3′-Me meayamycin D inhibited the alternative splicing of *MCL-1* similar to meayamycin D, indicating these compounds likely behave similarly to affect cancer cell growth. Additionally, we investigated interactions within the SF3B1 binding pocket using a chimeric SF3B1 protein in yeast to understand the binding of these substituted analogs. None of the mutants tested improved the ability of 2′-Me meayamycin D to inhibit growth, consistent with the intolerability for substitution at this position. Meanwhile, a V1078A mutant was identified to have enhanced activity with 3′-Me meayamycin D in comparison to both meayamycin D and the unmutated chimeric protein. The 3′-Me meayamycin D analog highlights a new position on FR901464-based compounds that is suitable for single carbon or single atom substitutions without a significant loss in potency and justifies further exploration of the binding pocket through structure–activity relationship studies.

## Supporting Information Available

Selective 1D NOE spectrum of compounds **8** and **19**; ^1^H and ^13^C{^1^H} NMR spectrum of synthesized compounds; Antiproliferative assay data; RT-PCR; in vitro plasma stability; *S. cerevisiae* growth inhibition assays; detailed synthetic procedures; and general experimental protocols (PDF)

## Notes

Safety: No unexpected or unusually high safety hazards were encountered.

## Abbreviations

GI50: Half-maximal growth inhibition
MCL-1L: myeloid cell leukemia-1 long isoform
MCL-1S: myeloid cell leukemia-1 short isoform
SD: standard deviation
PHF5A: PHD finger protein 5A
SF3B1: splicing factor 3B subunit 1
