# Supplementary material for: Structure–Activity Relationship Study of Splicing Modulators on Hsh155/SF3B1 through Chemical Synthesis and Yeast Genetics
Source: ACS Med Chem Lett. 2024 Nov 25;15(12):2225–30. doi: 10.1021/acsmedchemlett.4c00510 (PMC11647714; doi:10.1021/acsmedchemlett.4c00510)
Supplement: Supplementary file 1 — ml4c00510_si_001.pdf [file ml4c00510_si_001.pdf]

Supporting Information

for

Structure-activity relationship study of splicing modulators on Hsh155/SF3B1 through chemical synthesis and yeast genetics

Jacob P. Beard[1], Sierra L. Love[2,3], John C. Schmitz[4,5], Aaron A. Hoskins[2,6], and Kazunori Koide[1,5]*

[1]Department of Chemistry, University of Pittsburgh
219 Parkman Avenue, Pittsburgh, Pennsylvania 15260, United States

[2]Department of Biochemistry, University of Wisconsin-Madison
433 Babcock Drive, Madison, Wisconsin 53706, United States

[3]Genetics Training Program, University of Wisconsin-Madison
425 Henry Mall, Madison, Wisconsin 53706, United States

[4]Division of Hematology-Oncology, Department of Medicine, University of Pittsburgh School of Medicine
5150 Centre Avenue, Pittsburgh, Pennsylvania 15232, United States

[5]Cancer Therapeutics Program, UPMC Hillman Cancer Center
5117 Centre Ave, Pittsburgh, Pennsylvania 15232, United States

[6]Department of Chemistry, University of Wisconsin-Madison
1101 University Avenue, Madison, Wisconsin 53706, United States

koide@pitt.edu

Table of Contents

Figure S1. 1D selective NOESY spectra of allylic alcohol **8** S3

Figure S2. 1D selective NOESY spectra of allylic alcohol **19** S4

Figure S3. Antiproliferative assays S5

Figure S4. RT-PCR S5

Figure S5. In vitro plasma stability S6

Figure S6–7. Growth inhibition assays of *S. cerevisiae* S6-7

Figure S8. GCMS fragmentation of ketone **13** S7

Synthetic Procedures S8

Growth Inhibition Assay S17

Immunoblot Analysis S17

RT-PCR S17

In Vitro Plasma Stability S18

Preparation of Yeast Strains S18

Microplate Yeast Growth Assay S18

References S19

NMR Spectra S20

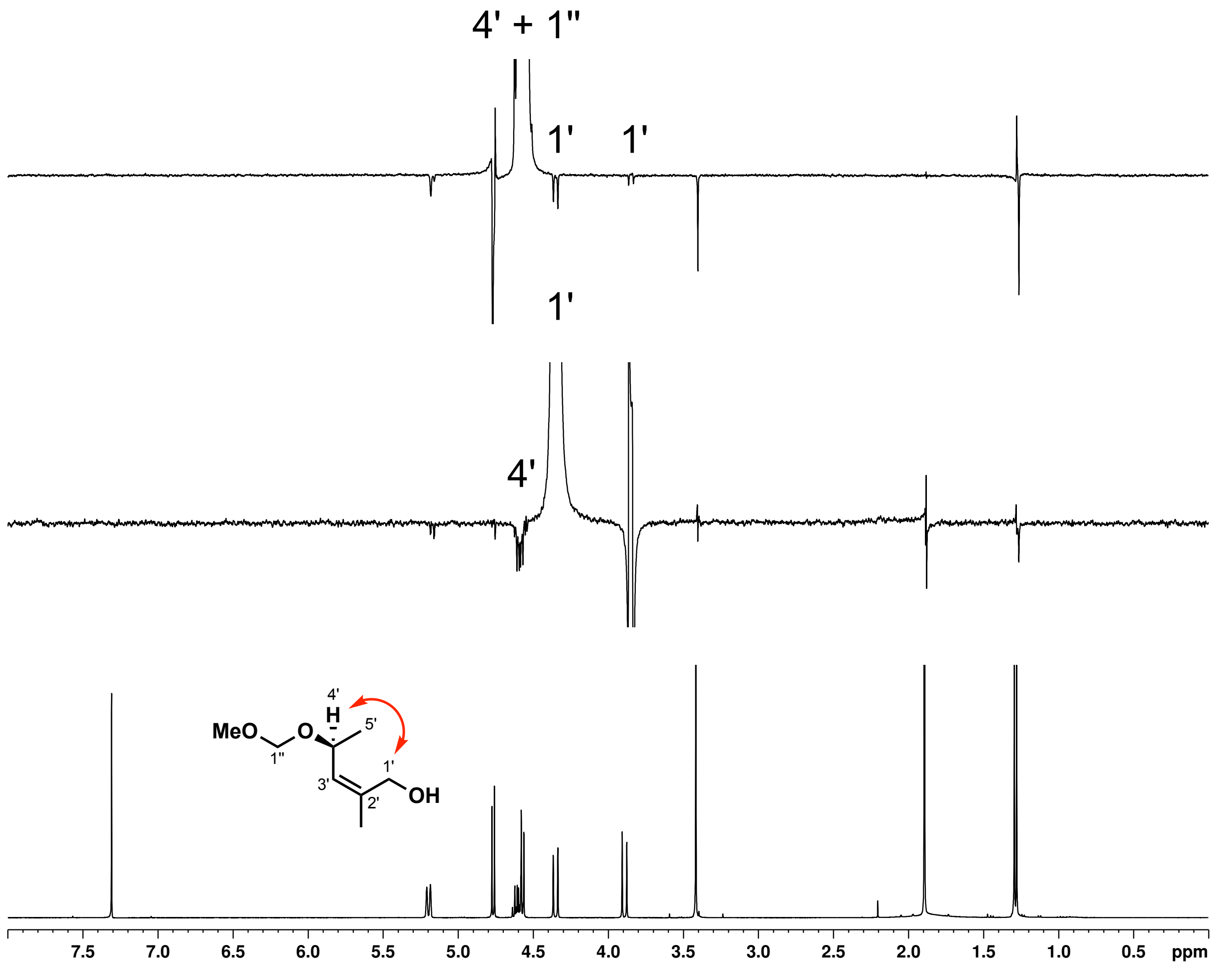

Figure S1. Selective 1D NOESY spectra of (*S*,*Z*)-4-(methoxymethoxy)-2-methylpent-2-en-1-ol **8** (400 MHz, 1% CD_3OD in $CDCl_3$, 296K)

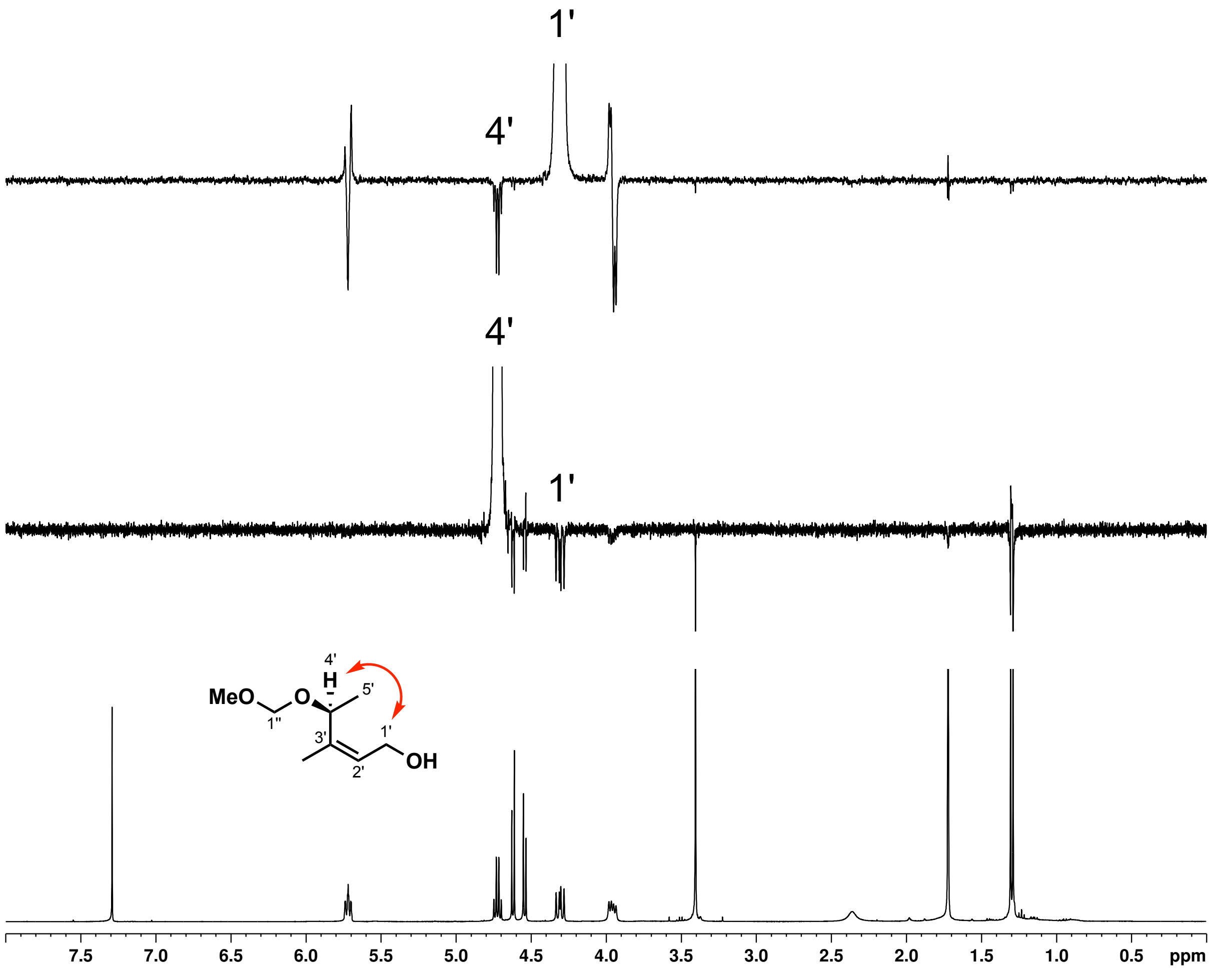

Figure S2. Selective 1D NOESY spectra of (*S*,*Z*)-4-(methoxymethoxy)-3-methylpent-2-en-1-ol **19** (400 MHz, 1% CD_3OD in $CDCl_3$, 296K)

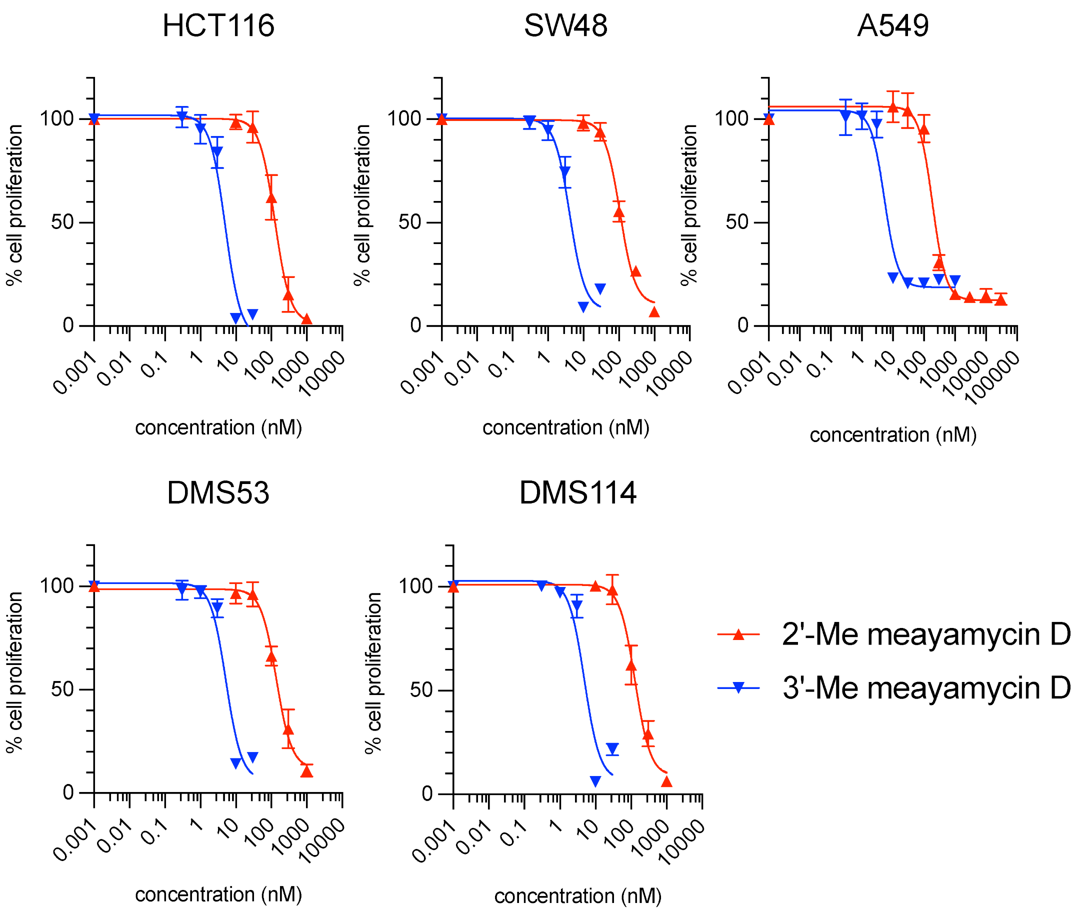

Figure S3. Antiproliferative assays. Each point represents the average of n = 3 replicates, ± SD.

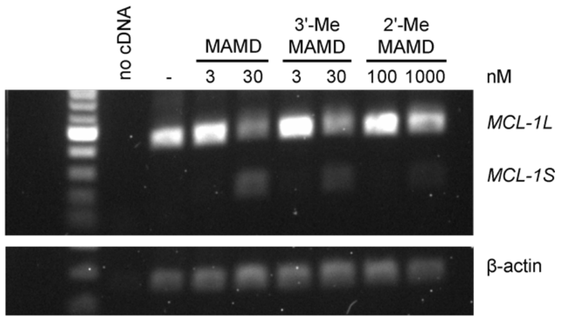

Figure S4. RT-PCR analysis of HCT116 cells treated with meayamycin D (MAMD), 3'-Me MAMD, and 2'-Me MAMD.

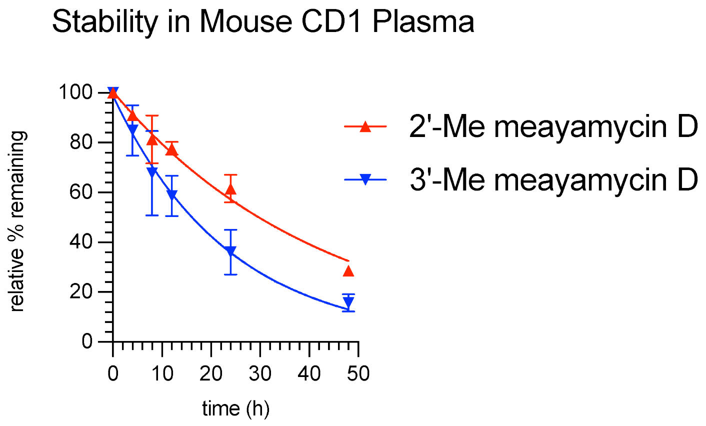

Figure S5. In vitro plasma stability of 2'-Me meayamycin D and 3'-Me meayamycin D in mouse CD1 plasma. Each point represents the average of $n = 2$ replicates, ± SD.

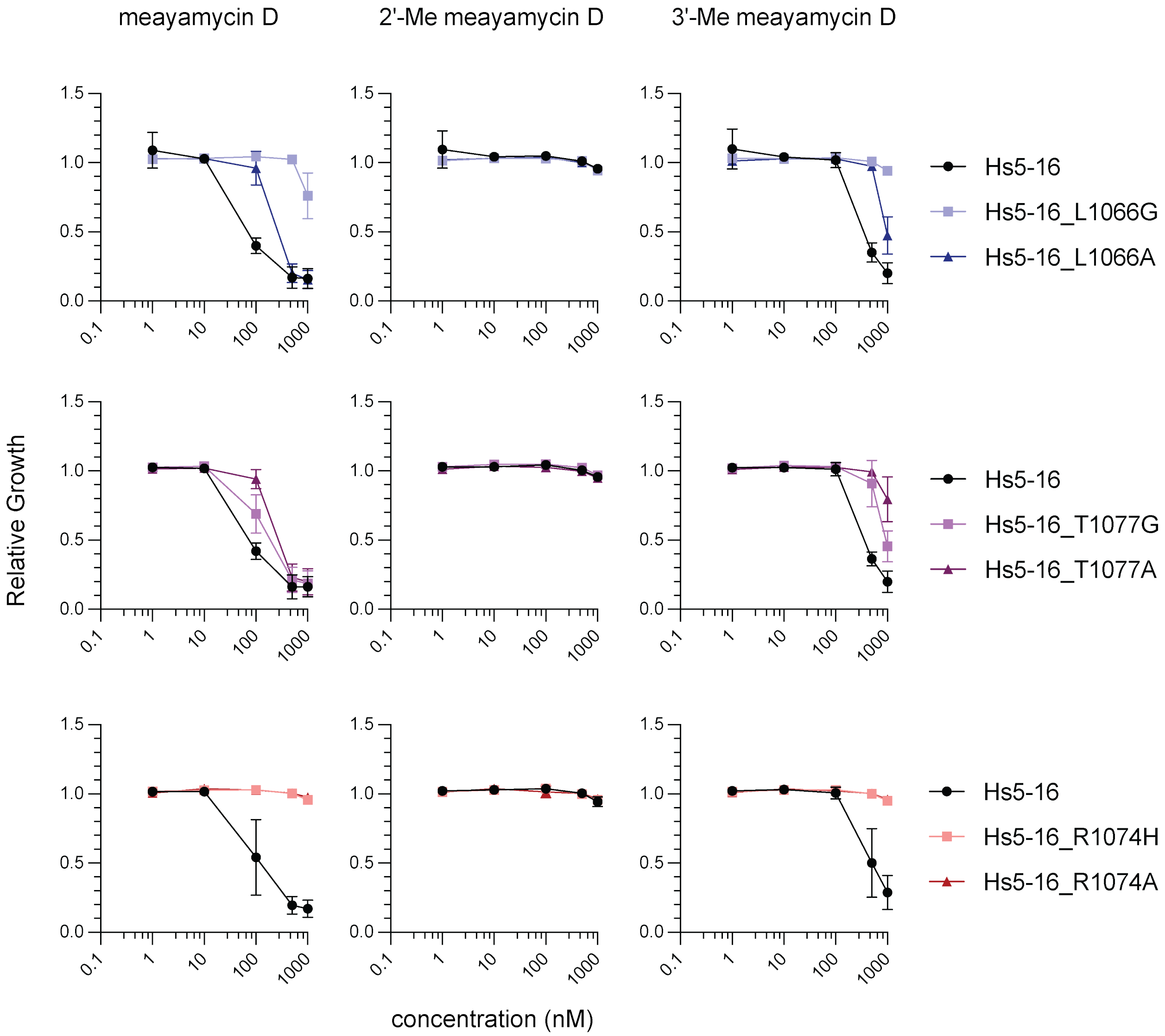

Figure S6. Growth inhibition assays of *S. cerevisiae* harboring Hs5-16 mutations. Each point represents the average of $n \geq 3$ replicates, ± SD.

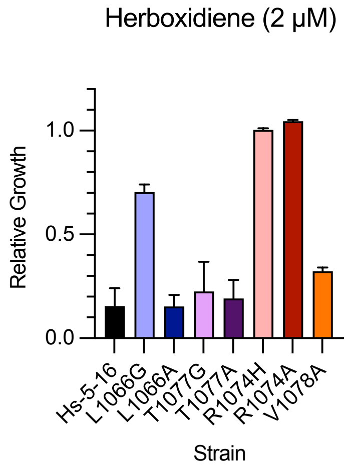

Figure S7. Growth inhibition of *S. cerevisiae* mutants treated with herboxidiene (2 μM). Each bar represents the average of $n \geq 3$ replicates, ± SD.

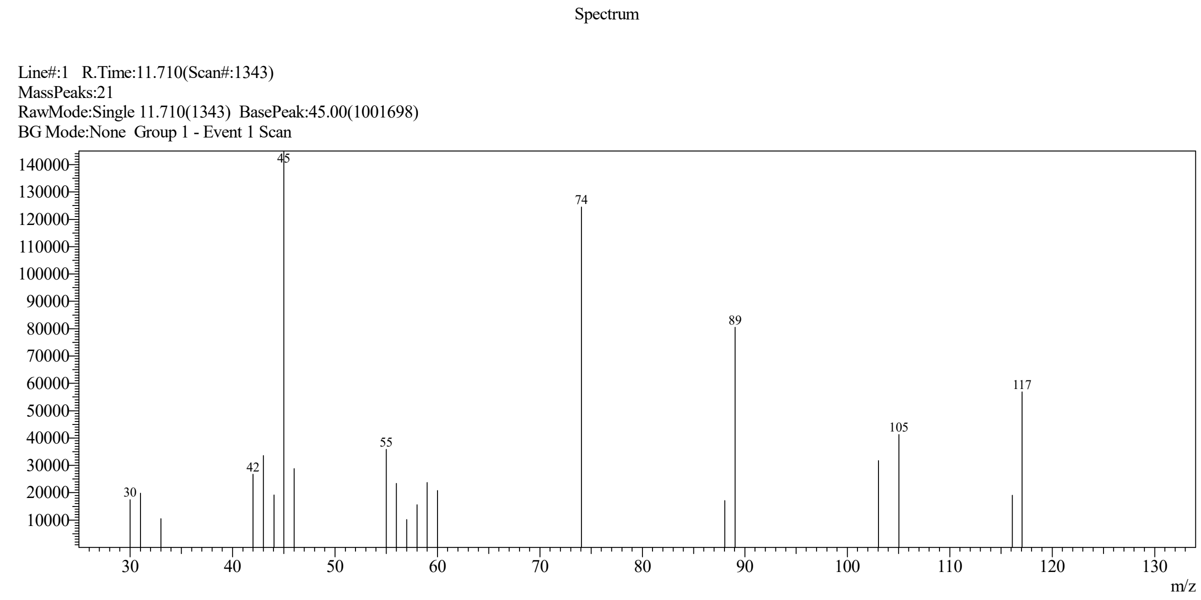

Figure S8. GCMS fragmentation of (*S*)-3-(methoxymethoxy)butan-2-one **13**

Synthetic Procedures

Safety. No unexpected or unusually high safety hazards were encountered.

Chemistry. All reactions were carried out with freshly distilled solvents under anhydrous conditions unless otherwise noted. All flasks used for carrying out reactions were dried in an oven at 80 °C prior to use. Unless otherwise stated, all reactions that required heating used an oil bath as the heating source, with a thermometer submerged in the bath to monitor the temperature. Unless specifically stated, the temperature of a water bath during the evaporation of organic solvents using a rotary evaporator was about 35 ± 5 °C. THF was distilled over Na metal and benzophenone. CH_2Cl_2 was distilled over calcium hydride. MeCN was distilled over calcium hydride and stored over 3 Å molecular sieves. Yields refer to chromatographically and spectroscopically (^{1}H NMR) homogeneous materials unless otherwise stated. All reactions were monitored by thin-layer chromatography (TLC) carried out on 0.25-mm Merck silica gel plates (60F-254) using UV light (254 nm) for visualization or anisaldehyde in ethanol or 0.2% ninhydrin in ethanol as developing agents and heat for visualization. Silica gel (230−400 mesh) was used for flash column chromatography. NMR spectra were recorded on a Bruker ADVANCE spectrometer at 300, 400, 500, or 600 MHz. The chemical shifts are given in parts per million (ppm) on a delta (δ) scale. The solvent peak was used as a reference value for ^{1}H NMR: C*H*Cl$_3$ = 7.26 ppm, C*H*$_2$Cl$_2$ = 5.32 ppm, for ^{13}C{^{1}H} NMR: $CDCl_3$ = 77.16 ppm, CD_2Cl_2 = 53.84 ppm. The following abbreviations are used to indicate the multiplicities: s = singlet; d = doublet; t = triplet; q = quartet; m = multiplet; br = broad. High-resolution mass spectra were recorded on a Thermo Scientific Q Exactive Orbitrap. Infrared (IR) spectra were collected on a PerkinElmer FT-IR Spectrum Two UATR spectrometer. Optical rotation was obtained on a Jasco P-2000 Digital Polarimeter, and $[\alpha]_D^T$ values are given in deg×cm^3×g^{-1}×dm^{-1}; concentrations, *c*, are listed in g×100×mL^{-1}. The purity of all final compounds was determined to be >95% by HPLC.

*Ethyl (S,Z)-4-(methoxymethoxy)-2-methylpent-2-enoate (**2**)*. A 250-mL round-bottom flask equipped with an addition funnel and ethyl (*S*)-2-(methoxymethoxy)propanoate **1** (2.50 g, 15.4 mmol) was purged with nitrogen gas three times and then charged with CH_2Cl_2 (38 mL). The mixture was cooled to -78 °C, and DIBALH (1.0 M in hexanes, 23 mL, 1.5 equiv) was added dropwise down the side of the flask over 30 min at -78 °C with the addition funnel. After stirring for 1.5 h at -78 °C, a separate 100-mL round-bottom flask with ethyl 2-(bis(2-(*tert*-butyl)phenoxy)phosphoryl)propanoate **9** (8.95 g, 20.0 mmol, 1.3 equiv) was purged with nitrogen gas three times and then charged with THF (25 mL) and cooled to 0 °C. Potassium *tert*-butoxide (1.94 g, 17.0 mmol, 1.1 equiv) was added in one portion to the flask with ethyl 2-(bis(2-(*tert*-butyl)phenoxy)phosphoryl)propanoate **9** at 0 °C. After 45 min at 0 °C, the solution of ethyl 2-(bis(2-(*tert*-butyl)phenoxy)phosphoryl)propanoate **9** and potassium *tert*-butoxide was added to the addition funnel of the flask with ethyl (*S*)-2-(methoxymethoxy)propanoate **1** via cannula and added dropwise at -78 °C. The reaction mixture was slowly warmed to 23 °C. After stirring for 20 h at the same temperature, the reaction mixture was quenched with aqueous 1 M sodium citrate (70 mL) and stirred. After 16 h, the organic solvent was removed under reduced pressure, and the residue was extracted with

EtOAc/hexanes (1:4, 2 × 50 mL) and washed with brine (1 × 50 mL) using a separatory funnel. The combined organic layers were dried over anhydrous Na_2SO_4, filtered, and concentrated under reduced pressure. The crude material was purified by flash chromatography (2 to 10% EtOAc in hexanes) on silica gel (175 mL) to afford ethyl (*S*,*Z*)-4-(methoxymethoxy)-2-methylpent-2-enoate **2** (1.62 g, 52% yield, 6:94 E:Z) as a colorless oil. R_f = 0.30 (10% EtOAc in hexanes); IR (neat): v_{max} = 2981, 2932, 1714, 1650, 1450, 1370, 1212, 1157, 1096, 1028, 920 cm^{-1}; $[\alpha]_D^{25}$ -89.7 (*c* 1.0, CH_2Cl_2); ^1H NMR (500 MHz, 296K, $CDCl_3$) δ 5.85 (dq, *J* = 8.3, 1.5 Hz, 1H), 5.02 (m, 1H), 4.64 (d, *J* = 6.7 Hz, 1H), 4.57 (d, *J* = 6.7 Hz, 1H), 4.20 (q, *J* = 7.2 Hz, 2H), 3.35 (s, 3H), 1.92 (d, *J* = 1.5 Hz, 3H), 1.30 (t, *J* = 7.2 Hz, 3H), 1.28 (d, *J* = 5.1 Hz, 3H); ^{13}C NMR (100 MHz, 296K, $CDCl_3$) δ 167.5, 144.1, 128.3, 94.8, 70.0, 60.6, 55.4, 21.0, 20.5, 14.3; HRMS (ESI+) calcd. for $C_{10}H_{18}O_4Na$ $[M+Na]^+$ 225.1097, found 225.1087.

*(S,Z)-4-(Methoxymethoxy)-2-methylpent-2-enoic acid (**3**)*. A 50-mL round-bottom flask with ethyl (*S*,*Z*)-4-(methoxymethoxy)-2-methylpent-2-enoate **2** (1.09 g, 5.43 mmol, 6:94 E:Z), open to air, in methanol (2.9 mL) was cooled to 0 °C. Aqueous 1.0 M NaOH (13.6 mL, 2.5 equiv) was added dropwise at 0 °C. The resulting mixture was warmed to 23 °C. After stirring for 16 h at the same temperature, the mixture was concentrated under reduced pressure to remove excess methanol, and then acidified with aqueous 4 M HCl to approximately pH 4. The resulting solution was extracted with EtOAc (4 × 20 mL) and washed with brine (1 × 20 mL) using a separatory funnel. The combined organic layers were dried over anhydrous Na_2SO_4, filtered, and concentrated under reduced pressure to afford (*S*,*Z*)-4-(methoxymethoxy)-2-methylpent-2-enoic acid **3** (1.09 g, quantitative) as a colorless oil. R_f = 0.15 (30% EtOAc in hexanes); IR (neat): v_{max} = 2934, 1718, 1692, 1646, 1453, 1371, 1214, 1157, 1095, 1027, 921 cm^{-1}; $[\alpha]_D^{25}$ -85.3 (*c* 1.0, CH_2Cl_2); ^1H NMR (300 MHz, 296K, 1% CD_3OD in $CDCl_3$) δ 6.03 (dq, *J* = 8.3, 1.4 Hz, 1H), 5.09 (m, 1H), 4.67 (d, *J* = 6.8 Hz, 1H), 4.62 (d, *J* = 6.8 Hz, 1H), 3.38 (s, 3H), 1.94 (d, *J* = 1.4 Hz, 3H), 1.30 (d, *J* = 6.4 Hz, 3H); ^{13}C NMR (100 MHz, 296K, 1% CD_3OD $CDCl_3$) δ 172.3, 147.2, 127.2, 95.0, 70.4, 55.5, 20.9, 20.4. HRMS (ESI-) calcd. for $C_8H_{13}O_4$ $[M-H]^-$ 173.0808, found 173.0804.

*(S,Z)-N-((2R,3R,5S,6S)-6-Allyl-2,5-dimethyltetrahydro-2H-pyran-3-yl)-4-(methoxymethoxy)-2-methylpent-2-enamide (**4**)*. A 50-mL round-bottom flask with (2*R*,3*R*,5*S*,6*S*)-6-allyl-2,5-dimethyltetrahydro-2*H*-pyran-3-amine **10** (288 mg, 1.70 mmol) was purged with nitrogen and then charged with CH_2Cl_2 (5.7 mL), (*S*,*Z*)-4-(methoxymethoxy)-2-methylpent-2-enoic acid **3** (445 mg, 2.55 mmol, 1.5 equiv), and diisopropylethylamine (1.63 mL, 8.94 mmol, 3.5 equiv) at 23 °C. The resulting solution was cooled to 0 °C, and HATU (991 mg, 2.55 mmol, 1.5 equiv) was added at 0 °C. The mixture was warmed to 23 °C. After 42 h, the reaction mixture was quenched with aqueous satd. NH_4Cl (10 mL), extracted with EtOAc (2 × 10 mL), and washed with brine (1 × 10 mL) using a separatory funnel. The combined organic layers were dried over anhydrous Na_2SO_4, filtered, and concentrated under reduced pressure. The crude material was purified by flash chromatography (10 to 25% EtOAc in hexanes) on silica gel (70 mL) to afford (*S*,*Z*)-*N*-((2*R*,3*R*,5*S*,6*S*)-6-allyl-2,5-dimethyltetrahydro-2*H*-pyran-3-yl)-4-(methoxymethoxy)-2-methylpent-2-enamide **4** (351 mg) as an inseparable mixture of isomers as a yellow oil. The mixture of isomers was used in the next step without further purification. A small portion of the mixture

was subjected to HPLC purification for spectroscopic analysis. R_f = 0.29 (40% EtOAc in hexanes); ^{1}H NMR (500 MHz, 296K, $CDCl_3$) δ 5.85 (d, *J* = 9.0 Hz, 1H), 5.84–5.74 (m, 1H), 5.52 (dq, *J* = 8.9, 1.5 Hz, 1H), 5.11 (dq, *J* = 17.2, 1.5 Hz, 1H), 5.05 (br d, *J* = 10.0 Hz, 1H), 4.78 (dq, *J* = 8.9, 6.3 Hz, 1H), 4.67 (d, *J* = 6.7 Hz, 1H), 4.56 (d, *J* = 6.7 Hz, 1H), 3.99–3.94 (m, 1H), 3.68 (qd, *J* = 6.4, 2.1 Hz, 1H), 3.54 (ddd, *J* = 7.2, 7.2, 2.7 Hz, 1H), 3.35 (s, 3H), 2.37–2.29 (m, 1H), 2.18–2.08 (m, 1H), 1.97–1.93 (m, 5H), 1.83–1.76 (m, 1H), 1.29 (d, *J* = 6.3 Hz, 3H), 1.16 (d, *J* = 6.5 Hz, 3H), 1.03 (d, *J* = 7.4 Hz, 3H); HRMS (ESI+) calcd. for $C_{18}H_{32}NO_4$ $[M+H]^+$ 326.2326, found 326.2311.

*(S,Z)-N-((2R,3R,5S,6S)-2,5-Dimethyl-6-((E)-3-methyl-4-oxobut-2-en-1-yl)tetrahydro-2H-pyran-3-yl)-4-(methoxymethoxy)-2-methylpent-2-enamide (**5**)*. A 5-mL sealed tube with the mixture of (*S*,*Z*)-*N*-((2*R*,3*R*,5*S*,6*S*)-6-allyl-2,5-dimethyltetrahydro-2*H*-pyran-3-yl)-4-(methoxymethoxy)-2-methylpent-2-enamide **4** (216 mg, 665 mmol) was placed under a flow of argon gas and then charged with methacrolein (1.65 mL, 15.9 mmol, 24 equiv) and nitro-Grela catalyst (22 mg, 0.033 mmol, 5 mol%). The sealed tube was capped, and the reaction was heated to 50 °C (external temperature). After 20 h at 50 °C, the reaction was cooled to 23 °C. The crude contents were transferred to a separate 10-mL pear-shaped flask, concentrated under reduced pressure, and purified by flash chromatography (20 to 60% EtOAc in hexanes) on silica gel (30 mL) to afford (*S*,*Z*)-*N*-((2*R*,3*R*,5*S*,6*S*)-2,5-dimethyl-6-((*E*)-3-methyl-4-oxobut-2-en-1-yl)tetrahydro-2*H*-pyran-3-yl)-4-(methoxymethoxy)-2-methylpent-2-enamide **5** (147 mg, 39% yield, over two steps) as a yellow-brown oil. R_f = 0.31 (60% EtOAc in hexanes); IR (neat): v_{max} = 3450, 3343, 2968, 2925, 1683, 1640, 1504, 1467, 1446, 1372, 1215, 1156, 1096, 1065, 1028, 918 cm^{-1}; $[\alpha]_D^{25}$ -91.4 (*c* 1.0, CH_2Cl_2); ^{1}H NMR (500 MHz, 296K, $CDCl_3$) δ 9.42 (s, 1H), 6.57–6.51 (m, 1H), 5.94 (d, *J* = 8.6 Hz, 1H), 5.53 (dq, *J* = 8.9, 1.5 Hz, 1H), 4.78 (dq, *J* = 8.9, 6.4 Hz, 1H), 4.68 (d, *J* = 6.7 Hz, 1H), 4.57 (d, *J* = 6.7 Hz, 1H), 4.02–3.97 (m, 1H), 3.71 (qd, *J* = 6.4, 2.3 Hz, 1H), 3.66 (ddd, *J* = 8.4, 5.3, 2.8 Hz, 1H), 3.35 (s, 3H), 2.61–2.53 (m, 1H), 2.45–2.38 (m, 1H), 2.00 (app t, *J* = 3.6 Hz, 2H), 1.96 (d, *J* = 1.4 Hz, 3H), 1.87–1.79 (m, 1H), 1.76 (br s, 3H), 1.20 (d, *J* = 6.4 Hz, 3H), 1.17 (d, *J* = 6.5 Hz, 3H), 1.07 (d, *J* = 7.4 Hz, 3H); ^{13}C NMR (125 MHz, 296K, $CDCl_3$) δ 195.2, 168.6, 150.4, 140.7, 136.8, 132.6, 94.5, 79.9, 76.3, 69.7, 55.4, 47.0, 35.9, 32.9, 29.6, 21.5, 21.2, 17.9, 15.3, 9.6; HRMS (ESI+) calcd. for $C_{20}H_{34}NO_5$ $[M+H]^+$ 368.2432, found 368.2429.

*(S,Z)-N-((2R,3R,5S,6S)-2,5-Dimethyl-6-((E)-3-methylpenta-2,4-dien-1-yl)tetrahydro-2H-pyran-3-yl)-4-(methoxymethoxy)-2-methylpent-2-enamide (**6**)*. A 2-dram vial with methyltriphenylphosphonium bromide (139 mg, 0.382 mmol, 3.5 equiv) was purged with nitrogen gas three times, charged with THF (1 mL), and cooled to 0 °C. Potassium *tert*-butoxide (29 mg, 0.35 mmol, 3.2 equiv) was added at 0 °C. After 30 min at 0 °C, (*S*,*Z*)-*N*-((2*R*,3*R*,5*S*,6*S*)-2,5-dimethyl-6-((*E*)-3-methyl-4-oxobut-2-en-1-yl)tetrahydro-2*H*-pyran-3-yl)-4-(methoxymethoxy)-2-methylpent-2-enamide **5** (29.2 mg, 0.109 mmol) in THF (0.5 mL) was added, rinsed with THF (0.5 mL), and the mixture was warmed to 23 °C. After stirring for 18 h at the same temperature, the reaction was quenched with aqueous satd. NH_4Cl (1 mL). The mixture was separated, and the aqueous layer was extracted with Et_2O (2 × 2 mL) using a separatory funnel. The combined organic layers were washed with brine (1 × 2 mL), dried over anhydrous Na_2SO_4, filtered, and concentrated under reduced pressure. The crude material was purified

by flash chromatography (10 to 25% EtOAc in hexanes) on silica gel (7 mL) to afford (*S*,*Z*)-*N*-((2*R*,3*R*,5*S*,6*S*)-2,5-dimethyl-6-((*E*)-3-methylpenta-2,4-dien-1-yl)tetrahydro-2*H*-pyran-3-yl)-4-(methoxymethoxy)-2-methylpent-2-enamide **6** (23 mg, 78% yield) as a colorless oil. R_f = 0.37 (40% EtOAc in hexanes); IR (neat): ν_{max} = 3453, 3335, 2973, 2931, 1669, 1638, 1500, 1469, 1445, 1372, 1215, 1157, 1097, 1062, 1031, 918 cm^{-1}; $[\alpha]_D^{25}$ -79.8 (*c* 1.0, CH_2Cl_2); ^{1}H NMR (400 MHz, 296K, $CDCl_3$) δ 6.36 (dd, *J* = 17.3, 10.7 Hz, 1H), 5.92 (d, *J* = 8.7 Hz, 1H), 5.51 (dq, *J* = 8.9, 1.5 Hz, 1H), 5.54 (app t, *J* = 7.0 Hz, 1H), 5.11 (d, *J* = 17.3 Hz, 1H), 4.95 (d, *J* = 10.7 Hz, 1H), 4.78 (dq, *J* = 8.9, 6.4 Hz, 1H), 4.67 (d, *J* = 6.6 Hz, 1H), 4.56 (d, *J* = 6.6 Hz, 1H), 4.00–3.93 (m, 1H), 3.68 (qd, *J* = 6.4, 2.3 Hz, 1H), 3.54 (ddd, *J* = 7.3, 7.3, 2.7 Hz, 1H), 3.34 (s, 3H), 2.44–2.34 (m, 1H), 2.30–2.20 (m, 1H), 1.97–1.91 (m, 5H), 1.84–1.77 (m, 1H), 1.75 (br s, 3H), 1.29 (d, *J* = 6.4 Hz, 3H), 1.16 (d, *J* = 6.5 Hz, 3H), 1.02 (d, *J* = 7.4 Hz, 3H); ^{13}C NMR (100 MHz, 296K, $CDCl_3$) δ 168.5, 141.3, 136.7, 135.8, 132.6, 128.2, 111.3, 94.4, 80.8, 76.0, 69.7, 55.4, 47.2, 36.0, 32.0, 28.9, 21.5, 21.1, 18.0, 15.1, 12.1; HRMS (ESI+) calcd. for $C_{21}H_{36}NO_4$ $[M+H]^+$ 366.2639, found 366.2644.

2'-Me meayamycin D. A 2-mL sealed tube was treated with (*S*,*Z*)-*N*-((2*R*,3*R*,5*S*,6*S*)-2,5-dimethyl-6-((*E*)-3-methylpenta-2,4-dien-1-yl)tetrahydro-2*H*-pyran-3-yl)-4-(methoxymethoxy)-2-methylpent-2-enamide **6** (59 mg, 0.16 mmol) in DCE (315 µL). (3*R*,4*R*,5*R*)-7,7-Dimethyl-5-vinyl-1,6-dioxaspiro[2.5]octan-4-ol **7** in DCE (148 µL, 100 mg/mL solution, 0.5 equiv) and nitro-Grela catalyst (5 mg, 7 µmol, 5 mol%) were added to the sealed tube at 23 °C, and the sealed tube was purged with argon. The sealed tube was heated to 50 °C. After 2 h at 50 °C, additional right-hand fragment **7** in DCE (148 µL, 100 mg/mL solution, 0.5 equiv) was added. After an additional 2 h at 50 °C, right-hand fragment **7** in DCE (148 µL, 100 mg/mL, 0.5 equiv) and nitro-Grela catalyst (5 mg, 7 µmol, 5 mol%) were added. After an additional 4 h at 50 °C, the reaction was cooled to 23 °C and concentrated under reduced pressure. The crude material was purified by flash chromatography (20 to 70% EtOAc in hexanes) on silica gel (30 mL) to afford a complex mixture, which was further purified by preparative TLC (60% EtOAc in hexanes). Unreacted (*S*,*Z*)-*N*-((2*R*,3*R*,5*S*,6*S*)-2,5-dimethyl-6-((*E*)-3-methylpenta-2,4-dien-1-yl)tetrahydro-2*H*-pyran-3-yl)-4-(methoxymethoxy)-2-methylpent-2-enamide **6** and right-hand fragment **7** were resubmitted to the reaction conditions and purified by preparative TLC (60% EtOAc in hexanes). The product mixtures were combined, dissolved in CH_2Cl_2 (5 mL), and charcoal (1.5 g, 50× by weight) was added. After 3 h, the mixture was filtered through Celite® and the filtrate was concentrated under reduced pressure, and further purified by preparative TLC (60% EtOAc in hexanes) to afford 2'-Me meayamycin D (7 mg, 8% yield) as a tan oil. The resulting oil was further purified by semi-preparative HPLC for biological studies. R_f = 0.21 (60% EtOAc in hexanes); IR (neat): ν_{max} = 3446, 3348, 2968, 2925, 1666, 1637, 1501, 1450, 1381, 1336, 1216, 1156, 1114, 1096, 1059, 1031, 973 cm^{-1}; $[\alpha]_D^{25}$ +18.9 (*c* 0.1, CH_2Cl_2); ^{1}H NMR (500 MHz, 296K, CD_2Cl_2) δ 6.34 (d, *J* = 15.7 Hz, 1H), 5.89 (d, *J* = 8.8 Hz, 1H), 5.64 (dd, *J* = 15.7, 6.6 Hz, 1H), 5.52 (app t, *J* = 7.0 Hz, 1H), 5.46 (dq, *J* = 8.9, 1.5 Hz, 1H), 4.69 (dq, *J* = 8.9, 6.4 Hz, 1H), 4.62 (d, *J* = 6.7 Hz, 1H), 4.51 (d, *J* = 6.7 Hz, 1H), 3.96 (dd, *J* = 9.3, 6.7 Hz, 1H), 3.93–3.87 (m, 1H), 3.67 (qd, *J* = 6.4, 2.3 Hz, 1H), 3.53 (ddd, *J* = 7.8, 6.7, 2.7 Hz, 1H), 3.48 (dd, *J* = 10.1, 10.1 Hz, 1H), 3.31 (s, 3H), 2.96 (d, *J* = 4.7 Hz, 1H), 2.46 (d, *J* = 4.7 Hz, 1H), 2.40–2.32 (m, 1H), 2.26–2.18 (m,

1H), 2.17 (d, *J* = 14.3 Hz, 1H) 1.96–1.91 (m, 5H), 1.81–1.74 (m, 4H), 1.61 (d, *J* = 10.4 Hz, 1H), 1.39 (d, *J* = 14.3 Hz, 1H), 1.36 (s, 3H), 1.25–1.22 (overlapping d + s, 6H), 1.12 (d, *J* = 6.4 Hz, 3H), 1.02 (d, *J* = 7.4 Hz, 3H); ^{13}C NMR (125 MHz, 296K, CD_2Cl_2) δ 168.7, 137.8, 136.1, 135.0, 133.6, 129.5, 125.9, 94.5, 81.2, 76.2, 74.9, 73.0, 69.7, 68.6, 57.8, 55.4, 47.8, 47.5, 43.1, 36.2, 32.4, 31.1, 29.5, 23.7, 21.6, 21.1, 18.0, 15.3, 12.8; HRMS (ESI+) calcd. for $C_{29}H_{48}NO_7$ $[M+H]^+$ 522.3425, found 522.3418.

*(S)-2-(Methoxymethoxy)propanoic acid (**11**)*. The preparation of (*S*)-2-(methoxymethoxy)propanoic acid **11** followed the reported procedure.[1] We note that a 5% MeOH in CH_2Cl_2 solution or a 25% ^{i}PrOH in $CHCl_3$ solution may be used as suitable replacements for extraction when the product remains in the aqueous layer.

*(S)-N-Methoxy-2-(methoxymethoxy)-N-methylpropanamide (**12**)*. A 500-mL round-bottom flask with (*S*)-2-(methoxymethoxy)propanoic acid **11** (6.68 g, 49.8 mmol) was purged with nitrogen and then charged with CH_2Cl_2 (165 mL) and trimethylacetyl chloride (6.81 mL, 54.8 mmol, 1.1 equiv) at 23 °C. The resulting solution was cooled to 0 °C, and triethylamine (7.71 mL, 54.8 mmol, 1.1 equiv) was added at 0 °C. After 1.5 h at 0 °C, *N*,*O*-dimethylhydroxylamine hydrochloride (5.45 g, 5.48 mmol, 1.1 equiv) and triethylamine (9.82 mL, 69.7 mmol, 1.4 equiv) were added at 0 °C. The mixture was warmed to 23 °C. After stirring for 20 h at the same temperature, the mixture was diluted with EtOAc (100 mL) and washed with 1M HCl (100 mL) using a separatory funnel. The organic layer was washed with aqueous satd. $NaHCO_3$ (100 mL), and then washed with brine (100 mL). The organic layer was dried over anhydrous Na_2SO_4, filtered, and concentrated under reduced pressure. The crude material was purified by flash chromatography (40 to 70% EtOAc in hexanes) on silica gel (500 mL) to afford (*S*)-*N*-methoxy-2-(methoxymethoxy)-*N*-methylpropanamide **12** (7.31 g, 83% yield) as a pale oil. R_f = 0.20 (60% EtOAc in hexanes); IR (neat): v_{max} = 2941, 1671, 1461, 1390, 1162, 1105, 1043, 920 cm^{-1}; $[\alpha]_D^{25}$ -102.0 (*c* 1.0, CH_2Cl_2); ^{1}H NMR (300 MHz, 296K, $CDCl_3$) δ 4.68 (d, *J* = 7.0 Hz, 1H), 4.65 (d, *J* = 7.0 Hz, 1H), 4.69–4.58 (m, 1H), 3.72 (s, 3H), 3.39 (s, 3H), 3.21 (s, 3H), 1.40 (d, *J* = 6.7 Hz, 3H); ^{13}C NMR (150 MHz, 296K, $CDCl_3$) δ 173.7, 95.6, 68.9, 61.5, 55.9, 32.4, 18.3; HRMS (ESI+) calcd. for $C_7H_{16}NO_4$ $[M+H]^+$ 178.1074, found 178.1066.

*(S)-3-(Methoxymethoxy)butan-2-one (**13**)*. A 250-mL round-bottom flask equipped with a reflux condenser, with magnesium granules (1.24 g, 51.2 mmol, 3.2 equiv) was purged with nitrogen gas three times, charged with Et_2O (25 mL), and cooled to 0 °C. Iodomethane (1.5 mL, 48 mmol, 3.0 equiv) was added at 0 °C. After 30 min at 0 °C, (*S*)-*N*-methoxy-2-(methoxymethoxy)-*N*-methylpropanamide **12** (2.84 g, 16.0 mmol) in Et_2O (25 mL) was added, and stirred at 0 °C. After 2.5 h at 0 °C, the reaction was quenched with aqueous satd. NH_4Cl (50 mL). The mixture was separated, and the aqueous layer was extracted with Et_2O (2 × 25 mL) using a separatory funnel. The combined organic layers were washed with brine (1 × 25 mL), dried over anhydrous Na_2SO_4, filtered, and carefully concentrated under reduced pressure to afford (*S*)-3-(methoxymethoxy)butan-2-one **13** (1.86 g, 88% yield) as a yellow oil. R_f = 0.37 (30% EtOAc in hexanes); v_{max} = 2939, 2894, 1718, 1356, 1154, 1111, 1026, 943, 920 cm^{-1}; $[\alpha]_D^{25}$ -13.4 (*c* 1.0, $CHCl_3$); ^{1}H NMR (400 MHz, 296K, $CDCl_3$) δ 4.68 (d, *J* = 6.8 Hz, 1H), 4.63 (d, *J* = 6.8 Hz,

1H), 4.09 (q, J = 6.9 Hz, 1H), 3.37 (s, 3H), 2.18 (s, 3H), 1.32 (d, J = 6.9 Hz, 3H); ^{13}C NMR (100 MHz, 296K, $CDCl_3$) δ 210.2, 96.0, 78.6, 56.0, 25.6, 17.4. GCMS (EI+) m/z: 117 (M – CH_3), 105, 89 (M – C_2H_3O), 74 (M – C_2H_3O – CH_3), 45 (C_2H_5O) (Figure S8).

*Ethyl (S,Z)-4-(methoxymethoxy)-3-methylpent-2-enoate (**14**)*. A 50-mL round-bottom flask with ethyl 2-(bis(2-(*tert*-butyl)phenoxy)phosphoryl)acetate **20** (2.46 g, 5.68 mmol, 1.3 equiv) was purged with nitrogen gas three times and then charged with THF (6 mL) and cooled to 0 °C. Potassium *tert*-butoxide (600 mg, 5.24 mmol, 1.2 equiv) was added in one portion to the flask at 0 °C. After 30 min at 0 °C, (*S*)-3-(methoxymethoxy)butan-2-one **13** (578 mg, 4.37 mmol) in THF (4 mL) was added at 0 °C. The mixture was warmed to 23 °C. After stirring for 18 h at the same temperature, the reaction mixture was quenched with aqueous satd. NH_4Cl (8 mL). The organic solvent was removed under reduced pressure, and the residue was extracted with EtOAc (2 × 10 mL) and washed with brine (1 × 50 mL) using a separatory funnel. The combined organic layers were dried over anhydrous Na_2SO_4, filtered, and concentrated under reduced pressure. The crude material was purified by flash chromatography (1.5 to 5% EtOAc in hexanes) on silica gel (60 mL) to afford ethyl (*S*,*Z*)-4-(methoxymethoxy)-3-methylpent-2-enoate **14** (445 mg, 50% yield, 15:85 E:Z) as a colorless oil. R_f = 0.30 (10% EtOAc in hexanes); v_{max} = 2985, 2935, 1713, 1647, 1445, 1376, 1232, 1149, 1096, 1031, 920, 861 cm^{-1}; $[\alpha]_D^{25}$ -35.8 (*c* 1.0, CH_2Cl_2); ^{1}H NMR (300 MHz, 296K, $CDCl_3$) δ 5.71 (br s, 1H), 5.59 (q, J = 6.5 Hz, 1H), 4.57 (d, J = 6.6 Hz, 1H), 4.54 (d, J = 6.6 Hz, 1H), 4.14 (q, J = 7.1 Hz, 1H), 3.36 (s, 3H), 1.88 (br s, 3H), 1.29 (d, J = 6.5 Hz, 3H), 1.27 (t, J = 7.1 Hz, 3H); ^{13}C NMR (100 MHz, 296K, $CDCl_3$) δ 165.8, 160.2, 117.5, 95.2, 70.4, 60.0, 55.7, 19.9, 18.6, 14.4; HRMS (ESI+) calcd. for $C_{10}H_{18}O_4Na$ $[M+Na]^+$ 225.1097, found 225.1087.

*(S,Z)-4-(Methoxymethoxy)-3-methylpent-2-enoic acid (**15**)*. A 25-mL round-bottom flask with ethyl (*S*,*Z*)-4-(methoxymethoxy)-3-methylpent-2-enoate **14** (261 mg, 1.29 mmol), open to air, in methanol (670 µL) was cooled to 0 °C. Aqueous 1.0 M NaOH (3.2 mL, 2.5 equiv) was added dropwise at 0 °C. After 3.5 h at 0 °C, the mixture was concentrated under reduced pressure to remove excess methanol, and then acidified with aqueous 4 M HCl to approximately pH 4. The resulting solution was extracted with CH_2Cl_2 (4 × 5 mL) using a separatory funnel. The combined organic layers were dried over anhydrous Na_2SO_4, filtered, and concentrated under reduced pressure to afford (*S*,*Z*)-4-(methoxymethoxy)-3-methylpent-2-enoic acid **15** (227 mg, quantitative) as a yellow oil. R_f = 0.16 (30% EtOAc in hexanes); v_{max} = 2982, 2935, 2896, 1689, 1643, 1445, 1374, 1156, 1097, 1030, 920 cm^{-1}; $[\alpha]_D^{25}$ -83.7 (*c* 1.0, CH_2Cl_2); ^{1}H NMR (400 MHz, 296K, 1% CD_3OD in $CDCl_3$) δ 5.74 (br s, 1H), 5.54 (q, J = 6.5 Hz, 1H), 4.60 (d, J = 6.6 Hz, 1H), 4.56 (d, J = 6.6 Hz, 1H), 3.37 (s, 3H), 1.93 (d, J = 1.1 Hz, 3H), 1.31 (d, J = 6.5 Hz, 3H); ^{13}C NMR (100 MHz, 296K, $CDCl_3$) δ 170.2, 163.1, 116.8, 95.3, 70.7, 55.7, 19.9, 19.0; HRMS (ESI-) calcd. for $C_8H_{13}O_4$ $[M-H]^-$ 173.0808, found 173.0803.

*(S,Z)-N-((2R,3R,5S,6S)-2,5-Dimethyl-6-((E)-3-methyl-4-oxobut-2-en-1-yl)tetrahydro-2H-pyran-3-yl)-4-(methoxymethoxy)-3-methylpent-2-enamide (**17**)*. A 50-mL round-bottom flask with (2*R*,3*R*,5*S*,6*S*)-6-allyl-2,5-dimethyltetrahydro-2*H*-pyran-3-amine **10** (382 mg, 2.25 mmol) was purged with nitrogen, charged with CH_2Cl_2 (7.5 mL), and cooled to 0 °C. (*S*,*Z*)-4-(Methoxymethoxy)-3-methylpent-2-enoic acid **15** (450 mg, 2.58 mmol, 1.1 equiv) and diisopropylethylamine (1.20 mL, 6.76 mmol, 3.0 equiv) were added at 0 °C. After 10 min at 0 °C, HATU (1.03 g, 2.58 mmol, 1.1 equiv) was added at 0 °C. The mixture was warmed to 23 °C. After 40 h, the reaction mixture was quenched with aqueous satd. NH_4Cl (15 mL), extracted with EtOAc (2 × 15 mL), and washed with brine (1 × 15 mL) using a separatory funnel. The combined organic layers were dried over anhydrous Na_2SO_4, filtered, and concentrated under reduced pressure. The crude material was purified by flash chromatography (5 to 20% EtOAc in hexanes) on silica gel (30 mL) to afford an inseparable mixture of isomers of (*S*,*Z*)-*N*-((2*R*,3*R*,5*S*,6*S*)-6-allyl-2,5-dimethyltetrahydro-2*H*-pyran-3-yl)-4-(methoxymethoxy)-3-methylpent-2-enamide **16** (459 mg) as a yellow oil. The mixture of isomers was used in the next step without further purification.

A 10-mL sealed tube with the mixture of (*S*,*Z*)-*N*-((2*R*,3*R*,5*S*,6*S*)-6-allyl-2,5-dimethyltetrahydro-2*H*-pyran-3-yl)-4-(methoxymethoxy)-3-methylpent-2-enamide **16** (439 mg) was placed under a flow of argon gas and then charged with methacrolein (3.50 mL, 33.8 mmol, 24 equiv) and nitro-Grela catalyst (47 mg, 0.070 mmol, 5 mol%). The sealed tube was capped, and the reaction was heated to 50 °C (external temperature). After 18 h at 50 °C, the reaction mixture was cooled to 23 °C. The crude contents were transferred to a separate 10-mL pear-shaped flask, concentrated under reduced pressure, and purified by flash chromatography (20 to 60% EtOAc in hexanes) on silica gel (70 mL) to afford (*S*,*Z*)-*N*-((2*R*,3*R*,5*S*,6*S*)-2,5-dimethyl-6-((*E*)-3-methyl-4-oxobut-2-en-1-yl)tetrahydro-2*H*-pyran-3-yl)-4-(methoxymethoxy)-3-methylpent-2-enamide **17** (269 mg, 32% yield, over two steps) as a yellow-brown oil. R_f = 0.25 (60% EtOAc in hexanes); IR (neat): ν_{max} = 3348, 2976, 2932, 1684, 1664, 1640, 1509, 1469, 1444, 1376, 1216, 1157, 1097, 1069, 1033, 919 cm^{-1}; $[\alpha]_D^{25}$ -33.3 (*c* 1.0, CH_2Cl_2); ^1H NMR (400 MHz, 296K, $CDCl_3$) δ 9.42 (s, 1H), 6.58–6.49 (m, 1H), 5.71 (d, *J* = 8.9 Hz, 1H), 5.63 (br s, 1H), 5.61 (q, *J* = 6.5 Hz, 1H), 4.58 (app s, 2H), 3.97–3.90 (m, 1H), 3.72–3.60 (m, 2H), 3.36 (s, 3H), 2.62–2.51 (m, 1H), 2.46–2.35 (m, 1H), 2.00–1.94 (m, 2H), 1.84 (d, *J* = 1.0 Hz, 3H), 1.84–1.78 (m, 1H), 1.76 (br s, 3H), 1.32 (d, *J* = 6.5 Hz, 3H), 1.14 (d, *J* = 6.4 Hz, 3H), 1.06 (d, *J* = 7.4 Hz, 3H); ^{13}C NMR (125 MHz, 296K, $CDCl_3$) δ 195.2, 165.5, 154.7, 150.5, 140.7, 120.3, 95.1, 79.9, 76.4, 70.4, 55.7, 46.9, 35.9, 33.0, 29.7, 20.0, 18.3, 17.9, 15.4, 9.6; HRMS (ESI+) calcd. for $C_{20}H_{34}NO_5$ $[M+H]^+$ 368.2431, found 368.2427.

*(S,Z)-N-((2R,3R,5S,6S)-2,5-Dimethyl-6-((E)-3-methylpenta-2,4-dien-1-yl)tetrahydro-2H-pyran-3-yl)-4-(methoxymethoxy)-3-methylpent-2-enamide (**18**)*. A 10-mL round-bottom flask with methyltriphenylphosphonium bromide (798 mg, 2.19 mmol, 3.5 equiv) was purged with nitrogen gas three times, charged with THF (2 mL), and cooled to 0 °C. Potassium *tert*-butoxide (229 mg, 2.00 mmol, 3.2 equiv) was added at 0 °C. After 30 min at 0 °C, (*S*,*Z*)-*N*-((2*R*,3*R*,5*S*,6*S*)-2,5-dimethyl-6-((*E*)-3-methyl-4-oxobut-2-en-1-yl)tetrahydro-2*H*-pyran-3-yl)-4-(methoxymethoxy)-3-methylpent-2-enamide **17** (230 mg, 0.62 mmol) in THF (1 mL) was added, rinsed with THF (1 mL), and stirred 0 °C. After stirring for 1.5 h at 0 °C, the reaction was quenched with aqueous satd. NH_4Cl (5

mL). The mixture was separated, and the aqueous layer was extracted with EtOAc (2 × 5 mL) using a separatory funnel. The combined organic layers were washed with brine (1 × 5 mL), dried over anhydrous Na_2SO_4, filtered, and concentrated under reduced pressure. The crude material was purified by flash chromatography (10 to 30% EtOAc in hexanes) on silica gel (30 mL) to afford (*S*,*Z*)-*N*-((2*R*,3*R*,5*S*,6*S*)-2,5-dimethyl-6-((*E*)-3-methylpenta-2,4-dien-1-yl)tetrahydro-2*H*-pyran-3-yl)-4-(methoxymethoxy)-3-methylpent-2-enamide **18** (188 mg, 82% yield) as a colorless oil. R_f = 0.18 (30% EtOAc in hexanes); IR (neat): ν_{max} = 3345, 2975, 2930, 1663, 1635, 1503, 1468, 1444, 1376, 1217, 1158, 1096, 1072, 1063, 1033, 919 cm^{-1}; $[\alpha]_D^{25}$ -77.4 (*c* 1.0, CH_2Cl_2); ^{1}H NMR (500 MHz, 296K, $CDCl_3$) δ 6.37 (dd, *J* = 17.4, 10.7 Hz, 1H), 5.72 (d, *J* = 9.0 Hz, 1H), 5.62 (q, *J* = 6.6 Hz, 1H), 5.61 (br s, 1H), 5.46 (app t, *J* = 7.1 Hz, 1H), 5.11 (d, *J* = 17.4 Hz, 1H), 4.95 (d, *J* = 10.7 Hz, 1H), 4.58 (app s, 2H), 3.94–3.88 (m, 1H), 3.65 (qd, *J* = 6.5, 2.2 Hz, 1H), 3.53 (ddd, *J* = 7.3, 7.3, 2.8 Hz, 1H), 3.36 (s, 3H), 2.43–2.34 (m, 1H), 2.29–2.20 (m, 1H), 1.95–1.90 (m, 2H), 1.84 (d, *J* = 1.3 Hz, 1H), 1.81–1.76 (m, 1H), 1.76 (br s, 3H), 1.31 (d, *J* = 6.6 Hz, 3H), 1.13 (d, *J* = 6.5 Hz, 3H), 1.02 (d, *J* = 7.4 Hz, 3H); ^{13}C NMR (150 MHz, 296K, $CDCl_3$) δ 165.5, 154.4, 141.4, 135.8, 128.3, 120.4, 111.3, 95.1, 80.9, 76.2, 70.4, 55.7, 47.1, 36.1, 32.1, 29.1, 20.0, 18.3, 18.0, 15.3, 12.1; HRMS (ESI+) calcd. for $C_{21}H_{36}NO_4$ $[M+H]^+$ 366.2639, found 366.2624.

3'-Me meayamycin D. A 5-mL sealed tube was treated with (*S*,*Z*)-*N*-((2*R*,3*R*,5*S*,6*S*)-2,5-dimethyl-6-((*E*)-3-methylpenta-2,4-dien-1-yl)tetrahydro-2*H*-pyran-3-yl)-4-(methoxymethoxy)-3-methylpent-2-enamide **18** (80 mg, 0.22 mmol) in DCE (429 µL). (3*R*,4*R*,5*R*)-7,7-Dimethyl-5-vinyl-1,6-dioxaspiro[2.5]octan-4-ol **7** in DCE (203 µL, 100 mg/mL solution, 0.5 equiv) and nitro-Grela catalyst (10 mg, 15 µmol, 6.7 mol%) were added to the sealed tube at 23 °C, and the sealed tube was purged with argon. The sealed tube was heated to 45 °C. After 1 h at 45 °C, additional right-hand fragment **7** in DCE (203 µL, 100 mg/mL solution, 0.5 equiv) and nitro-Grela catalyst (10 mg, 15 µmol, 6.7 mol%) were added. After an additional 1 h at 45 °C, the final portion of right-hand fragment **7** in DCE (203 µL, 100 mg/mL, 0.5 equiv) and nitro-Grela catalyst (10 mg, 7 µmol, 6.7 mol%) was added. After 12 h at 45 °C, the reaction was cooled to 23 °C and concentrated under reduced pressure. The crude material was filtered through a plug of silica and rinsed with 80% EtOAc in hexanes. Charcoal (1.7 g, 10× by weight) was added to the mixture and the resulting mixture was heated to 45 °C and stirred at that temperature. After 3 h at 45 °C, the mixture was cooled to 23 °C, filtered through Celite®, and concentrated under reduced pressure. The crude material was purified by flash chromatography (20 to 70% EtOAc in hexanes) on silica gel (20 mL) to afford a mixture, which was further purified by preparative TLC (60% EtOAc in hexanes) to afford 3'-Me meayamycin D (9 mg, 8% yield) as a tan solid. The resulting solid was further purified by semi-preparative HPLC for biological studies. R_f = 0.20 (60% EtOAc in hexanes); IR (neat): ν_{max} = 3368, 2974, 2926, 1663, 1637, 1506, 1468, 1444, 1381, 1216, 1158, 1114, 1096, 1060, 1035, 973 cm^{-1}; $[\alpha]_D^{25}$ -35.1 (*c* 0.25, CH_2Cl_2); ^{1}H NMR (600 MHz, 296K, CD_2Cl_2) δ 6.34 (d, *J* = 15.7 Hz, 1H), 5.71 (d, *J* = 9.0 Hz, 1H), 5.64 (dd, *J* = 15.6, 6.6 Hz, 1H), 5.63 (br s, 1H), 5.59 (q, *J* = 6.5 Hz, 1H), 5.52 (app t, *J* = 7.1 Hz, 1H), 4.52 (app s, 2H), 3.96 (dd, *J* = 9.4, 6.7 Hz, 1H), 3.88–3.82 (m, 1H), 3.64 (qd, *J* = 6.4, 2.2 Hz, 1H), 3.52 (ddd, *J* = 7.9, 6.6, 2.8 Hz, 1H), 3.48 (dd, *J* = 10.0, 10.0 Hz, 1H), 3.33 (s, 3H), 2.95 (d, *J* = 4.7 Hz, 1H), 2.46 (d, *J* = 4.7 Hz, 1H), 2.39–2.32 (m, 1H), 2.25–2.19 (m, 1H), 2.17 (d, *J* = 14.3

Hz, 1H) 1.92–1.88 (m, 2H), 1.81 (d, J = 1.3 Hz, 3H), 1.78 (br s, 3H), 1.78–1.73 (m, 1H), 1.61 (d, J = 10.4 Hz, 1H), 1.39 (d, J = 14.3 Hz, 1H), 1.36 (s, 3H), 1.26 (d, J = 6.5 Hz, 3H), 1.23 (s, 3H), 1.09 (d, J = 6.4 Hz, 3H), 1.01 (d, J = 7.4 Hz, 3H); ^{13}C NMR (150 MHz, 296K, CD_2Cl_2) δ 165.5, 154.3, 137.8, 134.9, 129.6, 125.8, 120.8, 95.1, 81.2, 76.4, 74.9, 73.0, 70.3, 68.6, 57.8, 55.6, 47.8, 47.3, 43.1, 36.3, 32.4, 31.1, 29.6, 23.7, 20.0, 18.2, 18.0, 15.3, 12.8; HRMS (ESI+) calcd. for $C_{29}H_{48}NO_7$ $[M+H]^+$ 522.3425, found 522.3430.

*(S,Z)-4-(Methoxymethoxy)-2-methylpent-2-en-1-ol (**8**)*. A 10-mL round-bottom flask with ethyl (*S*,*Z*)-4-(methoxymethoxy)-2-methylpent-2-enoate **2** (29 mg, 14 mmol, 13:87 E:Z) was purged with nitrogen and charged with THF (520 µL). The mixture was cooled to -78 °C, and DIBALH (1.0 M in hexanes, 472 µL, 3.3 equiv) was added at -78 °C. After stirring for 1.5 h at -78 °C, the reaction mixture was quenched with aqueous satd. NH_4Cl (3 mL) and concentrated under reduced pressure to remove THF. The remaining mixture was extracted with EtOAc (3 × 3 mL) and washed with brine (1 × 3 mL) using a separatory funnel. The combined organic layers were dried over anhydrous Na_2SO_4, filtered, and concentrated under reduced pressure. The crude material was purified by flash chromatography (10 to 40% EtOAc in hexanes) on silica gel (10 mL) to afford (*S*,*Z*)-4-(methoxymethoxy)-2-methylpent-2-en-1-ol **8** (17 mg, 68% yield) as a colorless oil. R_f = 0.30 (40% EtOAc in hexanes); v_{max} = 3416, 2973, 2932, 1449, 1374, 1157, 1095, 1027, 918 cm^{-1}; $[\alpha]_D^{25}$ -174.0 (*c* 1.0, CH_2Cl_2); ^{1}H NMR (400 MHz, 296K, 1% CD_3OD in $CDCl_3$) δ 5.13 (br d, J = 9.6 Hz, 1H), 4.73 (d, J = 6.9 Hz, 1H), 4.55 (dq, J = 9.7, 6.3 Hz, 1H), 4.51 (d, J = 6.9 Hz, 1H), 4.31 (d, J = 12.1 Hz, 1H), 3.80 (d, J = 12.1 Hz, 1H), 3.37 (s, 3H), 1.84 (d, J = 1.3 Hz, 3H), 1.23 (d, J = 6.3 Hz, 3H); ^{13}C NMR (100 MHz, 296K, 1% CD_3OD in $CDCl_3$) δ 139.8, 128.8, 93.1, 67.1, 61.5, 55.2, 22.0, 21.7; HRMS (ESI+) calcd. for $C_8H_{16}O_3Na$ $[M+Na]^+$ 183.0992, found 183.0984.

*(S,Z)-4-(Methoxymethoxy)-3-methylpent-2-en-1-ol (**19**)*. A 10-mL round-bottom flask with ethyl (*S*,*Z*)-4-(methoxymethoxy)-3-methylpent-2-enoate **14** (30 mg, 15 mmol, 39:61 E:Z) was purged with nitrogen and charged with THF (494 µL). The mixture was cooled to -78 °C, and DIBALH (1.0 M in hexanes, 445 µL, 3.0 equiv) was added at -78 °C. After stirring for 2 h at -78 °C, the reaction mixture was quenched with aqueous satd. NH_4Cl (3 mL) and concentrated under reduced pressure to remove THF. The remaining mixture was extracted with EtOAc (2 × 3 mL) and washed with brine (1 × 3 mL) using a separatory funnel. The combined organic layers were dried over anhydrous Na_2SO_4, filtered, and concentrated under reduced pressure. The crude material was purified by flash chromatography (7.5 to 30% EtOAc in hexanes) on silica gel (10 mL) to afford (*S*,*Z*)-4-(methoxymethoxy)-3-methylpent-2-en-1-ol **19** (19 mg, 79% yield, 38:62 E:Z) as a colorless oil. A small portion of the mixture was repurified by flash chromatography for spectroscopic analysis. Data: R_f = 0.24 (40% EtOAc in hexanes); v_{max} = 3408, 2980, 2933, 1447, 1378, 1157, 1096, 1031, 919 cm^{-1}; $[\alpha]_D^{25}$ -126.9 (*c* 1.0, CH_2Cl_2); ^{1}H NMR (400 MHz, 296K, $CDCl_3$) δ 5.68 (app t, J = 7.9 Hz, 1H), 4.69 (q, J = 6.6 Hz, 1H), 4.59 (d, J = 6.8 Hz, 1H), 4.50 (d, J = 6.8 Hz, 1H), 4.28 (dd, J = 12.4, 8.6 Hz, 1H), 3.90 (dd, J = 12.4, 6.4 Hz, 1H), 3.37 (s, 3H), 1.68 (br s, 3H), 1.26 (d, J = 6.6 Hz, 3H); ^{13}C NMR (100 MHz, 296K, $CDCl_3$) δ 138.9, 128.3, 93.3, 68.2, 57.5, 55.3, 19.3, 17.3; HRMS (ESI+) calcd. for $C_8H_{16}O_3Na$ $[M+Na]^+$ 183.0992, found 183.0983.

Growth Inhibition Assay

All cell lines were obtained from ATCC (Manassas, VA) and maintained in RPMI-1640 media, Waymouth media (DMS53 and DMS114 cells) + 10% (v/v) fetal bovine serum. Cells were mycoplasma-free as determined by the e-Myco PLUS mycoplasma PCR detection kit (Bulldog Bio, Portsmouth, NH). Cells were plated in 96-well plates at an initial density of 1500 or 5000 cells per well in culture media (100 μL) and were incubated for 24 h prior to compound addition. The compounds were prepared separately as 10 mM in 100% DMSO or 10 μM in 100% DMSO. Serial dilution in sterile water gave 10× dilutions that were added directly to the cells as 100-fold dilutions to give the desired concentration of compound, 0.1 nM – 30 μM meayamycin A, meayamycin D, 2'-Me meayamycin D or 3'-Me meayamycin D, in 0.1–0.3% (v/v) DMSO. The cells were then incubated for an additional 72 h. Cell proliferation was measured by using the commercial 3-(4,5-dimethylthiazol-2-yl)-5-(3-carboxymethoxyphenyl)-2-(4-sulfophenyl)-2*H*-tetrazolium (MTS) dye or 4-[3-(4-iodophenyl)-2-(4-nitrophenyl)-2*H*-5-tetrazolio]-1,3-benzene sulfonate (WST-1) dye reduction assay. The absorbance at 490 nm (MTS) or 450 nm (WST-1) was measured with a Modulus II Microplate Multimode Reader (Promega) or Tecan Infinite M1000 PRO Multimode Reader. Evaluation of the compounds was performed in duplicate at each concentration. GraphPad Prism 9.4.0 was used to construct dose-response curves and calculate the GI_{50} values.

Immunoblot Analysis

HCT116 cells were treated with various concentrations of meayamycin D analogs for 8 h and then lysed using RIPA buffer (10 mM Tris, pH 7.5, 150 mM NaCl, 1 mM EDTA, 0.1% (w/v) SDS, 1% (v/v) IGEPAL, 0.5% (w/v) sodium deoxycholate) containing phosphatase and protease inhibitors. Approximately 20 μg protein from each cell lysate was resolved on SDS-PAGE gels (Cat#5671084; Bio-Rad). Proteins were transferred onto nitrocellulose membranes followed by 1 h incubation at room temperature in blocking solution (1× TBS, 0.1% (v/v) Tween-20, 5% (w/v) milk powder). Membranes were incubated overnight at 4°C with the following antibodies: anti-phospho-SF3B1(#25009; Cell Signaling), anti-SF3B1 (#14434; Cell Signaling), anti-MCL-1 (#5453; Cell Signaling), anti-p27 (#3686; Cell Signaling), and anti-α-tubulin (#2125; Cell Signaling). Proteins were detected using SuperSignal West Pico substrate (Pierce; Rockford, IL).

Reverse Transcription-Polymerase Chain Reaction (RT-PCR)

HCT116 cells were treated with various concentrations of meayamycin D analogs for 8 h and the total RNA was extracted using RNeasy kit (Cat#74104; QIAGEN). cDNA was generated by reverse transcription using approximately 500 ng of total RNA and OneTaq® RT-PCR kit (Cat# E5310S; New England Biolabs). The primer sequences are as previously described.[2,3] For PCR, the thermocycler program for Mcl-1 and β-actin involved an initial denaturation at 95 °C for 30 sec, 35 cycles at 94 °C for 30 sec, 58 °C for 30 sec, 68 °C for 50 sec, and a final elongation at 68 °C for 7 min. The PCR products were examined on 1.5% agarose gels containing 0.5 $\mu g \times mL^{-}$

[1] ethidium bromide and imaged using a ChemiDoc system (BioRad). Images were processed using Lab Imager software (BioRad).

In Vitro Plasma Stability

Mouse CD1 plasma K2 EDTA (Innovative Research) was prepared in a 2-mL microcentrifuge tube. The compounds were prepared separately as 1 mM solutions in 10% (v/v) DMSO and added to the plasma as 100× dilutions to give 700 µL at a concentration of compound, 10 µM procaine, meayamycin A, 2'-Me meayamycin D, or 3'-Me meayamycin D in 0.1% (v/v) DMSO. The mixture was vortexed for 10 sec, capped, and placed in a shaking incubator (Corning) for 48 h at 125 rpm at 37 °C. At the indicated times an aliquot (70 µL) of the mixture was taken and added to an equal volume of ice-cold MeCN and centrifuged for 15 min at 14000 relative centrifugal force (RCF) at 4 °C. The supernatant was collected and frozen at -80 °C until sample analysis. The samples were analyzed by LC-MS using Fmoc-L-phenylalanine as an internal standard at a concentration of 25 µM. A standard curve was prepared separately in a matrix-matched solution by 2-fold serial dilution from 10 µM. The decomposition was determined by comparing the ratio of analyte to Fmoc-L-phenylalanine with the ratio of the analyte to Fmoc-L-phenylalanine in the first data point.

Preparation of Yeast Strains

Parental *Saccharomyces cerevisiae* strains and plasmids are as described in previous reports.[4,5] Hs5-16 point mutations were generated via site-directed mutagenesis and validated through DNA sequencing. Plasmids containing the mutant Hs5-16 protein genes were then introduced into yeast by plasmid shuffling and loss of the wild-type Hsh155 plasmid selected for using 5-FOA.[6] In strains containing the SF3B1 chimeras, the chimera is the only source of functional Hsh155/SF3B1 protein. Standard yeast growth media and conditions were employed unless stated otherwise.

Microplate Yeast Growth Assay

Yeast growth was done as previously described.[5] Strains were grown overnight in -tryptophan dropout media with 1% (v/v) DMSO at 30 °C while shaking (220 rpm). Cells were then diluted to an OD_{600} = 0.1 in -tryptophan dropout media. An aliquot of the diluted cultures (100 µL) was plated in a Corning Costar 96-well clear round-bottom cell culture plate. The compounds were added directly as 10-fold dilutions to give the desired concentration of compound, 1 nM – 1 µM meayamycin D, 2'-Me meayamycin D, or 3'-Me meayamycin D, in 1% (v/v) DMSO at OD_{600} = 0.1. The plates were covered with Breathe-Easy® plate sealing membranes to minimize evaporation and placed in a Tecan Infinite® 200 PRO plate reader set at 30 °C while shaking (220 rpm) for 24 h. OD_{600} measurements were read every 15 min. Absorbance values were corrected using measurements from wells that contained only media.

References

(1) Bhalay, G.; Clough, S.; McLaren, L.; Sutherland, A.; Willis, C. L. Synthesis and Enzyme-Catalysed Reductions of 2-Oxo Acids with Oxygen Containing Side-Chains. *J. Chem. Soc., Perkin Trans. 1* **2000**, No. 6, 901–910.

(2) Gao, Y.; Koide, K. Chemical Perturbation of Mcl-1 Pre-mRNA Splicing to Induce Apoptosis in Cancer Cells. *ACS Chem. Biol.* **2013**, *8* (5), 895–900.

(3) Gao, Y.; Trivedi, S.; Ferris, R. L.; Koide, K. Regulation of HPV16 E6 and MCL1 by SF3B1 Inhibitor in Head and Neck Cancer Cells. *Sci. Rep.* **2014**, *4* (1), 6098.

(4) Carrocci, T. J.; Paulson, J. C.; Hoskins, A. A. Functional Analysis of Hsh155/SF3b1 Interactions with the U2 snRNA/Branch Site Duplex. *RNA* **2018**, *24* (8), 1028–1040.

(5) Hansen, S. R.; Nikolai, B. J.; Spreacker, P. J.; Carrocci, T. J.; Hoskins, A. A. Chemical Inhibition of Pre-mRNA Splicing in Living *Saccharomyces cerevisiae*. *Cell Chem. Biol.* **2019**, *26* (3), 443–448.e3.

(6) Amberg, D. C.; Burke, D. J. Classical Genetics with *Saccharomyces cerevisiae*. *Cold Spring Harb. Protoc.* **2016**, *2016* (5), pdb.top077628.

NMR Spectra

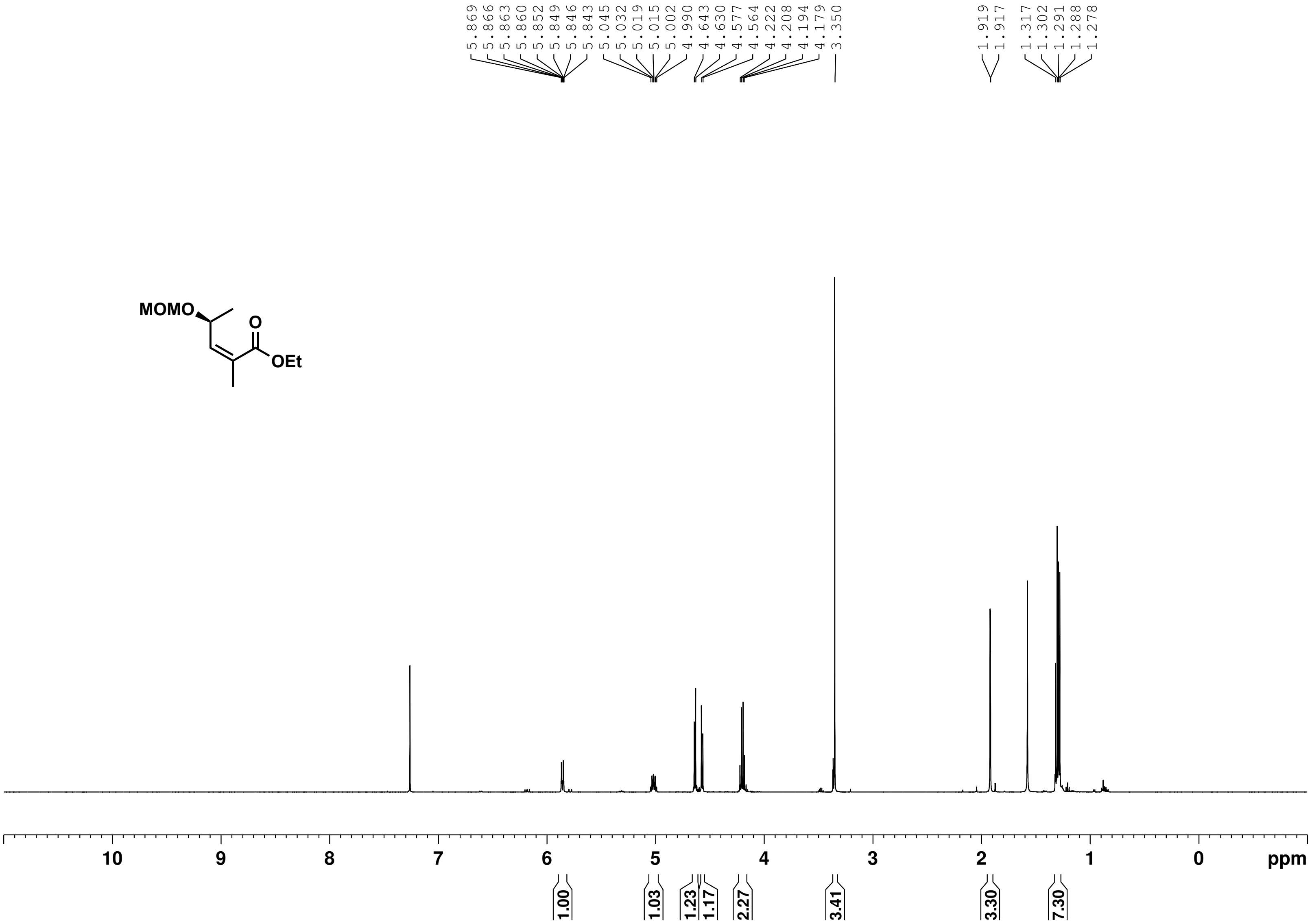

^{1}H NMR spectrum of ethyl (*S*,*Z*)-4-(methoxymethoxy)-2-methylpent-2-enoate **2** (500 MHz, $CDCl_3$, 296K)

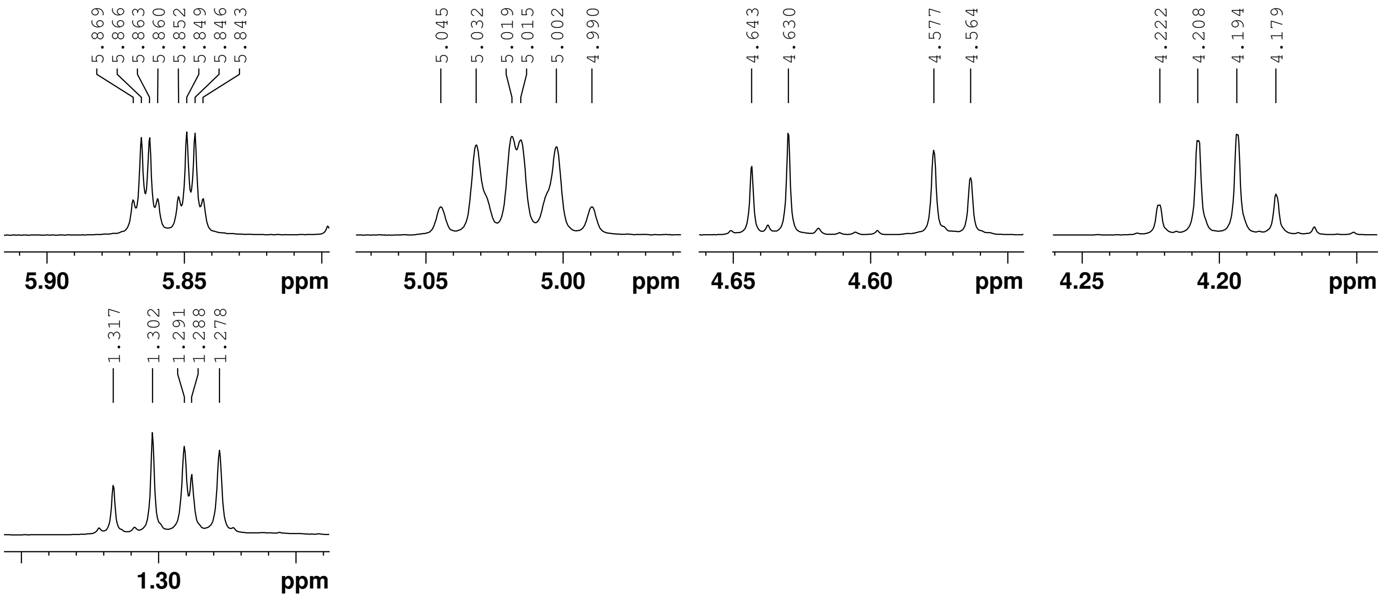

^{1}H NMR expansion of ethyl (*S*,*Z*)-4-(methoxymethoxy)-2-methylpent-2-enoate **2** (500 MHz, $CDCl_3$, 296K)

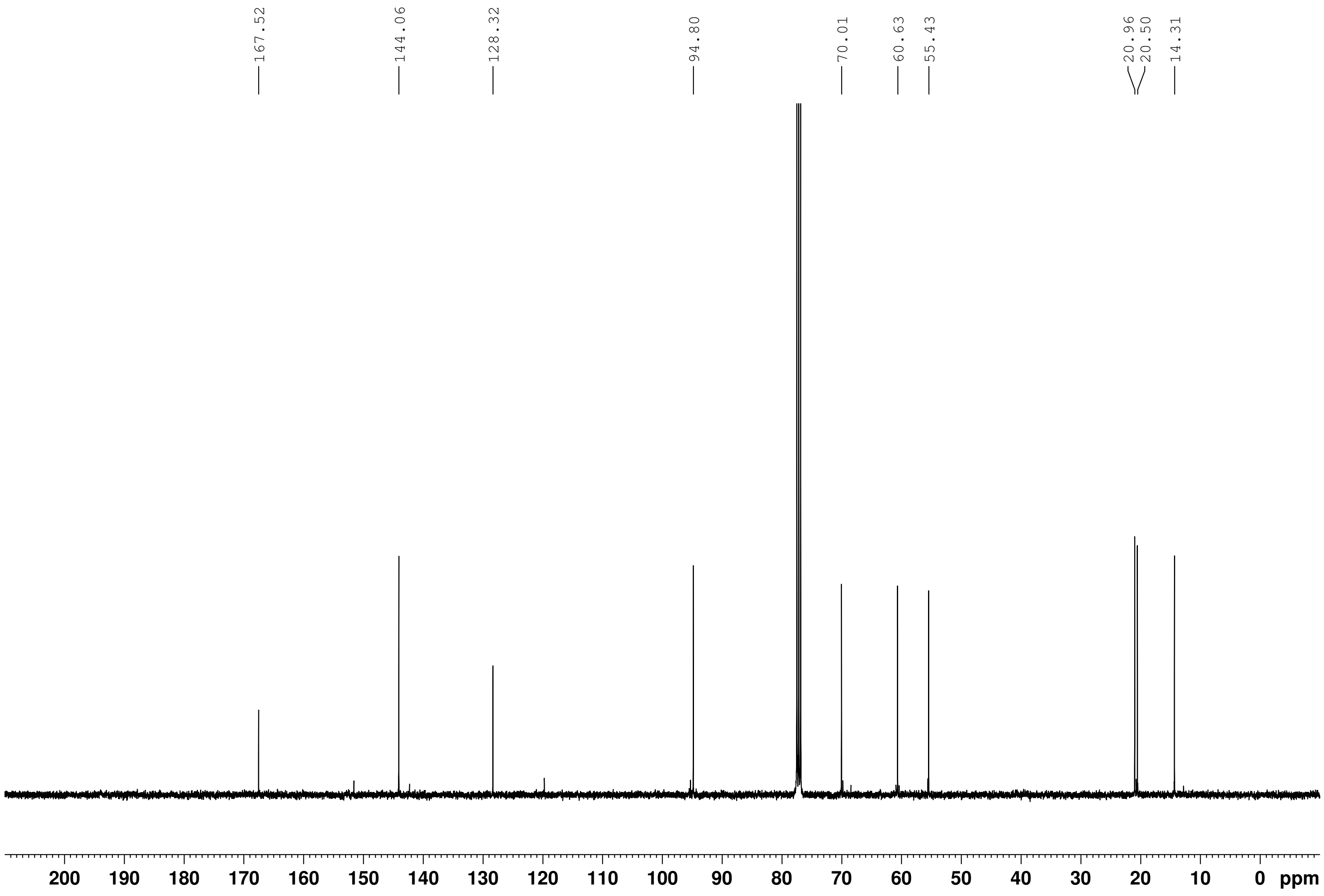

$^{13}C\{^1H\}$ NMR spectrum of ethyl (*S*,*Z*)-4-(methoxymethoxy)-2-methylpent-2-enoate **2** (100 MHz, $CDCl_3$, 296K)

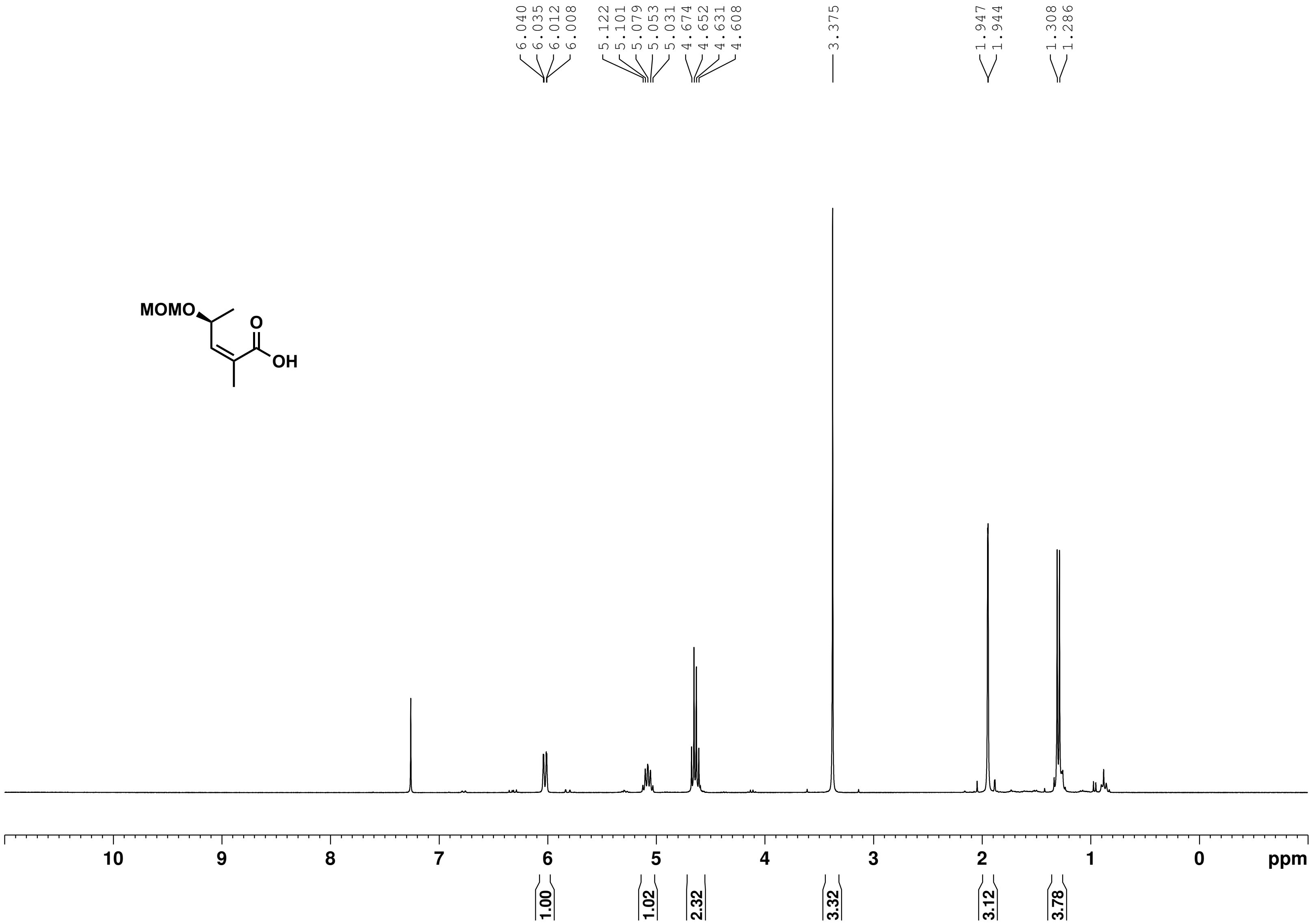

^{1}H NMR spectrum of (*S*,*Z*)-4-(methoxymethoxy)-2-methylpent-2-enoic acid **3** (300 MHz, 1% CD_3OD in $CDCl_3$, 296K)

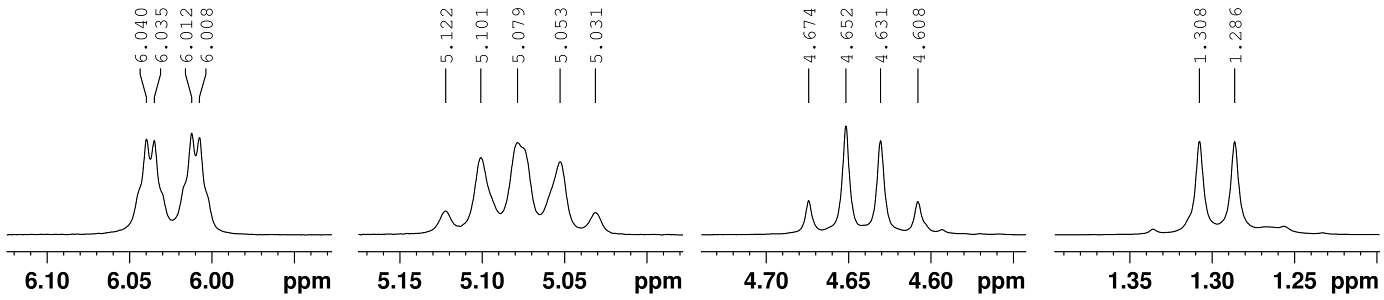

^{1}H NMR expansion of (*S*,*Z*)-4-(methoxymethoxy)-2-methylpent-2-enoic acid **3** (300 MHz, 1% CD_3OD in $CDCl_3$, 296K)

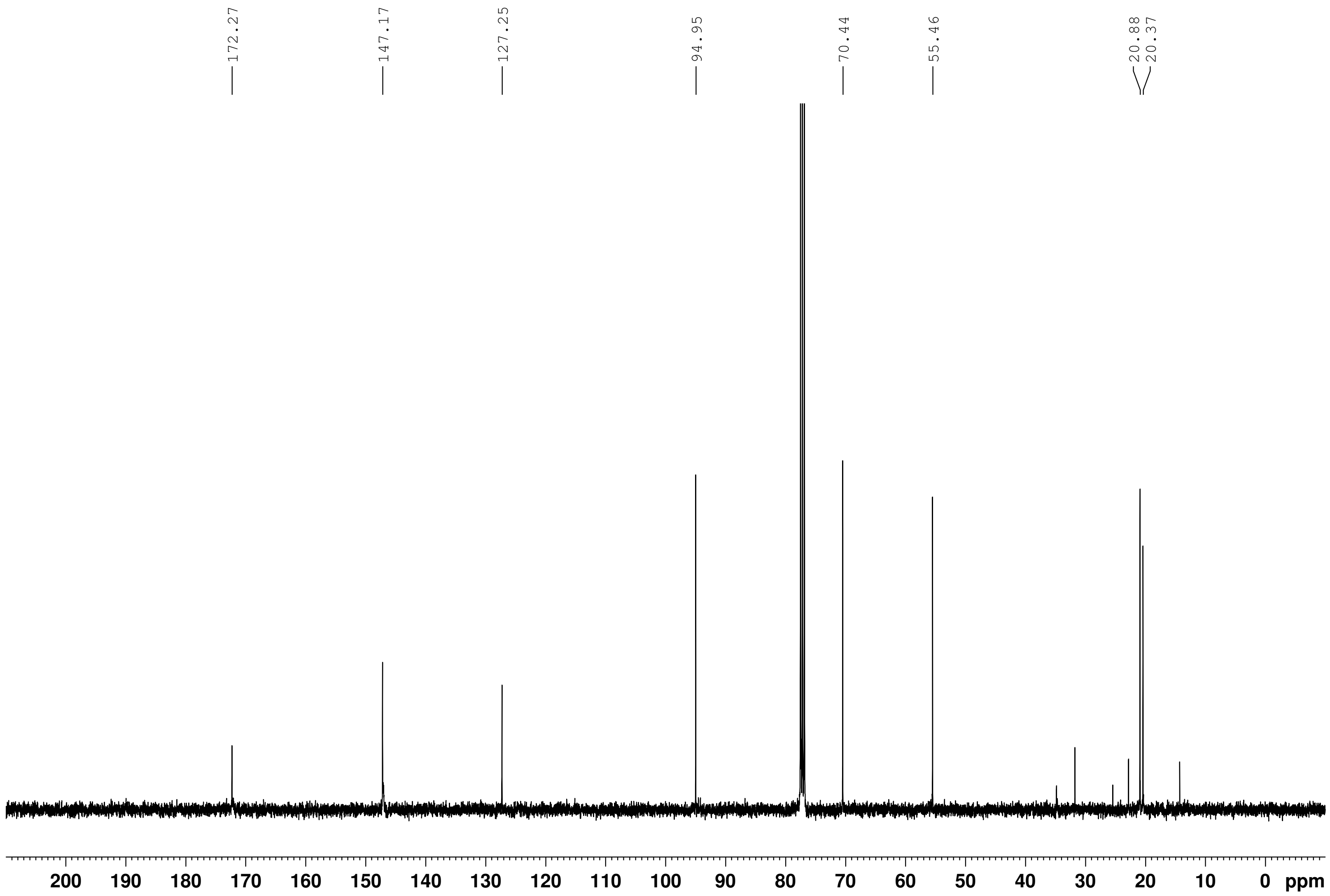

$^{13}C\{^{1}H\}$ NMR spectrum of (*S*,*Z*)-4-(methoxymethoxy)-2-methylpent-2-enoic acid **3** (100 MHz, 1% CD_3OD in $CDCl_3$, 296K)

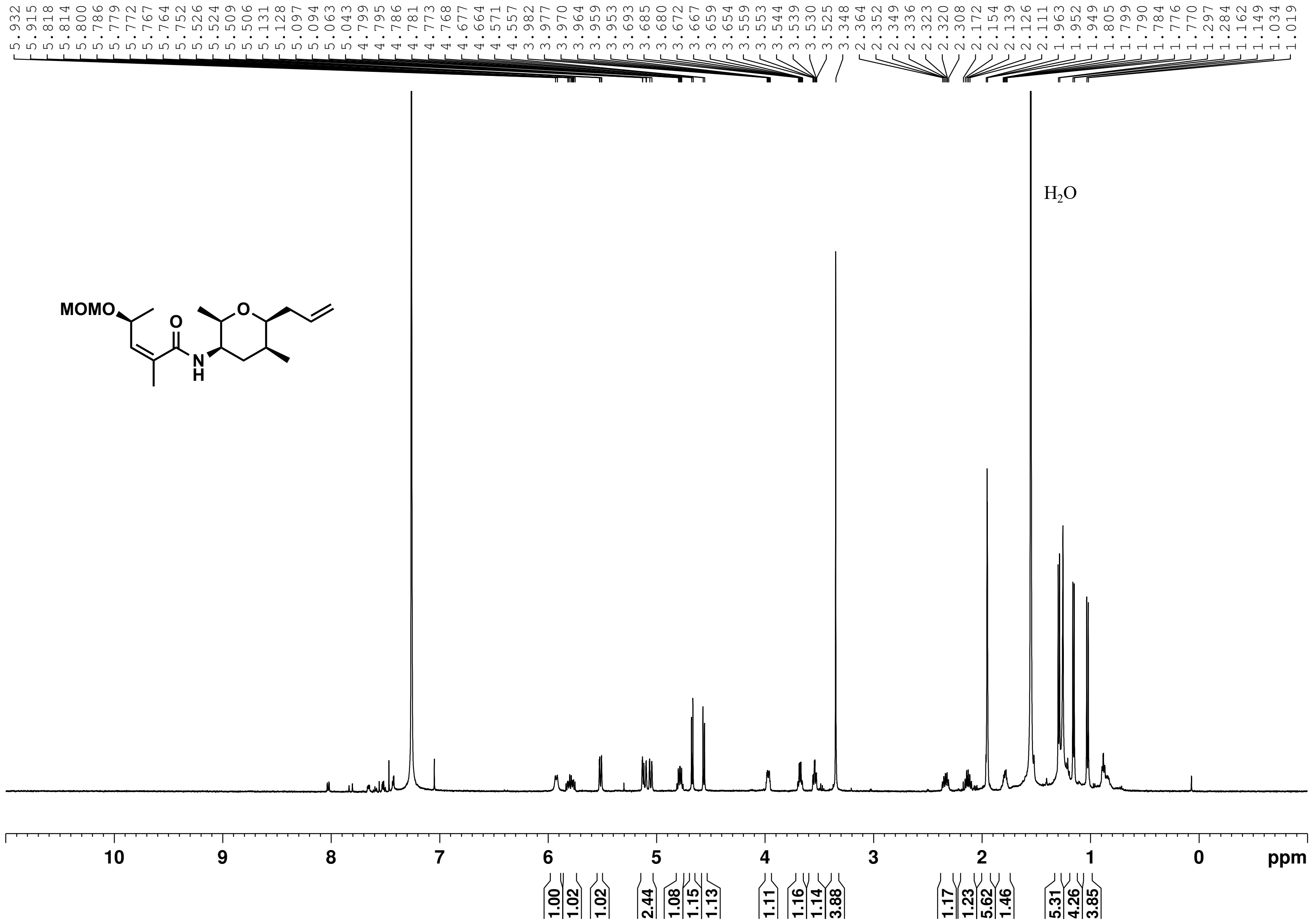

^{1}H NMR spectrum of (*S*,*Z*)-*N*-((2*R*,3*R*,5*S*,6*S*)-6-allyl-2,5-dimethyltetrahydro-2*H*-pyran-3-yl)-4-(methoxymethoxy)-2-methylpent-2-enamide **4**
(500 MHz, $CDCl_3$, 296K)

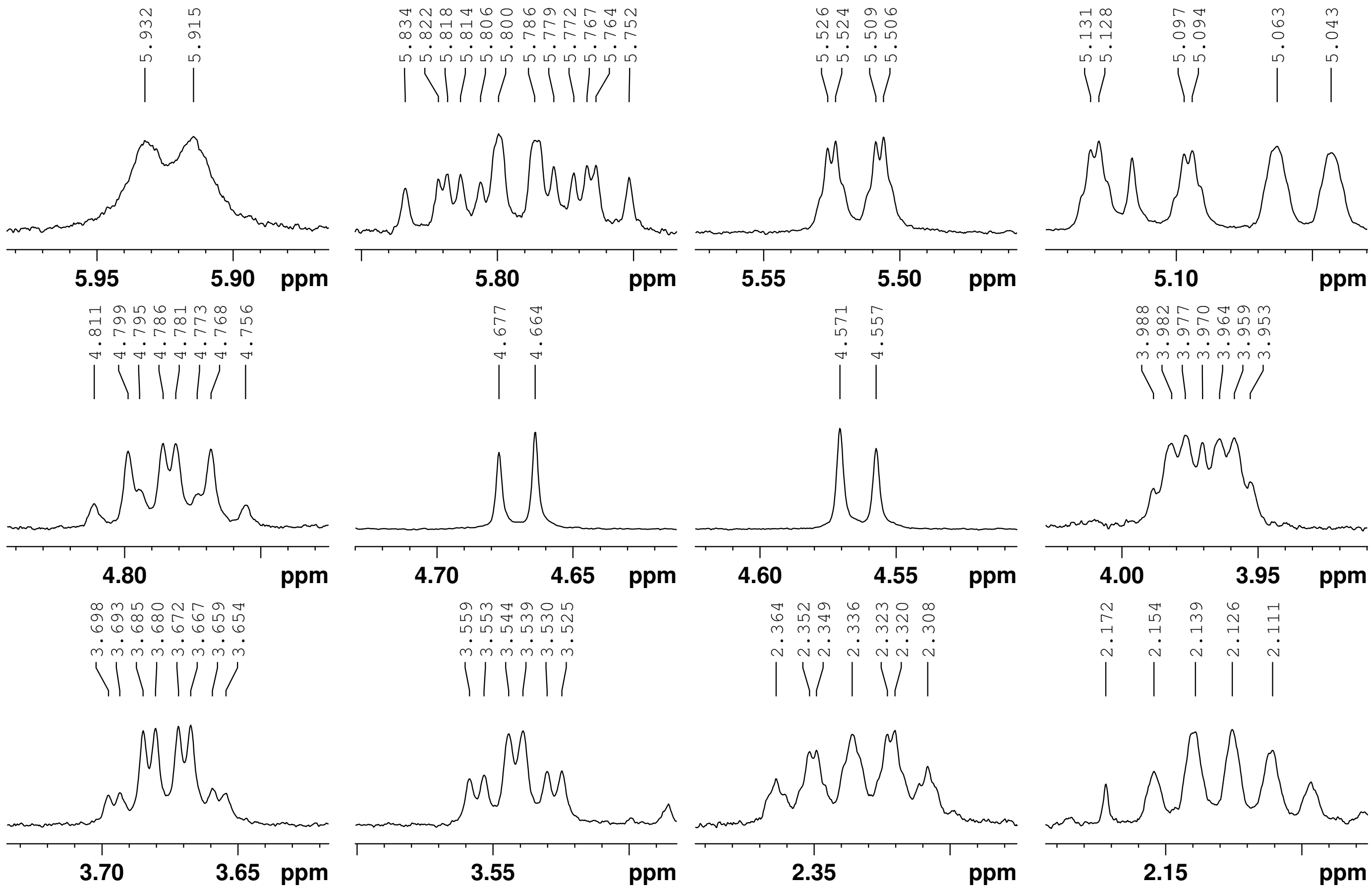

^{1}H NMR expansion of (*S*,*Z*)-*N*-((2*R*,3*R*,5*S*,6*S*)-6-allyl-2,5-dimethyltetrahydro-2*H*-pyran-3-yl)-4-(methoxymethoxy)-2-methylpent-2-enamide **4**
(500 MHz, $CDCl_3$, 296K)

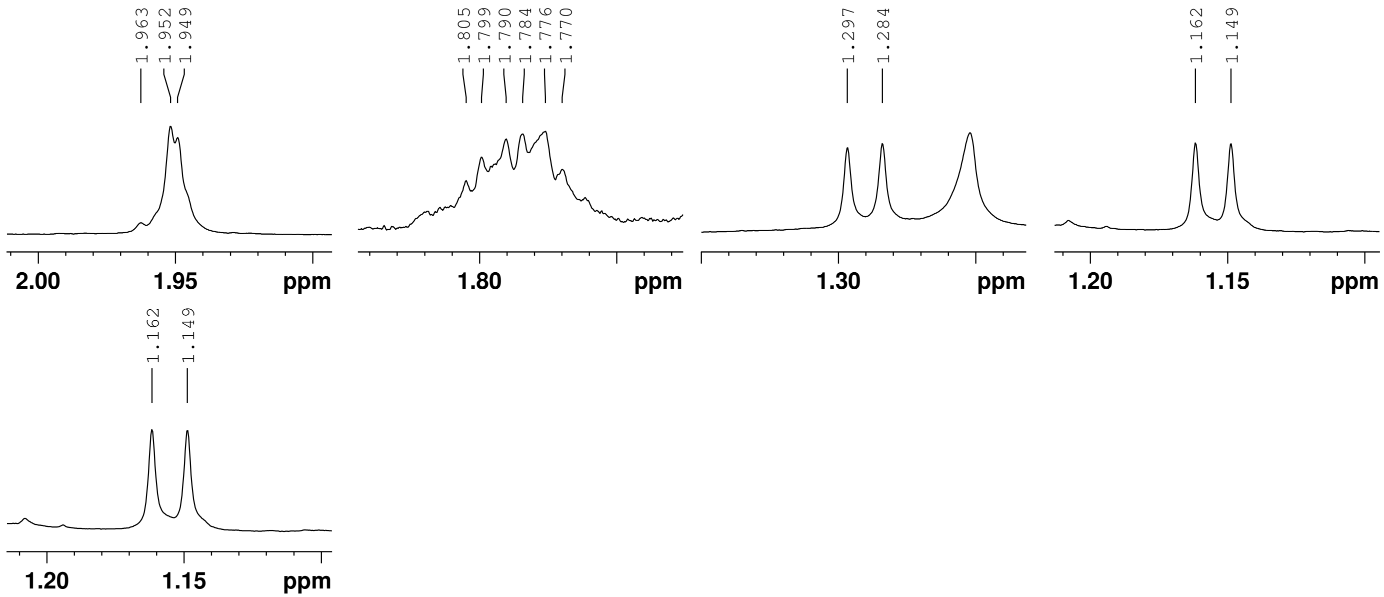

^{1}H NMR expansion of (*S*,*Z*)-*N*-((2*R*,3*R*,5*S*,6*S*)-6-allyl-2,5-dimethyltetrahydro-2*H*-pyran-3-yl)-4-(methoxymethoxy)-2-methylpent-2-enamide **4**
(500 MHz, $CDCl_3$, 296K)

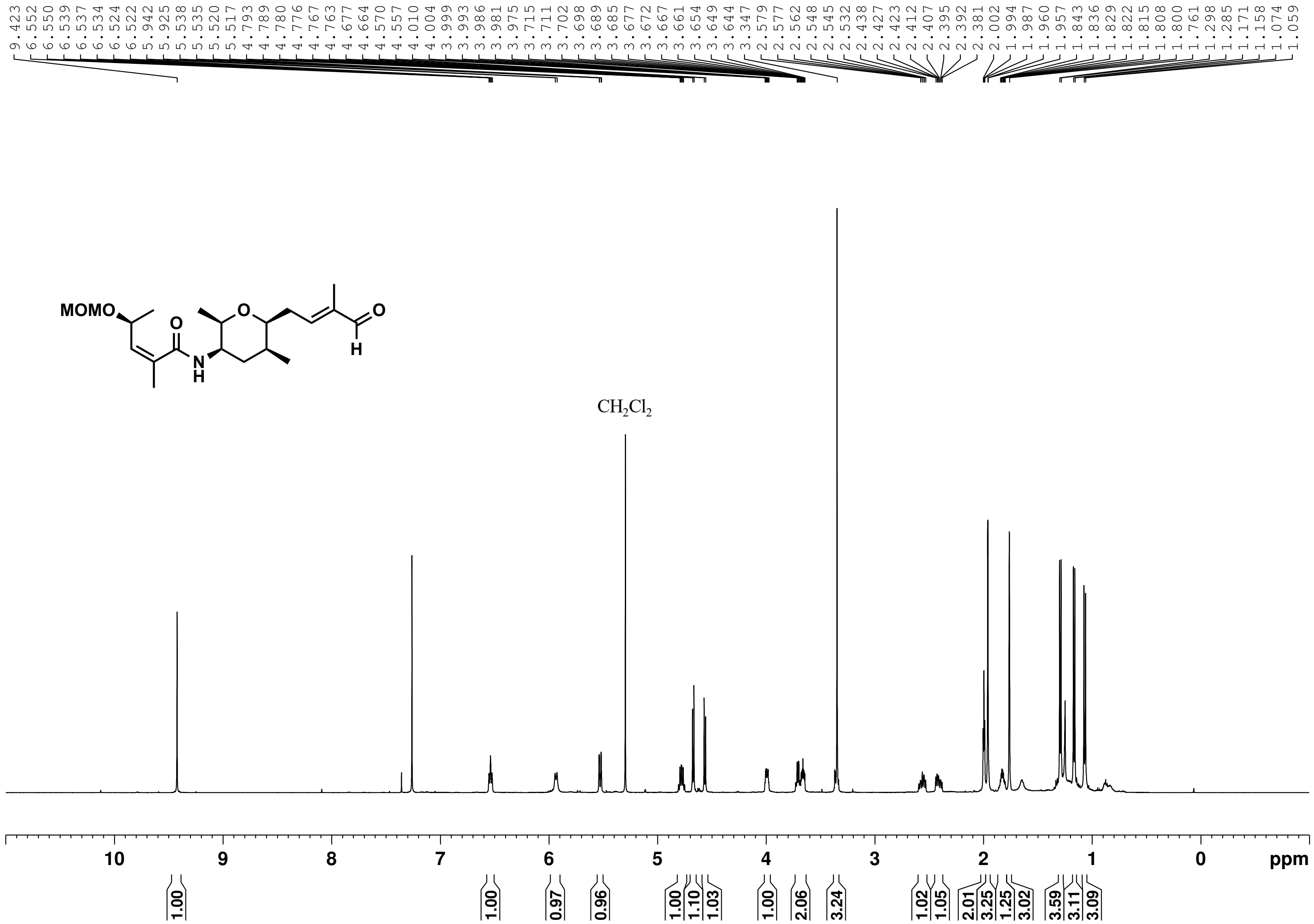

^{1}H NMR spectrum of (*S*,*Z*)-*N*-((2*R*,3*R*,5*S*,6*S*)-2,5-dimethyl-6-((*E*)-3-methyl-4-oxobut-2-en-1-yl)tetrahydro-2*H*-pyran-3-yl)-4-(methoxymethoxy)-2-methylpent-2-enamide **5** (500 MHz, $CDCl_3$, 296K)

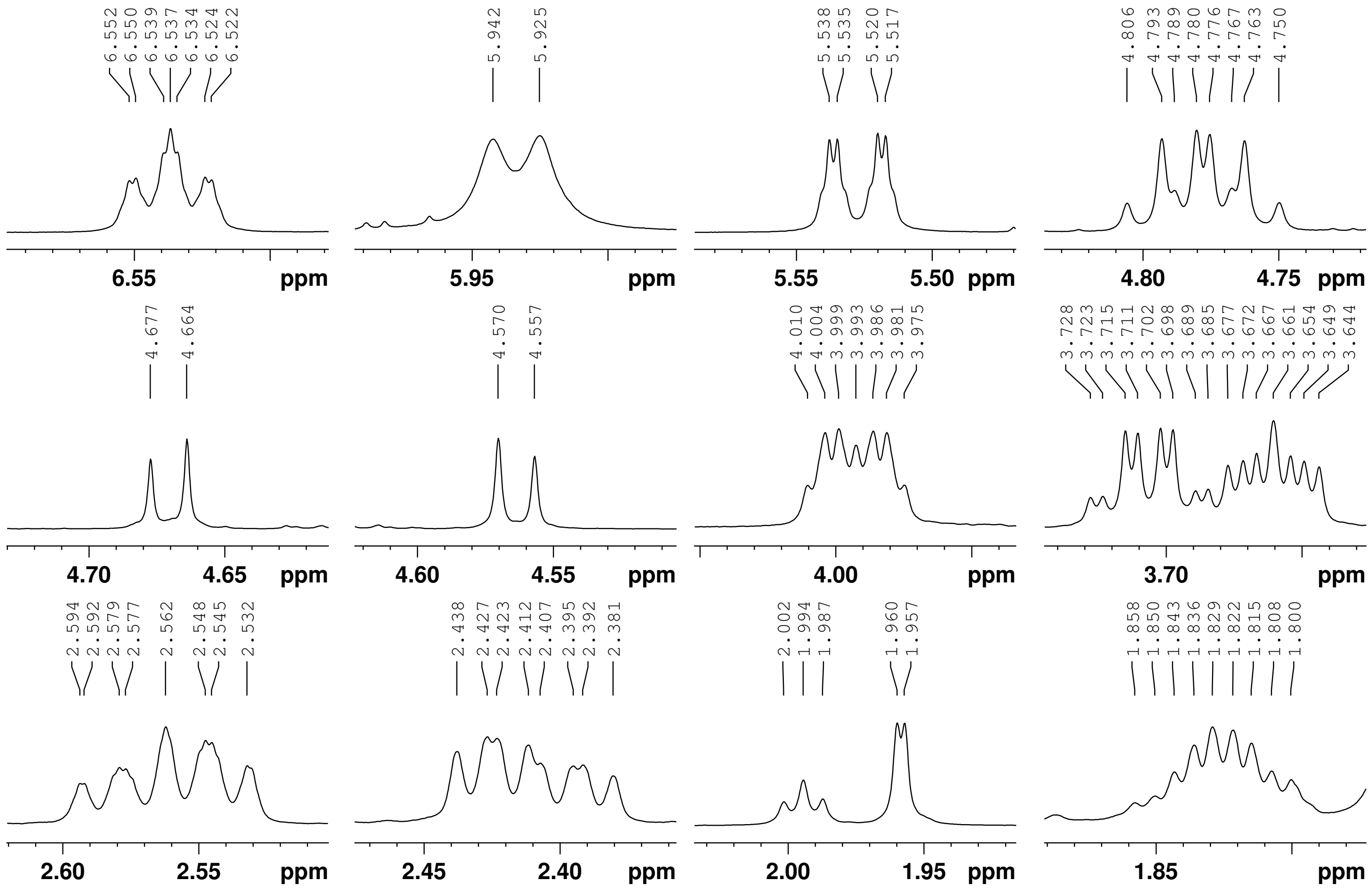

^{1}H NMR expansion of (*S*,*Z*)-*N*-((2*R*,3*R*,5*S*,6*S*)-2,5-dimethyl-6-((*E*)-3-methyl-4-oxobut-2-en-1-yl)tetrahydro-2*H*-pyran-3-yl)-4-(methoxymethoxy)-2-methylpent-2-enamide **5** (500 MHz, $CDCl_3$, 296K)

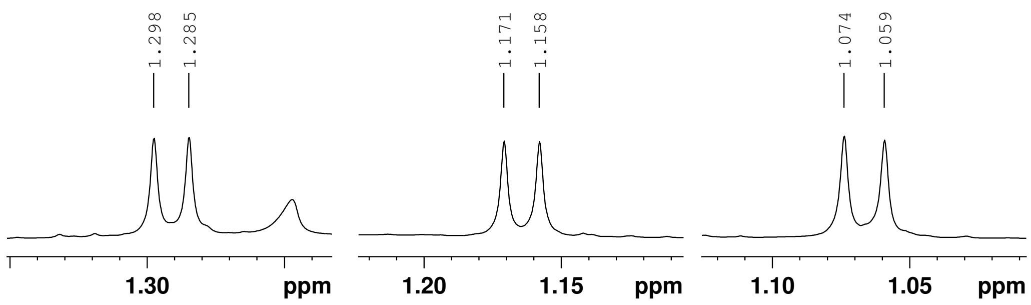

^{1}H NMR expansion of (*S*,*Z*)-*N*-((2*R*,3*R*,5*S*,6*S*)-2,5-dimethyl-6-((*E*)-3-methyl-4-oxobut-2-en-1-yl)tetrahydro-2*H*-pyran-3-yl)-4-(methoxymethoxy)-2-methylpent-2-enamide **5** (500 MHz, $CDCl_3$, 296K)

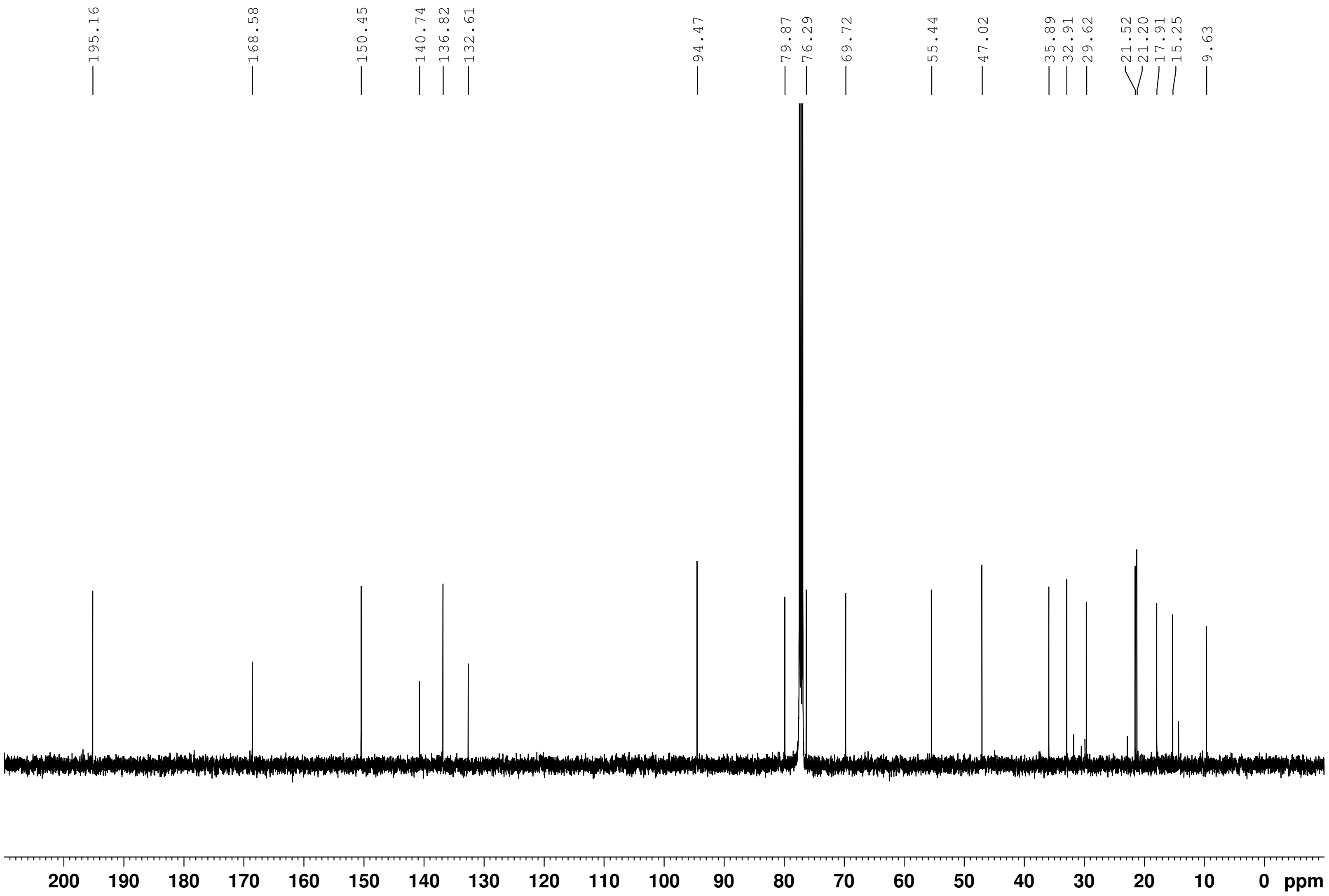

$^{13}C\{^1H\}$ NMR spectrum of (*S*,*Z*)-*N*-((2*R*,3*R*,5*S*,6*S*)-2,5-dimethyl-6-((*E*)-3-methyl-4-oxobut-2-en-1-yl)tetrahydro-2*H*-pyran-3-yl)-4-(methoxymethoxy)-2-methylpent-2-enamide **5** (125 MHz, $CDCl_3$, 296K)

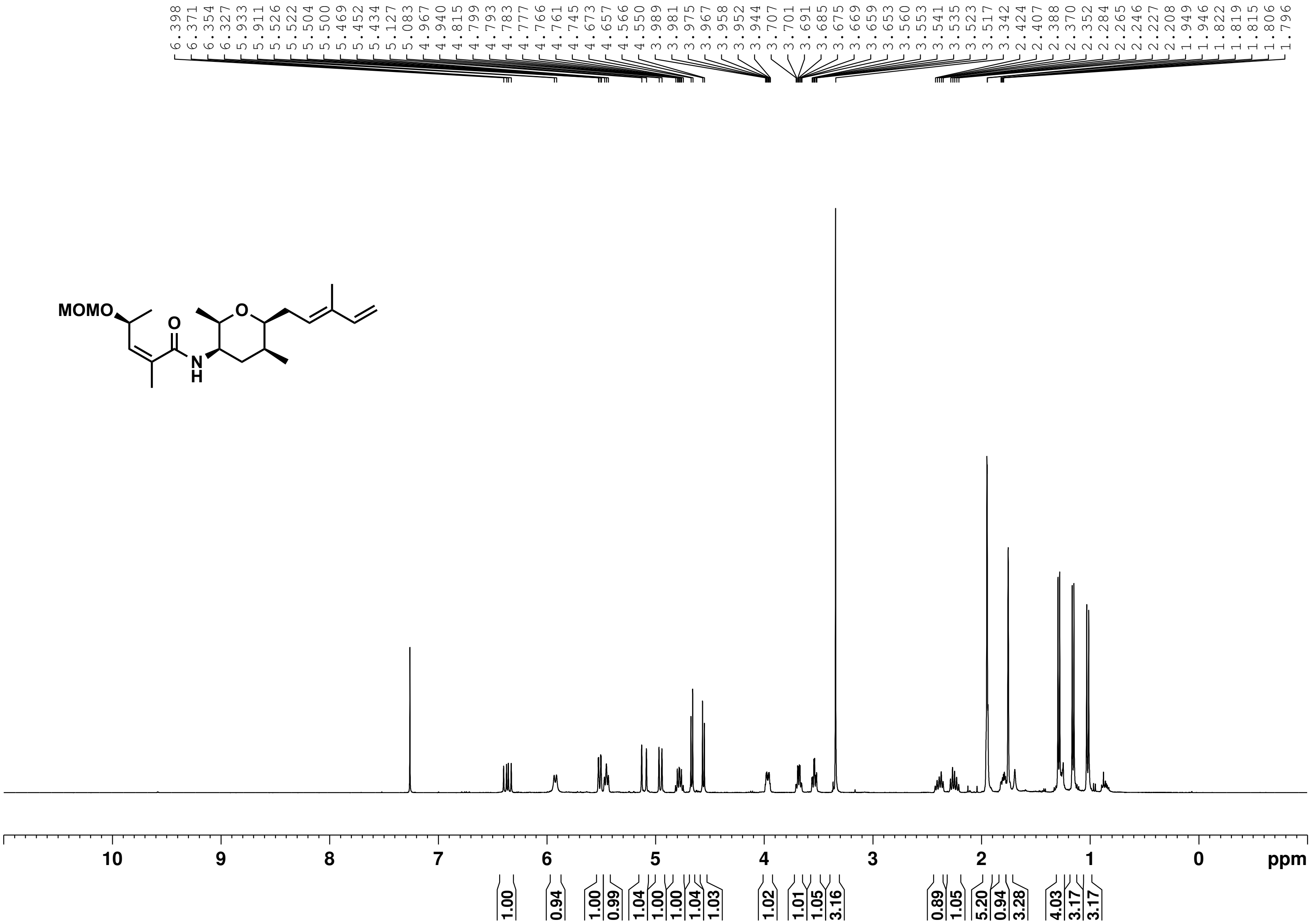

^{1}H NMR spectrum of (*S*,*Z*)-*N*-((2*R*,3*R*,5*S*,6*S*)-2,5-dimethyl-6-((*E*)-3-methylpenta-2,4-dien-1-yl)tetrahydro-2*H*-pyran-3-yl)-4-(methoxymethoxy)-2-methylpent-2-enamide **6** (400 MHz, $CDCl_3$, 296K)

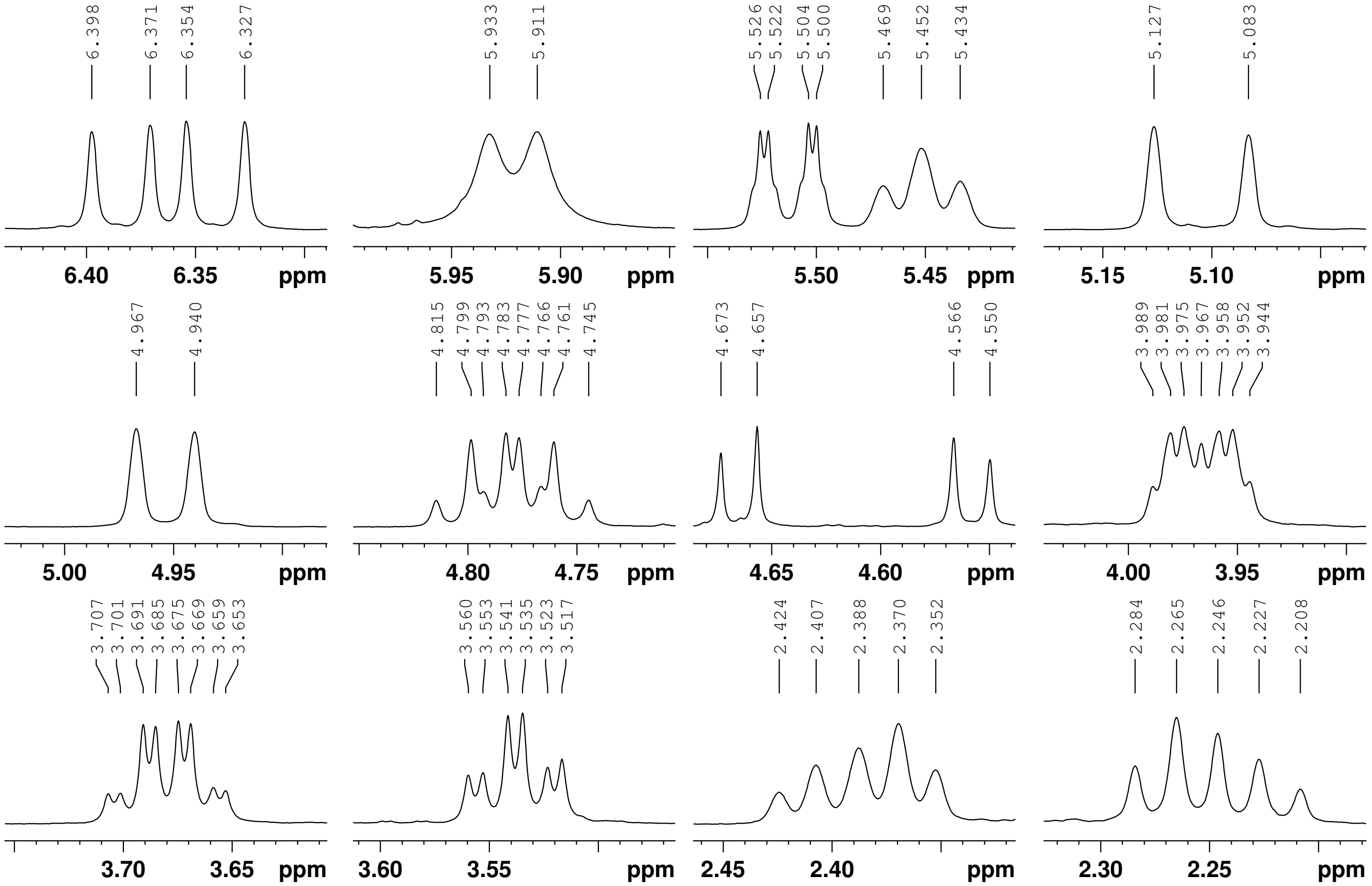

^{1}H NMR expansion of (*S*,*Z*)-*N*-((2*R*,3*R*,5*S*,6*S*)-2,5-dimethyl-6-((*E*)-3-methylpenta-2,4-dien-1-yl)tetrahydro-2*H*-pyran-3-yl)-4-(methoxymethoxy)-2-methylpent-2-enamide **6** (400 MHz, $CDCl_3$, 296K)

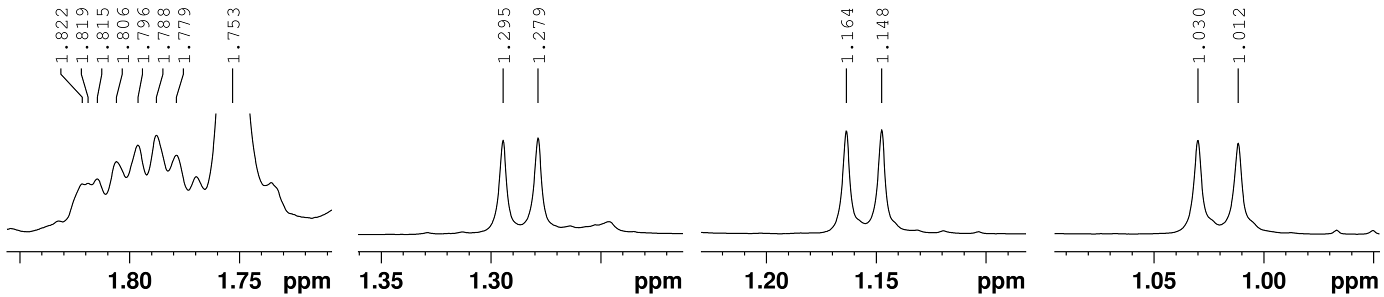

^{1}H NMR expansion of (*S*,*Z*)-*N*-((2*R*,3*R*,5*S*,6*S*)-2,5-dimethyl-6-((*E*)-3-methylpenta-2,4-dien-1-yl)tetrahydro-2*H*-pyran-3-yl)-4-(methoxymethoxy)-2-methylpent-2-enamide **6** (400 MHz, $CDCl_3$, 296K)

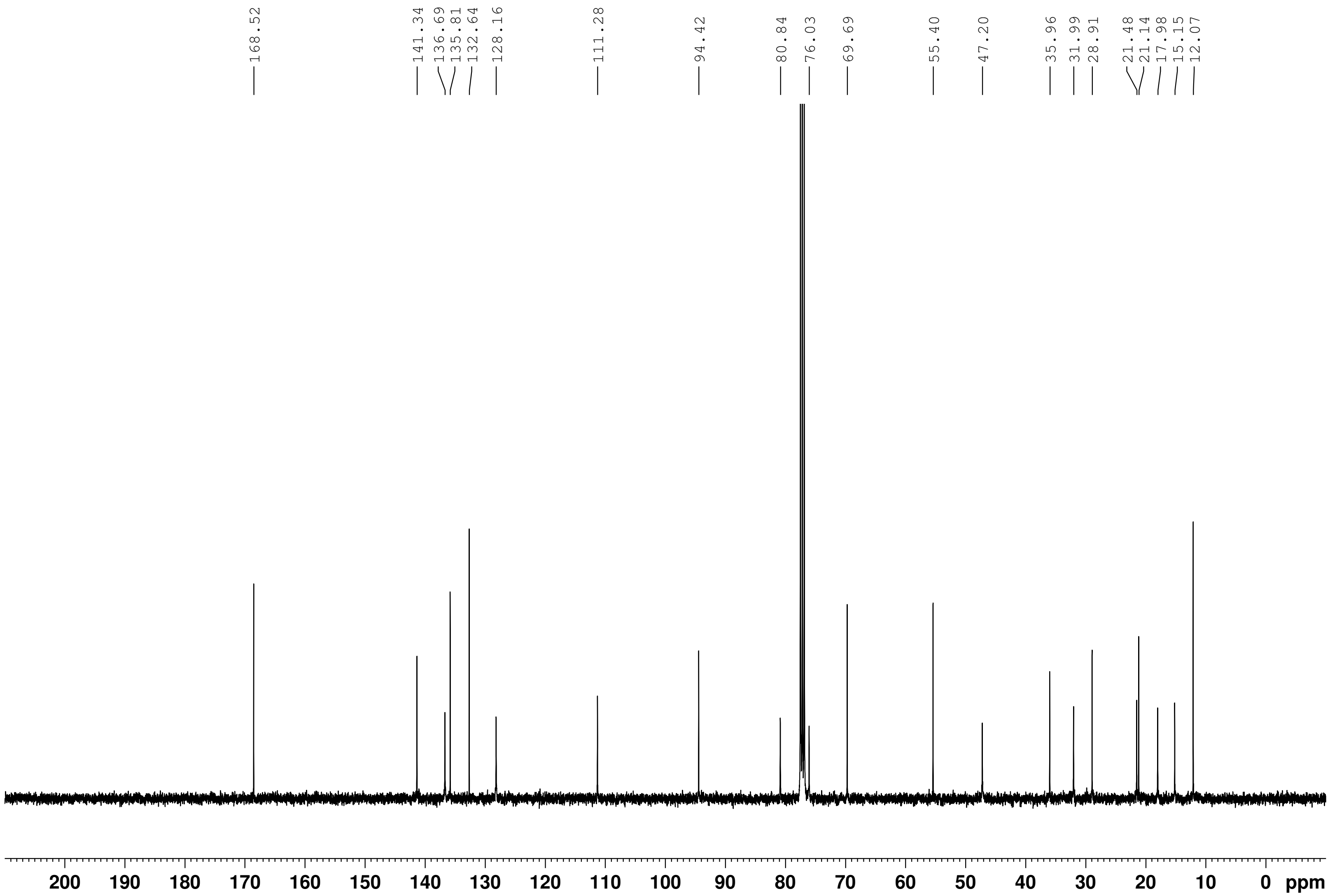

$^{13}C\{^1H\}$ NMR spectrum of (*S*,*Z*)-*N*-((2*R*,3*R*,5*S*,6*S*)-2,5-dimethyl-6-((*E*)-3-methylpenta-2,4-dien-1-yl)tetrahydro-2*H*-pyran-3-yl)-4-(methoxymethoxy)-2-methylpent-2-enamide **6** (100 MHz, $CDCl_3$, 296K)

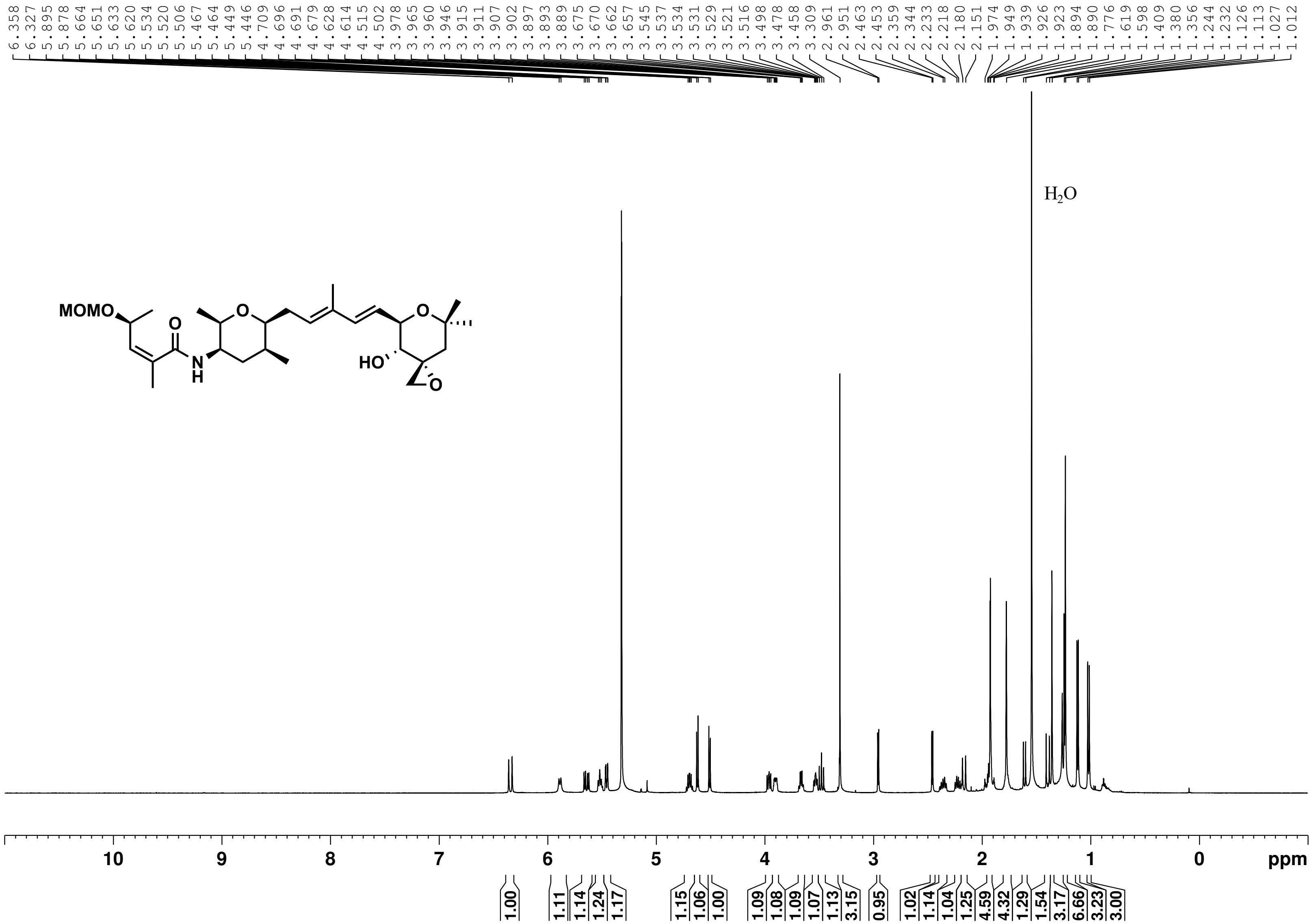

^1H NMR spectrum of 2'-Me meayamycin D (500 MHz, CD_2Cl_2, 296K)

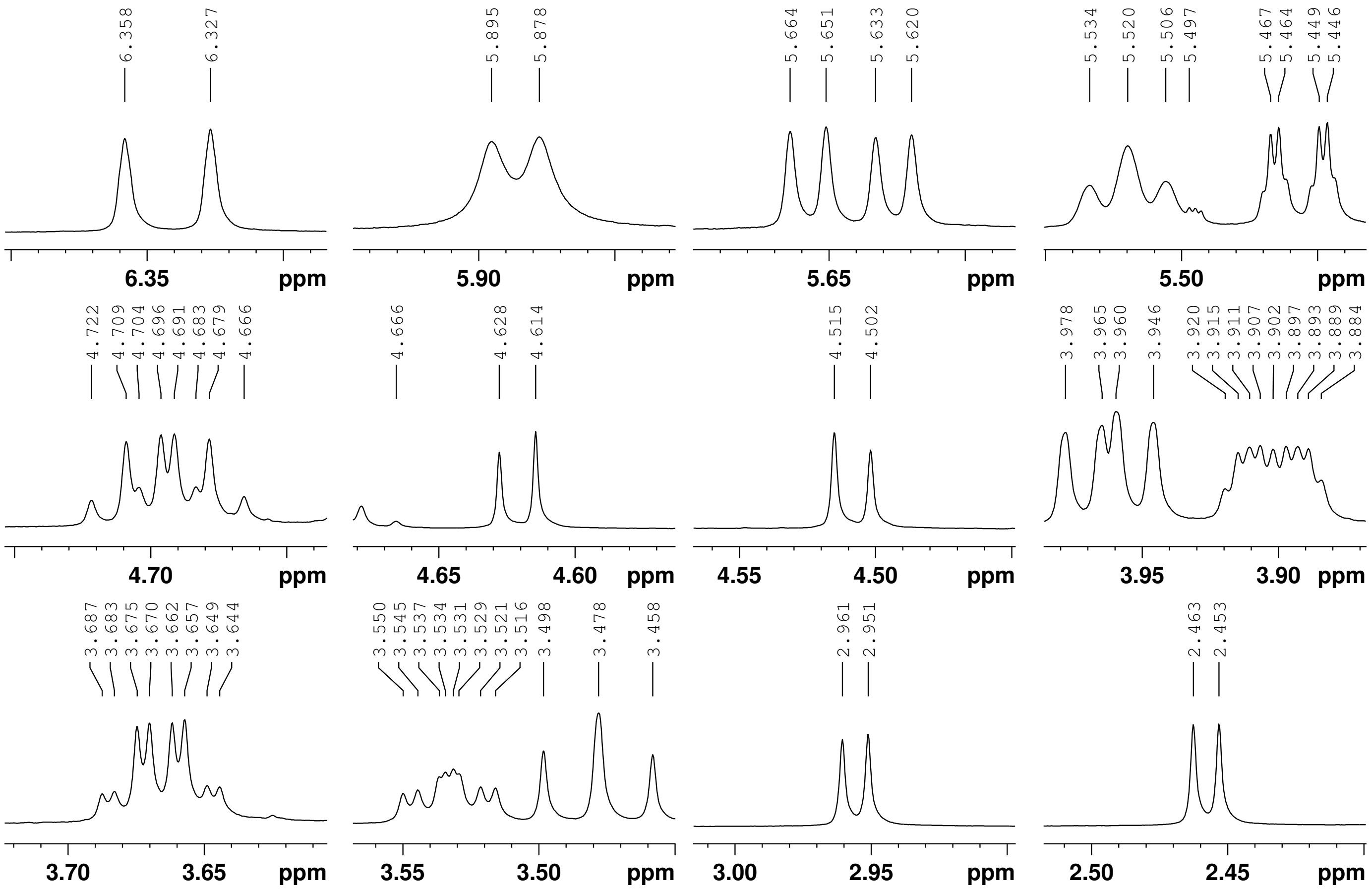

^{1}H NMR expansion of 2'-Me meayamycin D (500 MHz, CD_2Cl_2, 296K)

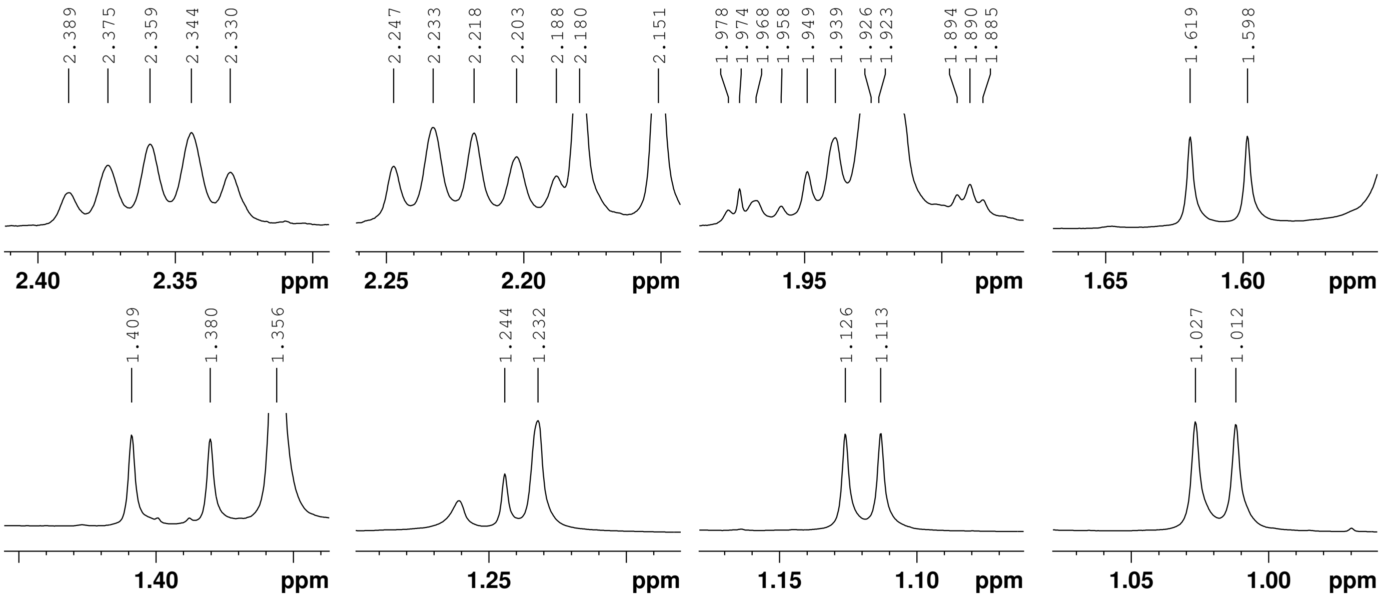

^{1}H NMR expansion of 2'-Me meayamycin D (500 MHz, CD_2Cl_2, 296K)

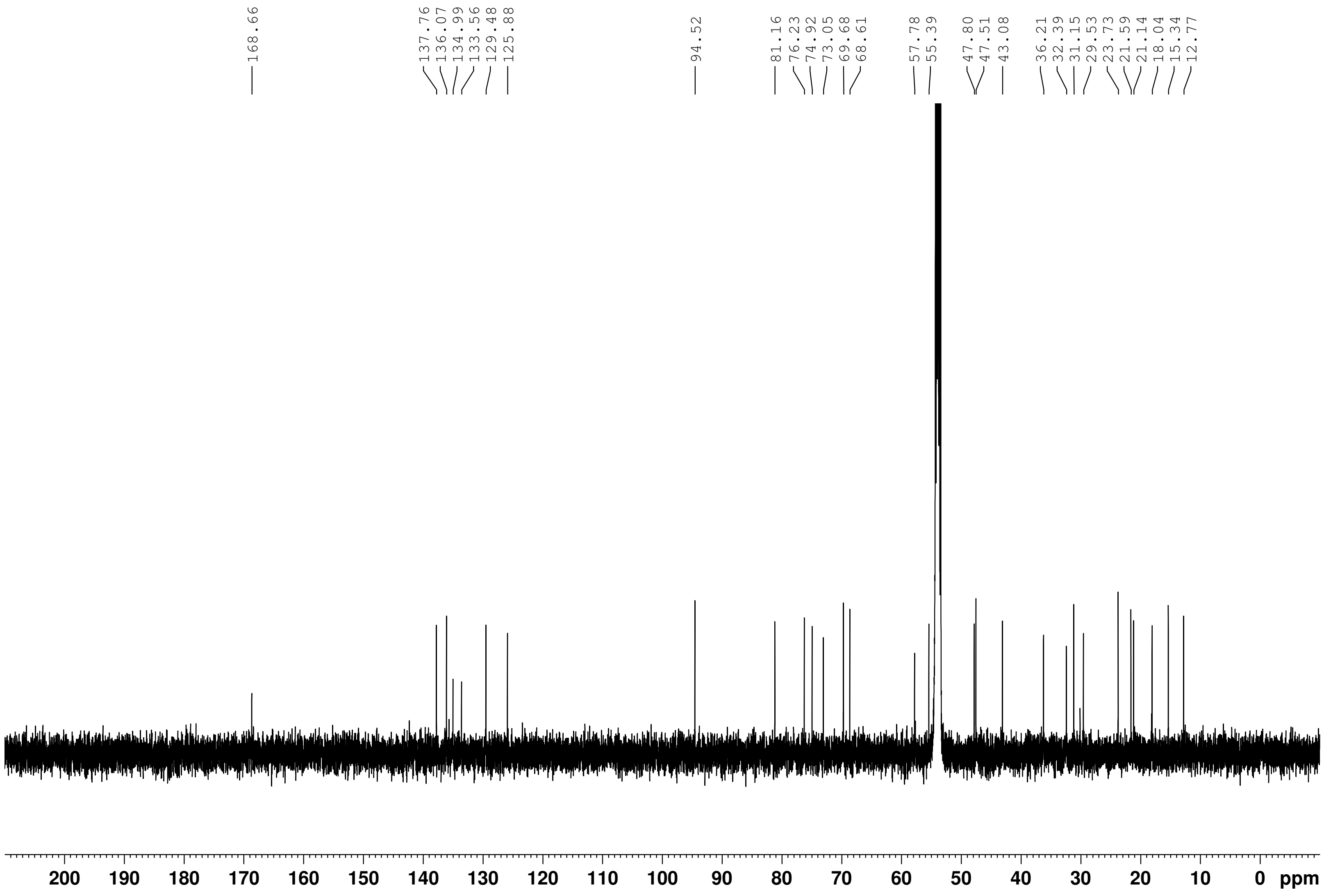

$^{13}C\{^{1}H\}$ NMR spectrum of 2'-Me meayamycin D (500 MHz, CD_2Cl_2, 296K)

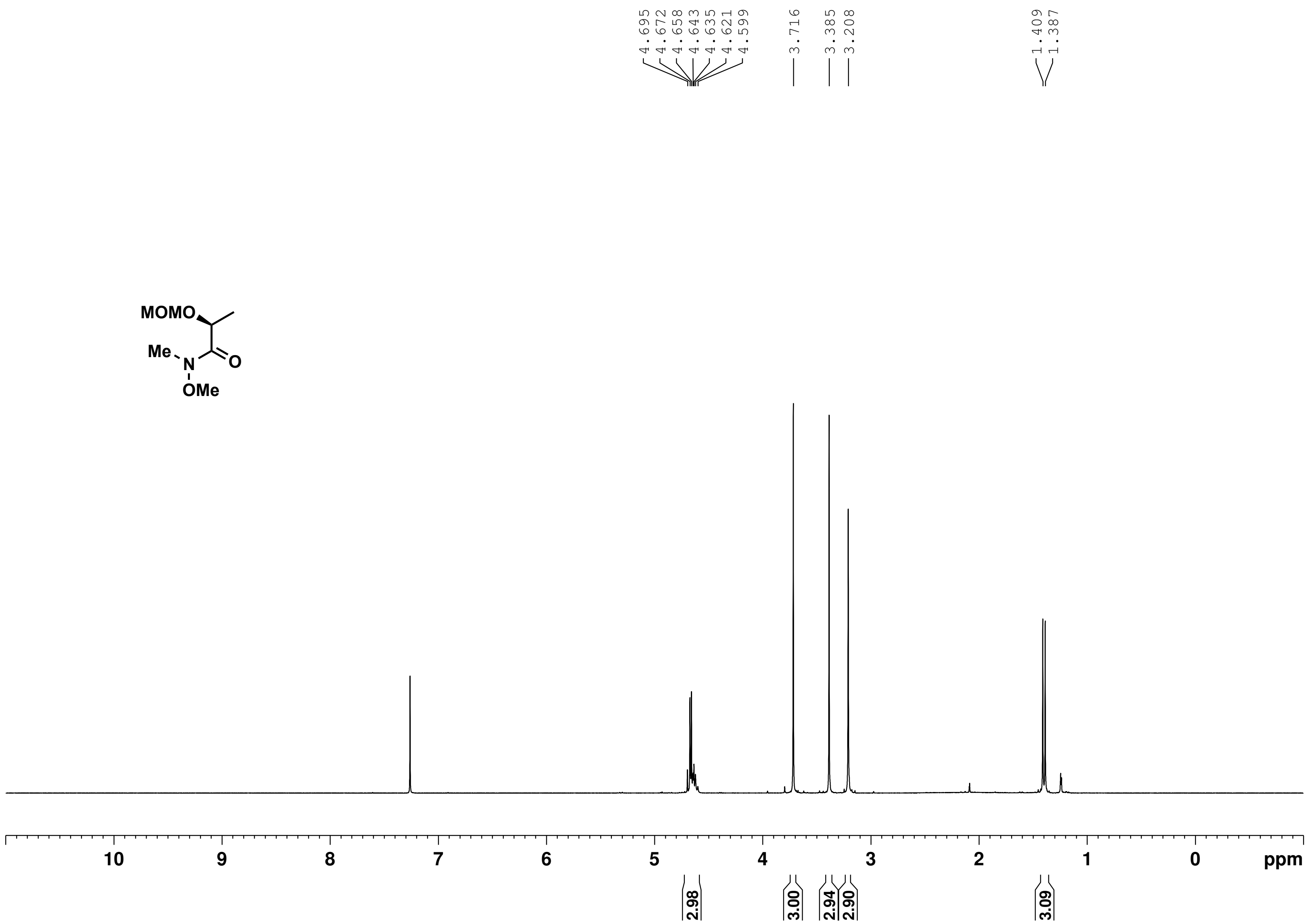

^{1}H NMR spectrum of (*S*)-*N*-methoxy-2-(methoxymethoxy)-*N*-methylpropanamide **12** (300 MHz, $CDCl_3$, 296K)

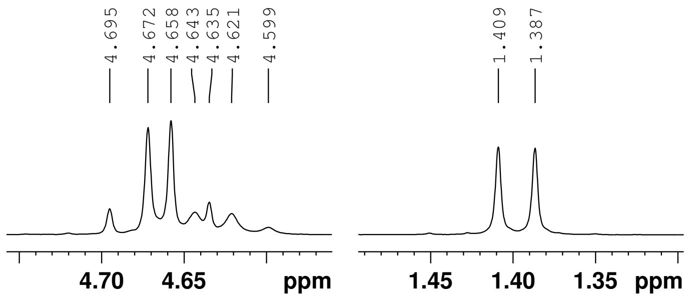

^{1}H NMR expansion of (*S*)-*N*-methoxy-2-(methoxymethoxy)-*N*-methylpropanamide **12** (300 MHz, $CDCl_3$, 296K)

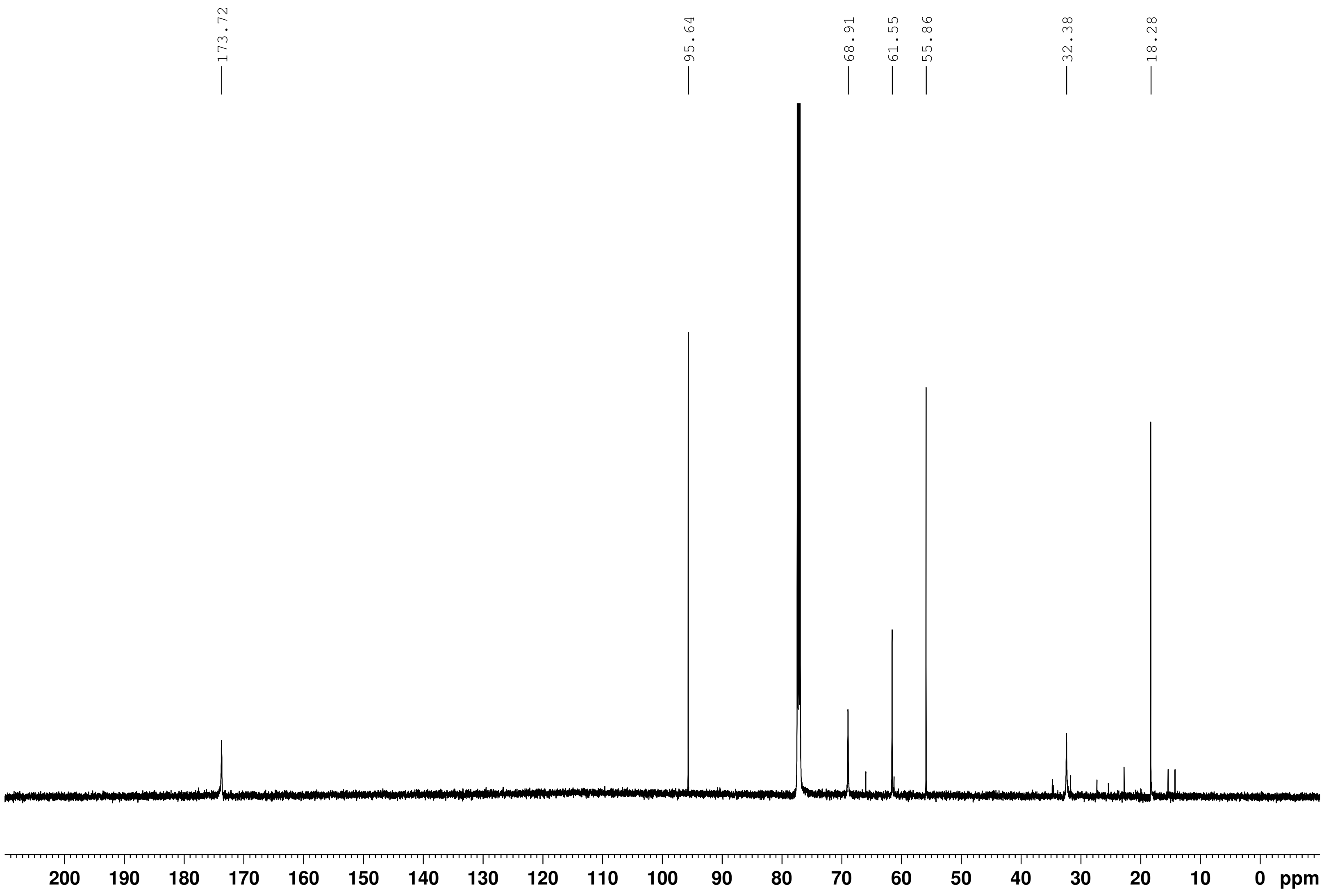

$^{13}C\{^{1}H\}$ NMR spectrum of (*S*)-*N*-methoxy-2-(methoxymethoxy)-*N*-methylpropanamide **12** (150 MHz, $CDCl_3$, 296K)

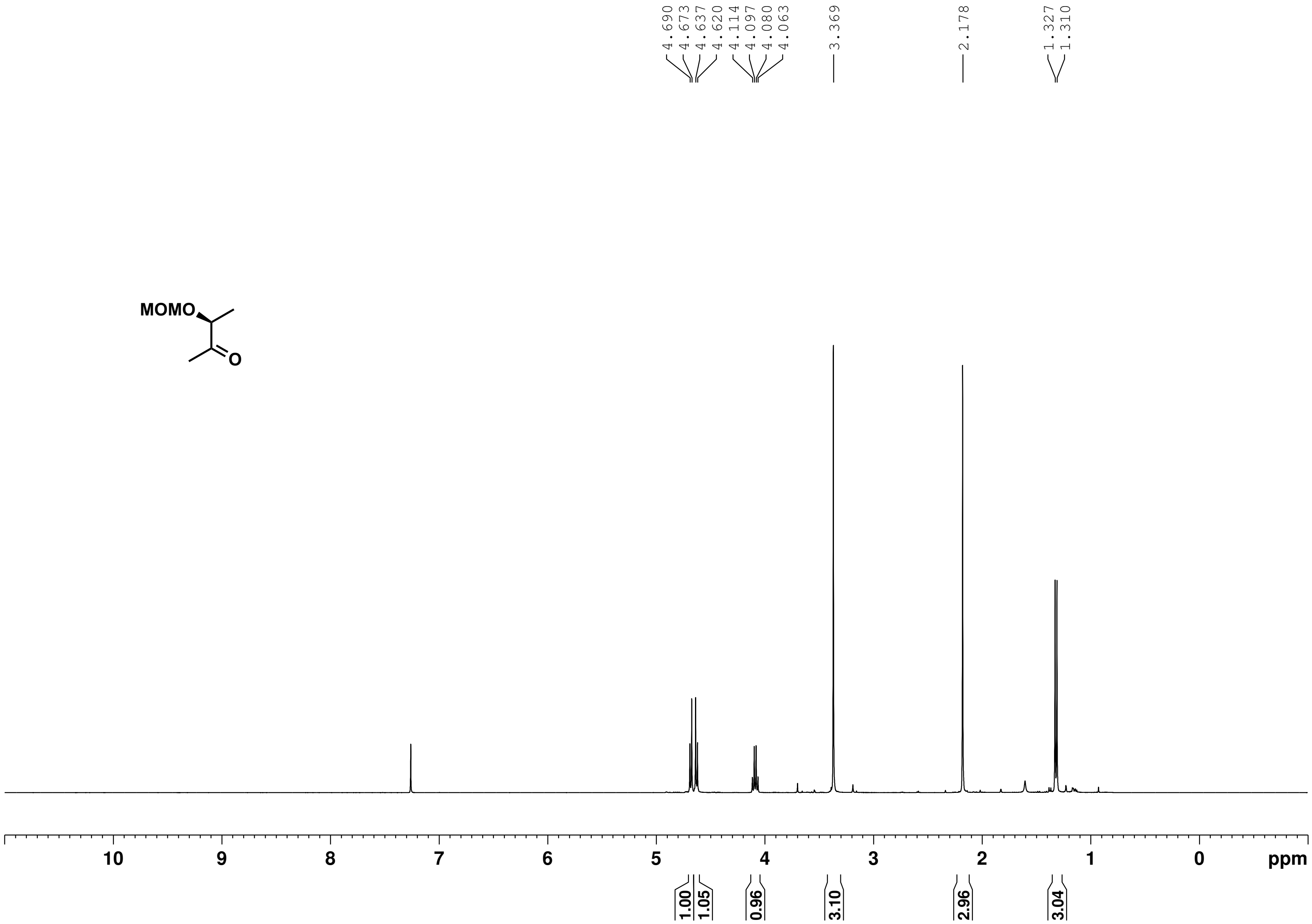

^{1}H NMR spectrum of (*S*)-3-(methoxymethoxy)butan-2-one **13** (400 MHz, 1% CD_3OD in $CDCl_3$, 296K)

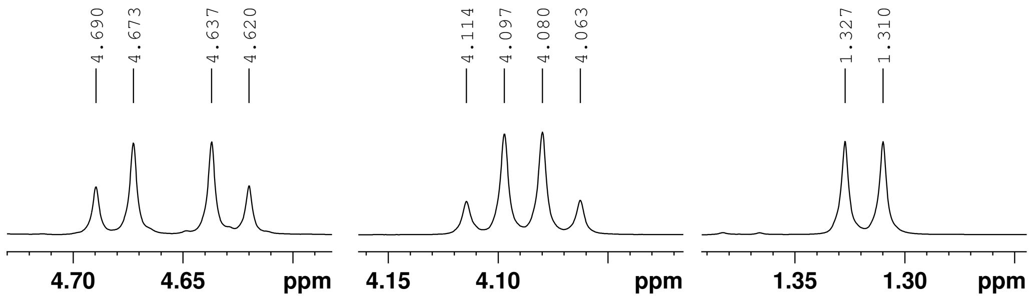

^{1}H NMR expansion of (*S*)-3-(methoxymethoxy)butan-2-one **13** (400 MHz, 1% CD_3OD in $CDCl_3$, 296K)

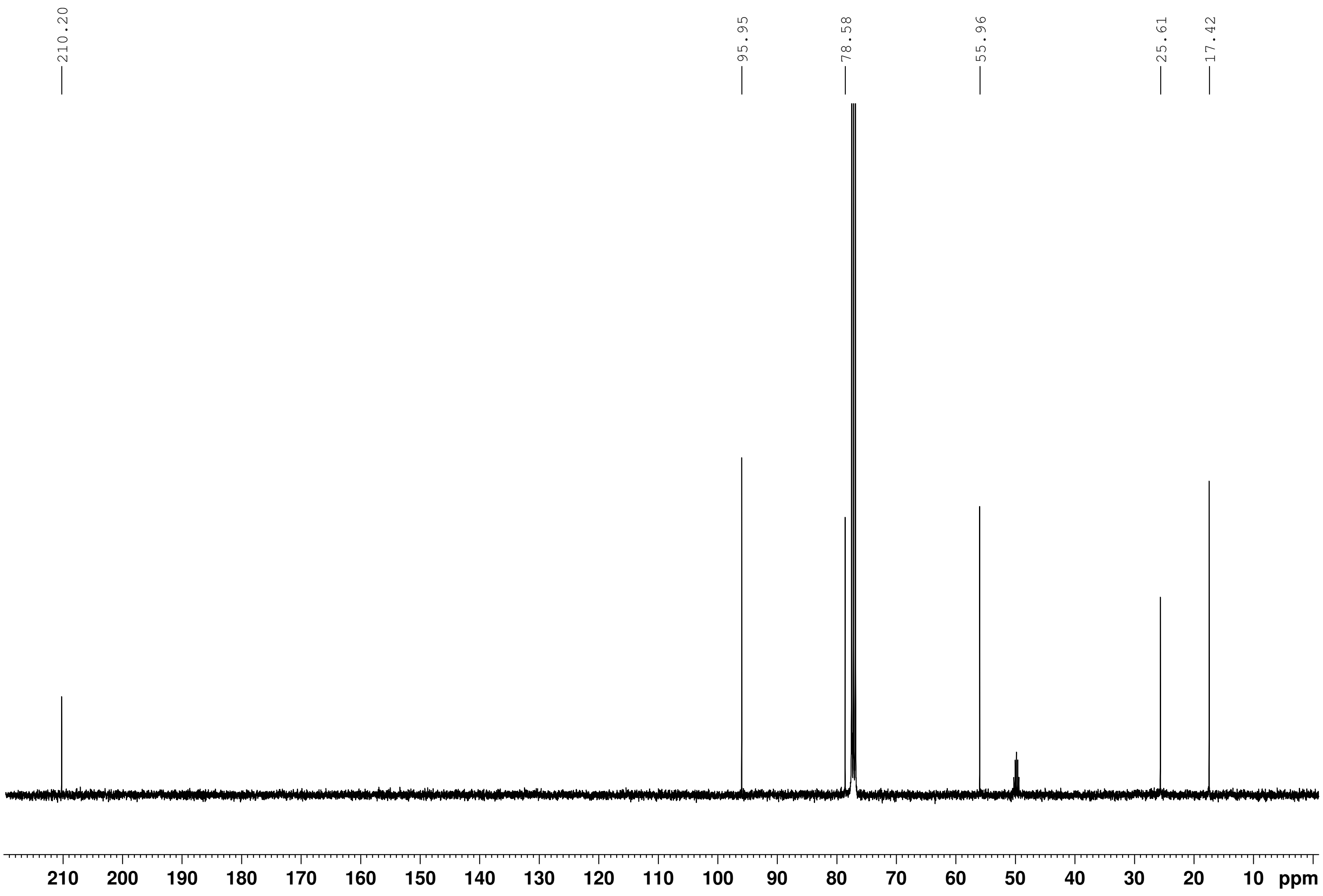

$^{13}C\{^1H\}$ NMR spectrum of (*S*)-3-(methoxymethoxy)butan-2-one **13** (100 MHz, 1% CD_3OD in $CDCl_3$, 296K)

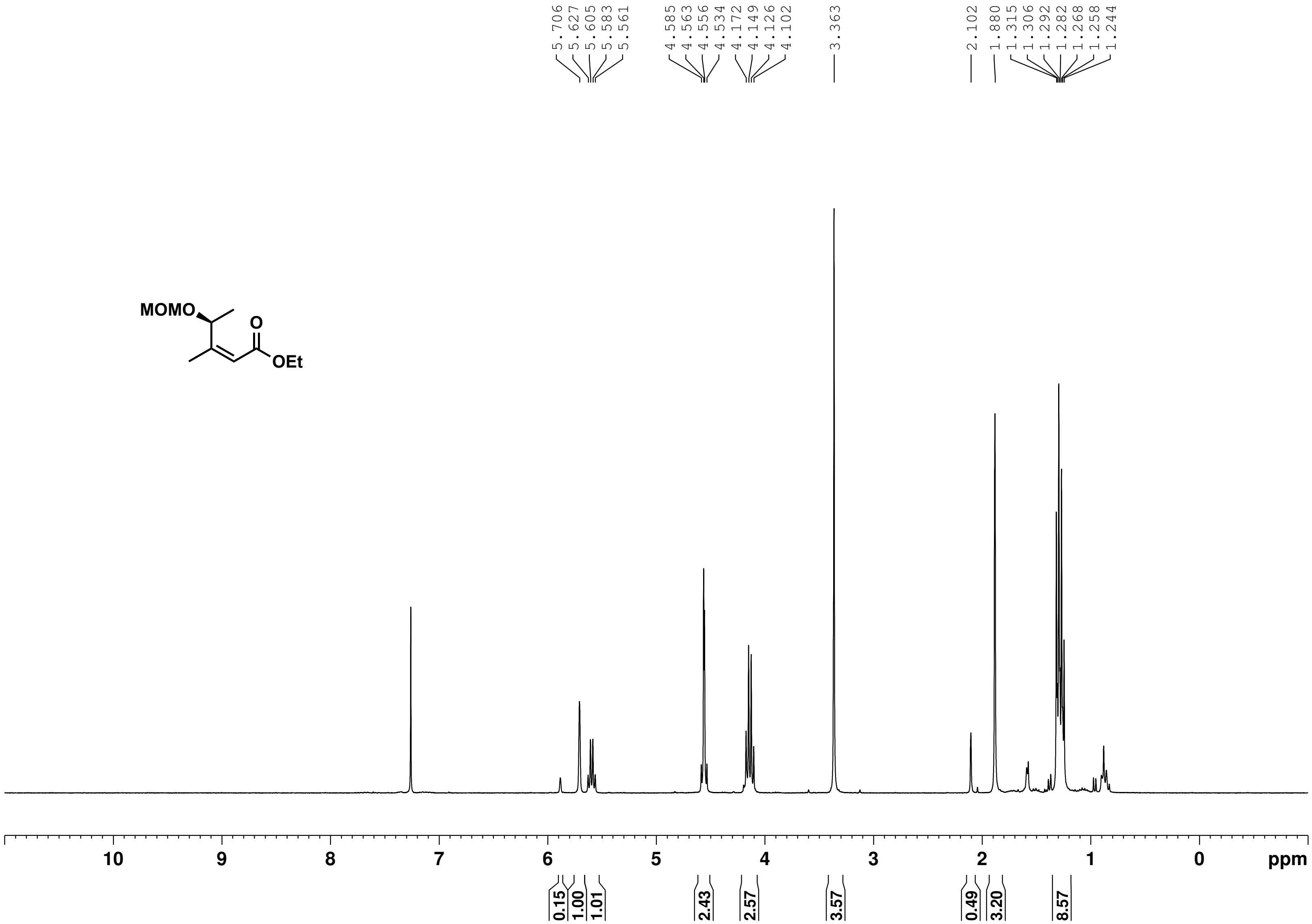

^{1}H NMR spectrum of (*S*,*Z*)-4-(methoxymethoxy)-3-methylpent-2-enoate **14** (300 MHz, $CDCl_3$, 296K)

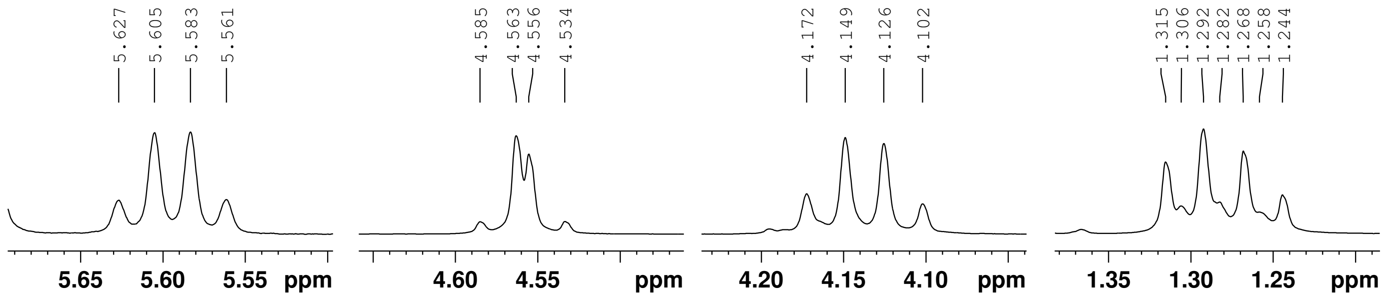

^{1}H NMR expansion of (*S*,*Z*)-4-(methoxymethoxy)-3-methylpent-2-enoate **14** (300 MHz, $CDCl_3$, 296K)

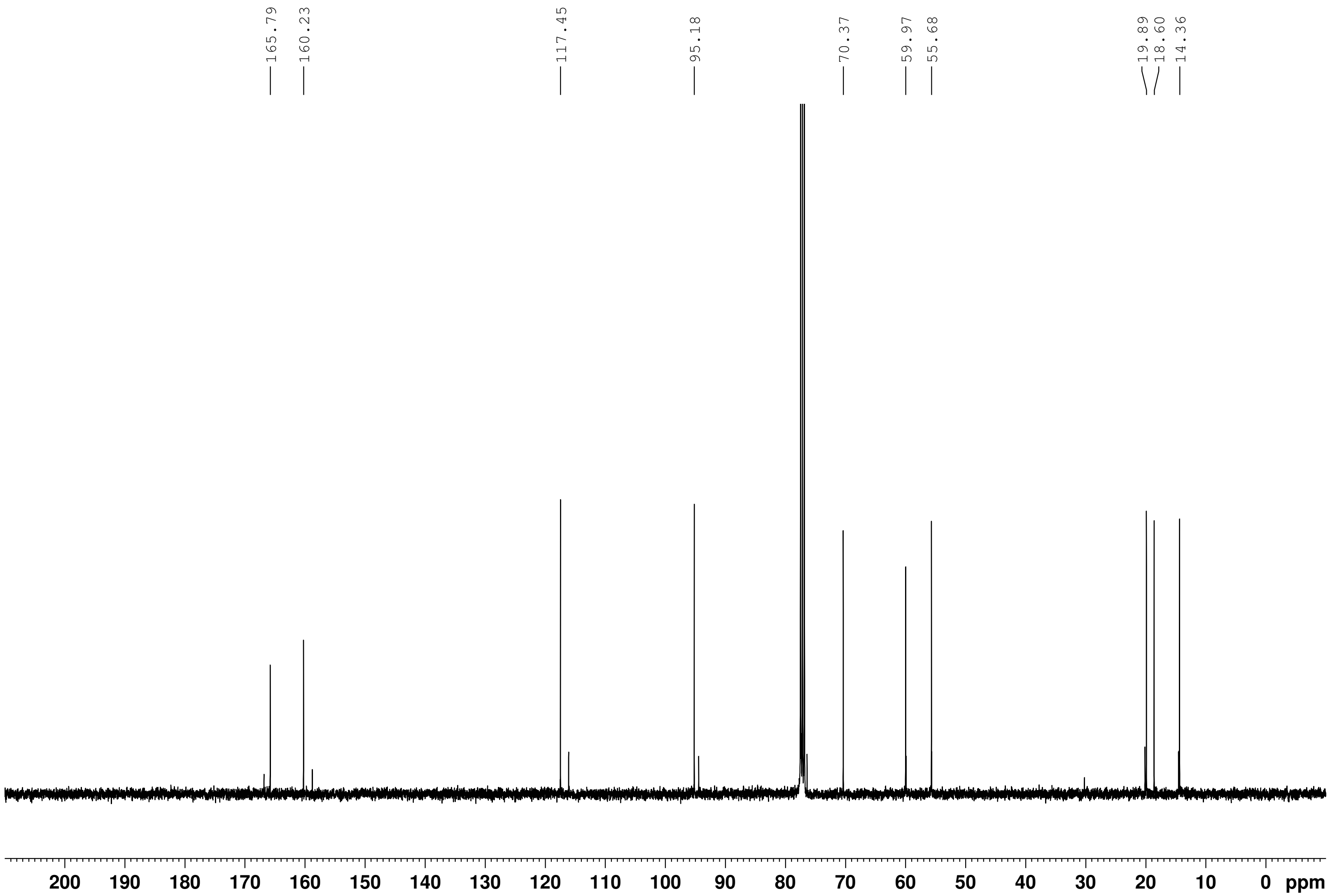

$^{13}C\{^{1}H\}$ NMR spectrum of (*S*,*Z*)-4-(methoxymethoxy)-3-methylpent-2-enoate **14** (100 MHz, $CDCl_3$, 296K)

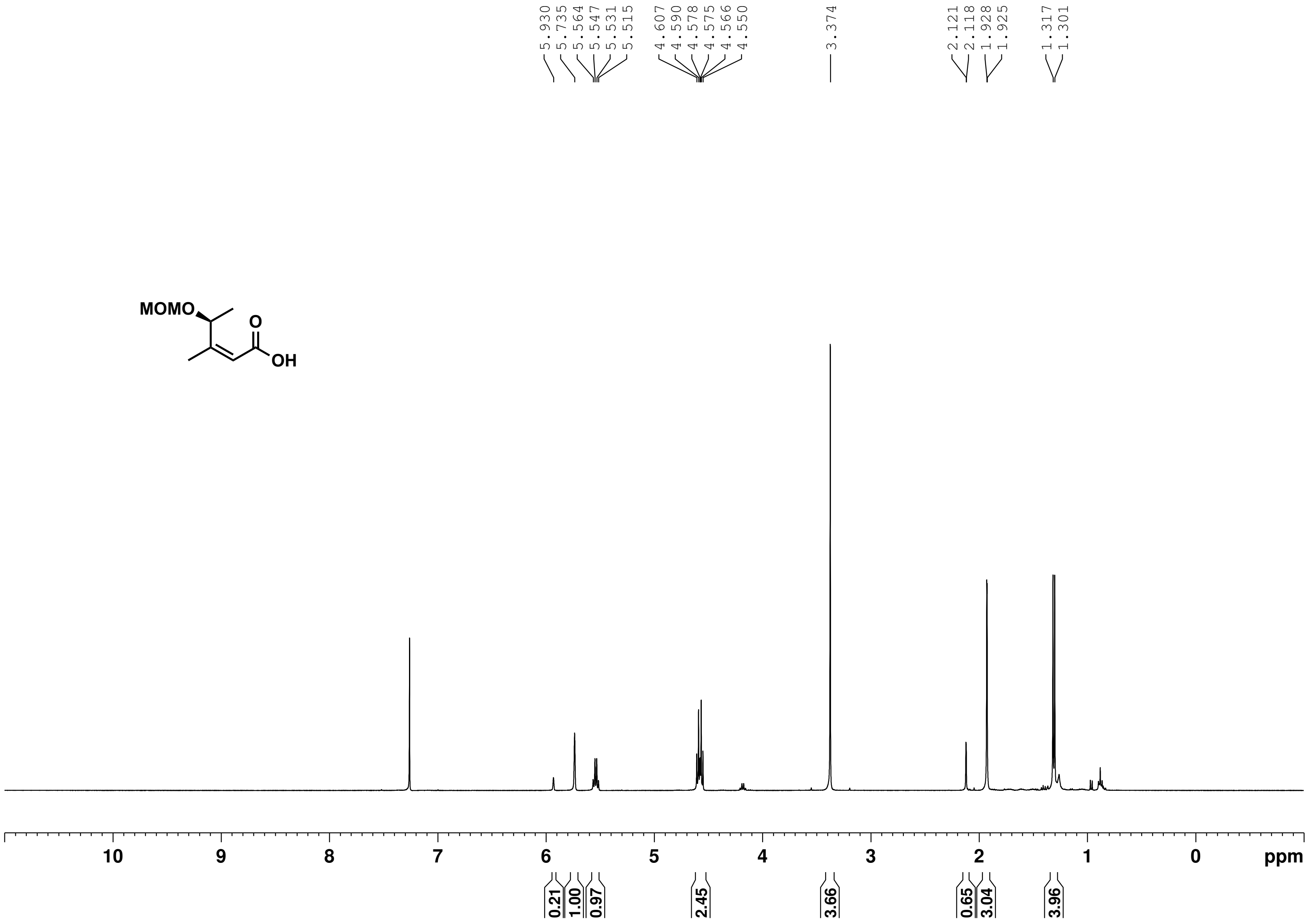

^{1}H NMR spectrum of (*S*,*Z*)-4-(methoxymethoxy)-3-methylpent-2-enoic acid **15** (400 MHz, 1% CD_3OD in $CDCl_3$, 296K)

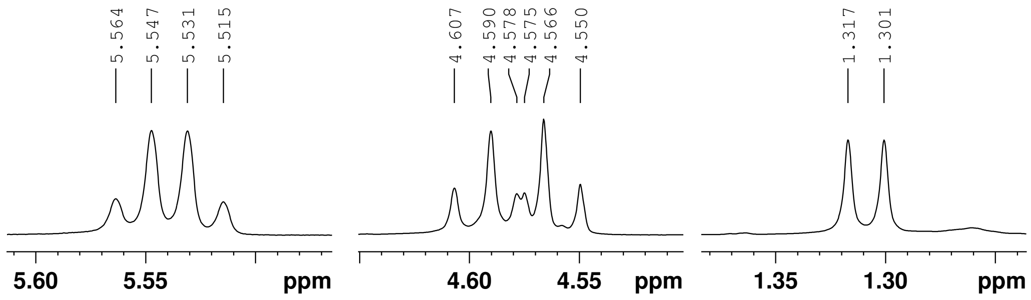

^{1}H NMR expansion of (*S*,*Z*)-4-(methoxymethoxy)-3-methylpent-2-enoic acid **15** (400 MHz, 1% CD_3OD in $CDCl_3$, 296K)

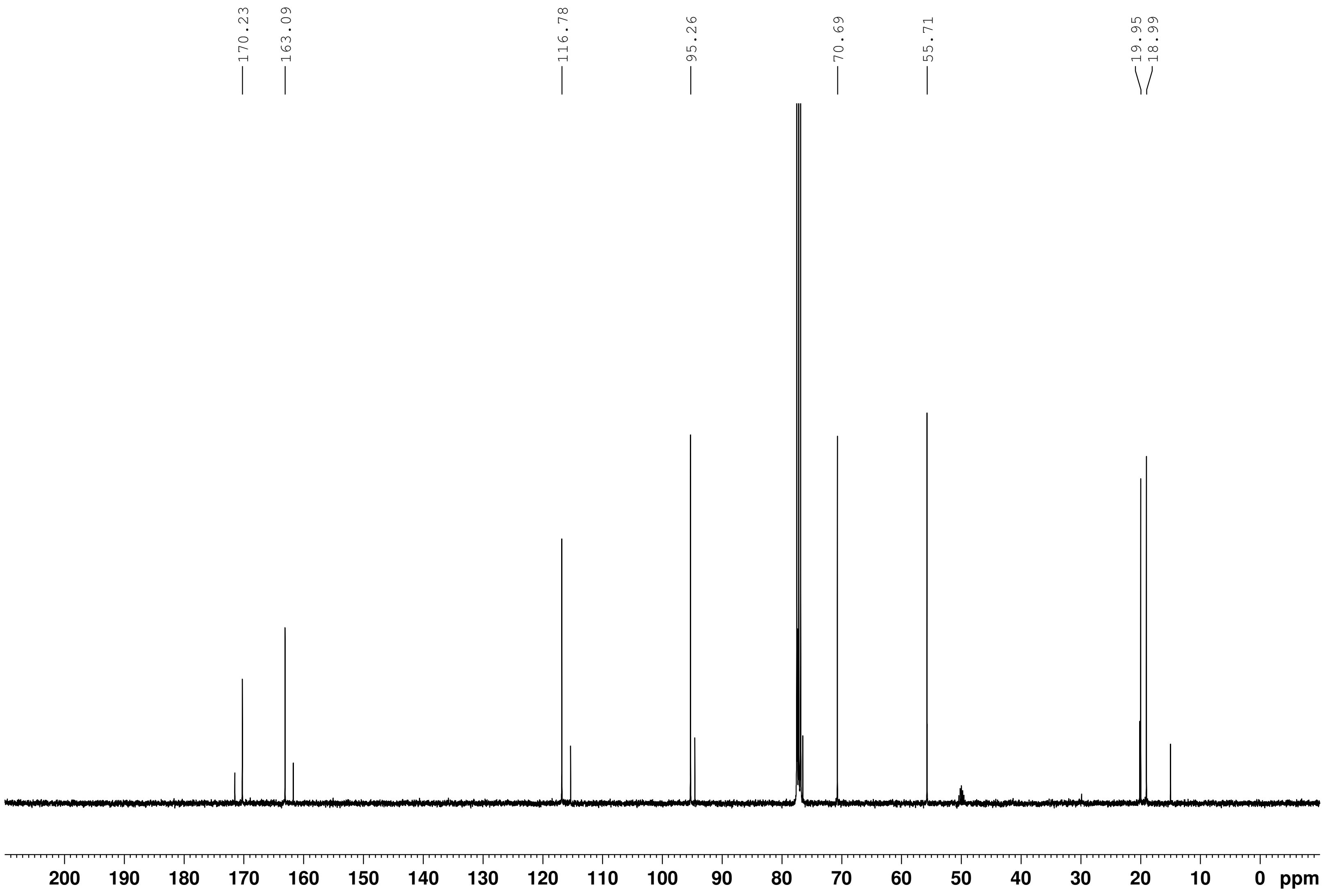

$^{13}C\{^1H\}$ NMR spectrum of (*S*,*Z*)-4-(methoxymethoxy)-3-methylpent-2-enoic acid **15** (100 MHz, 1% CD_3OD in $CDCl_3$, 296K)

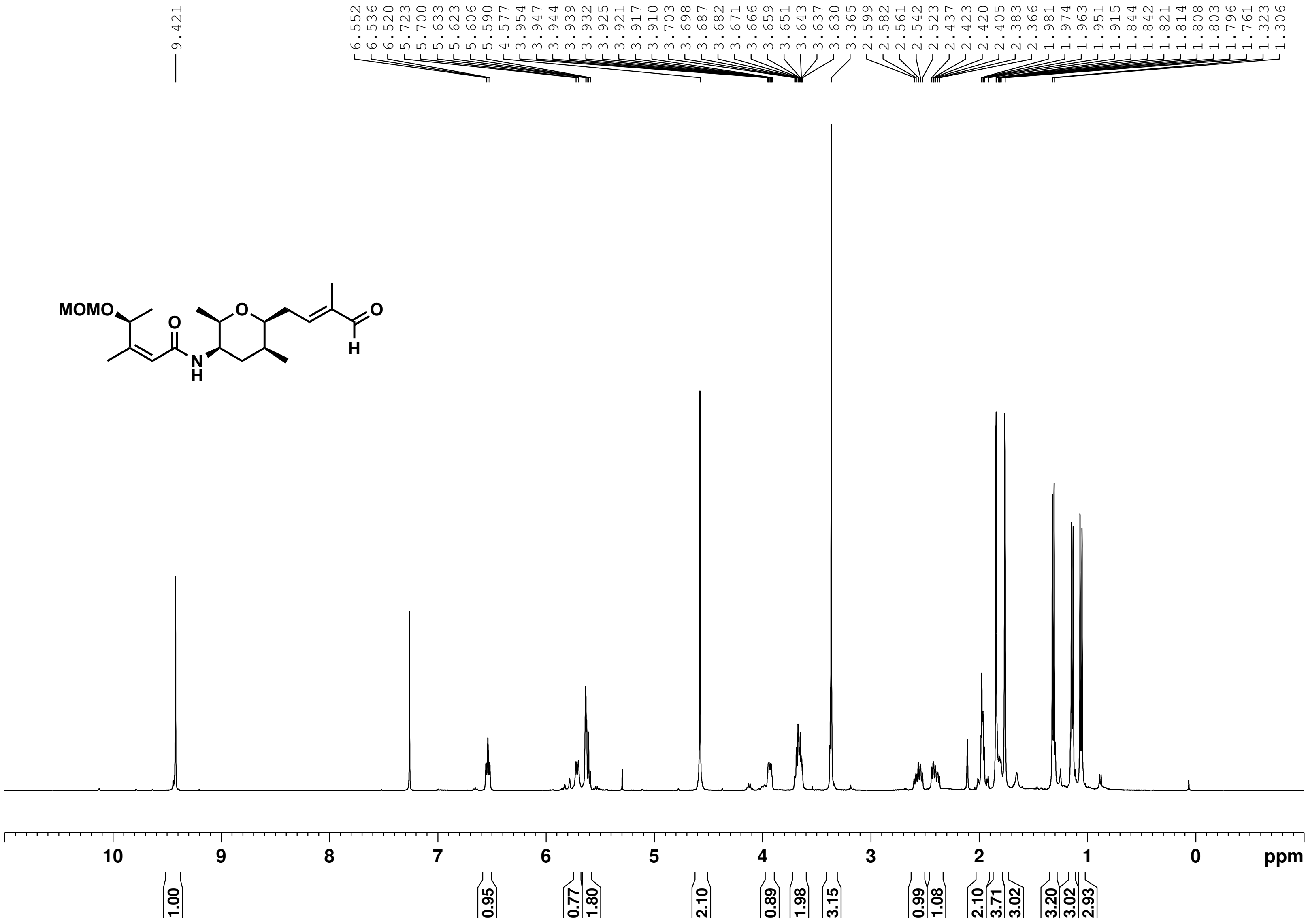

^{1}H NMR spectrum of (*S*,*Z*)-*N*-((2*R*,3*R*,5*S*,6*S*)-2,5-dimethyl-6-((*E*)-3-methyl-4-oxobut-2-en-1-yl)tetrahydro-2*H*-pyran-3-yl)-4-(methoxymethoxy)-3-methylpent-2-enamide **17** (400 MHz, $CDCl_3$, 296K)

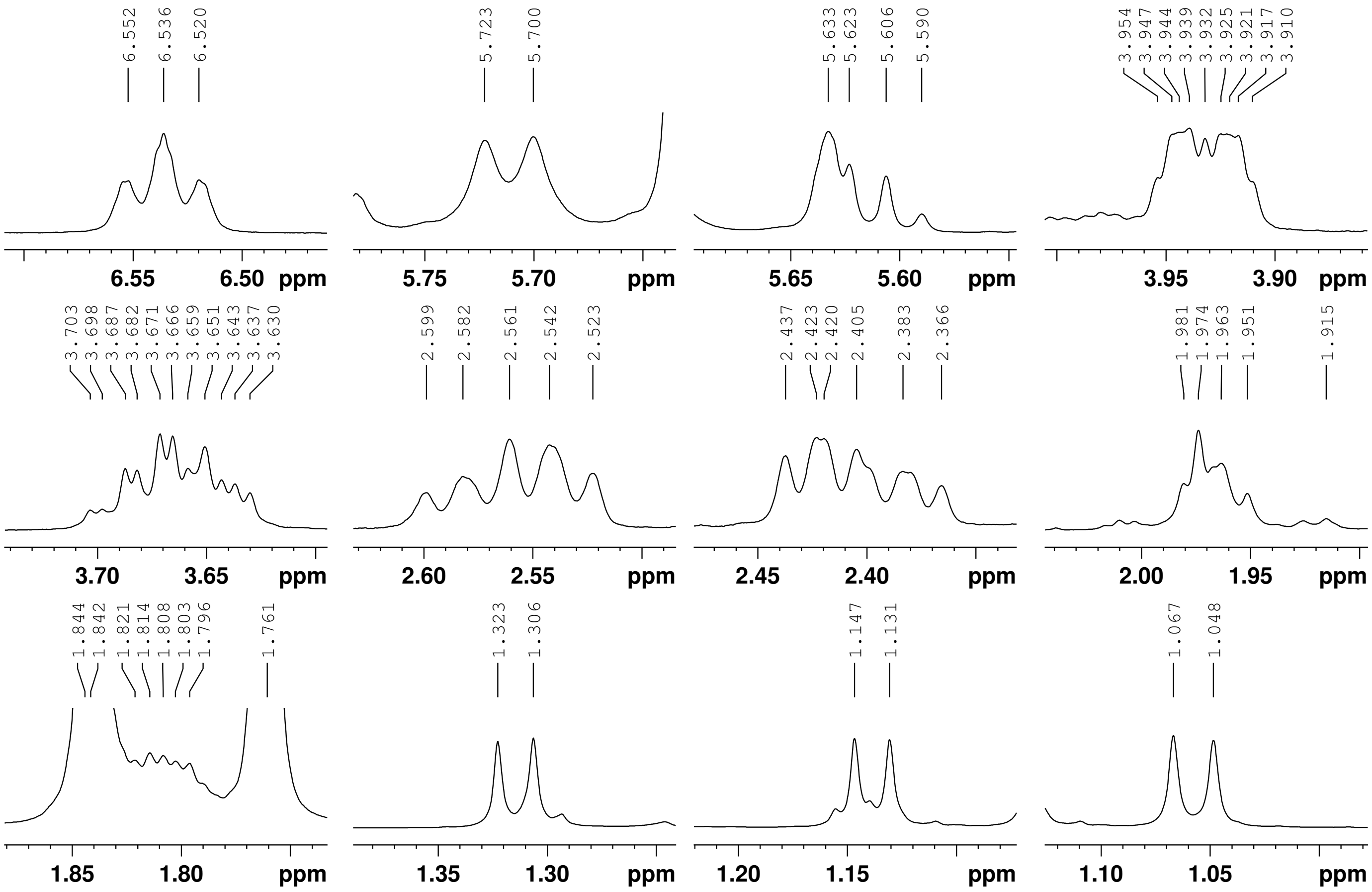

^{1}H NMR expansion of (*S*,*Z*)-*N*-((2*R*,3*R*,5*S*,6*S*)-2,5-dimethyl-6-((*E*)-3-methyl-4-oxobut-2-en-1-yl)tetrahydro-2*H*-pyran-3-yl)-4-(methoxymethoxy)-3-methylpent-2-enamide **17** (400 MHz, $CDCl_3$, 296K)

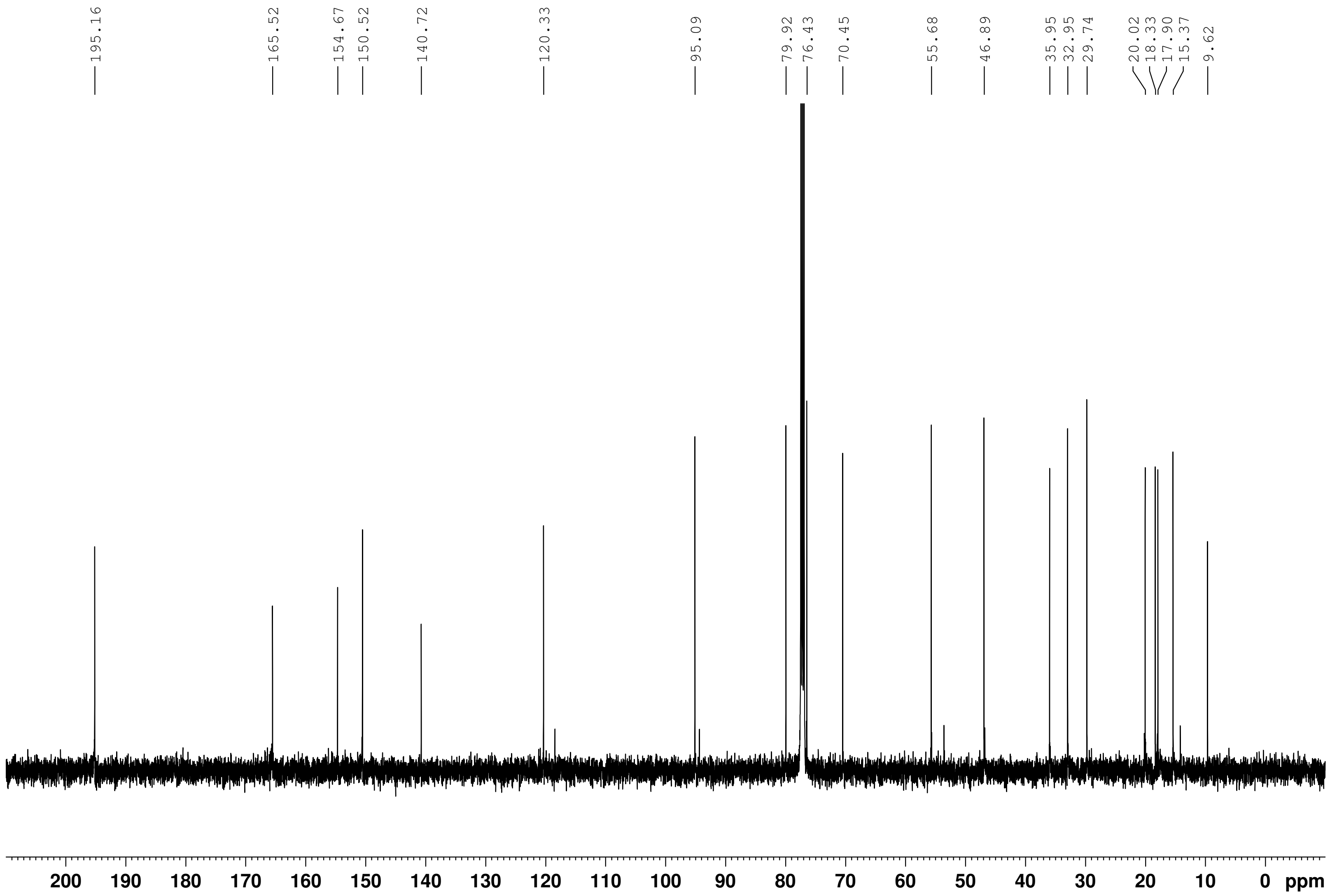

$^{13}C\{^{1}H\}$ NMR spectrum of (*S*,*Z*)-*N*-((2*R*,3*R*,5*S*,6*S*)-2,5-dimethyl-6-((*E*)-3-methyl-4-oxobut-2-en-1-yl)tetrahydro-2*H*-pyran-3-yl)-4-(methoxymethoxy)-3-methylpent-2-enamide **17** (125 MHz, $CDCl_3$, 296K)

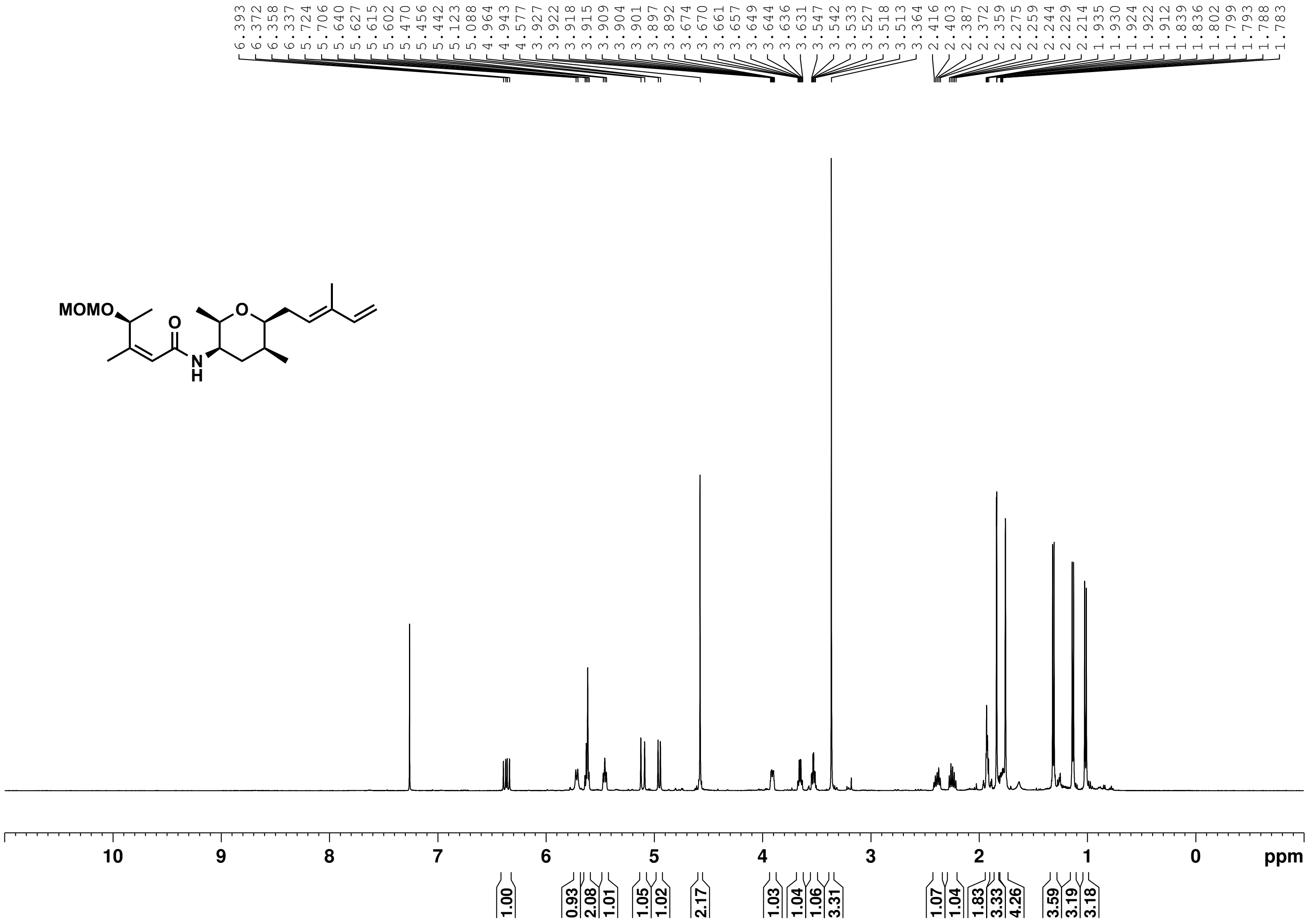

^{1}H NMR spectrum of (*S*,*Z*)-*N*-((2*R*,3*R*,5*S*,6*S*)-2,5-dimethyl-6-((*E*)-3-methylpenta-2,4-dien-1-yl)tetrahydro-2*H*-pyran-3-yl)-4-(methoxymethoxy)-3-methylpent-2-enamide **18** (500 MHz, $CDCl_3$, 296K)

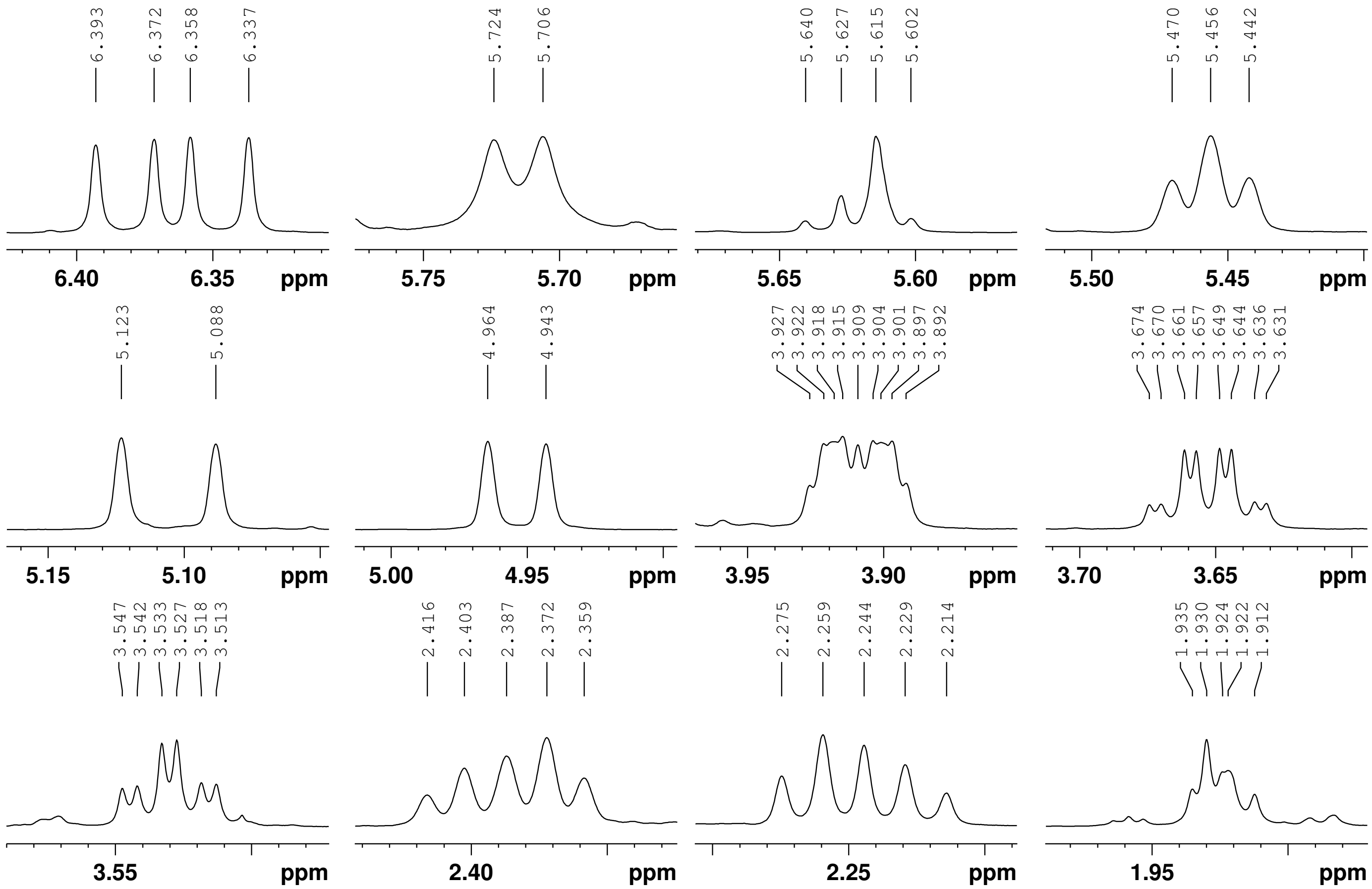

^{1}H NMR expansion of (*S*,*Z*)-*N*-((2*R*,3*R*,5*S*,6*S*)-2,5-dimethyl-6-((*E*)-3-methylpenta-2,4-dien-1-yl)tetrahydro-2*H*-pyran-3-yl)-4-(methoxymethoxy)-3-methylpent-2-enamide **18** (500 MHz, $CDCl_3$, 296K)

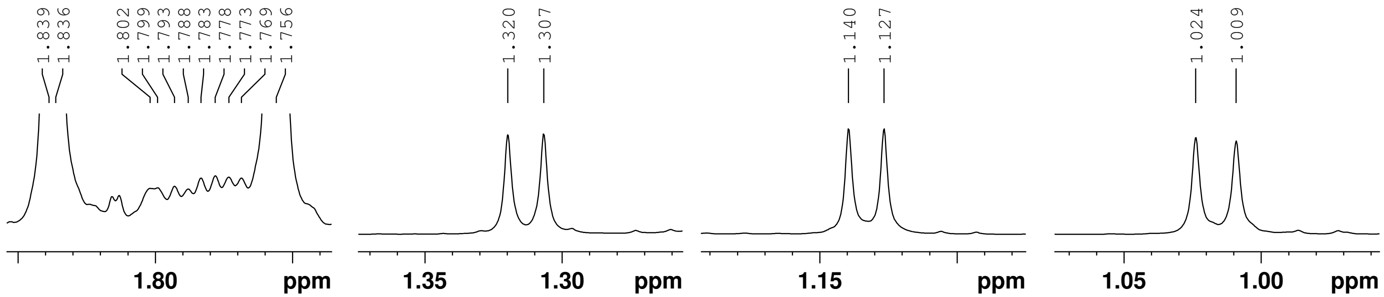

^{1}H NMR expansion of (*S*,*Z*)-*N*-((2*R*,3*R*,5*S*,6*S*)-2,5-dimethyl-6-((*E*)-3-methylpenta-2,4-dien-1-yl)tetrahydro-2*H*-pyran-3-yl)-4-(methoxymethoxy)-3-methylpent-2-enamide **18** (500 MHz, $CDCl_3$, 296K)

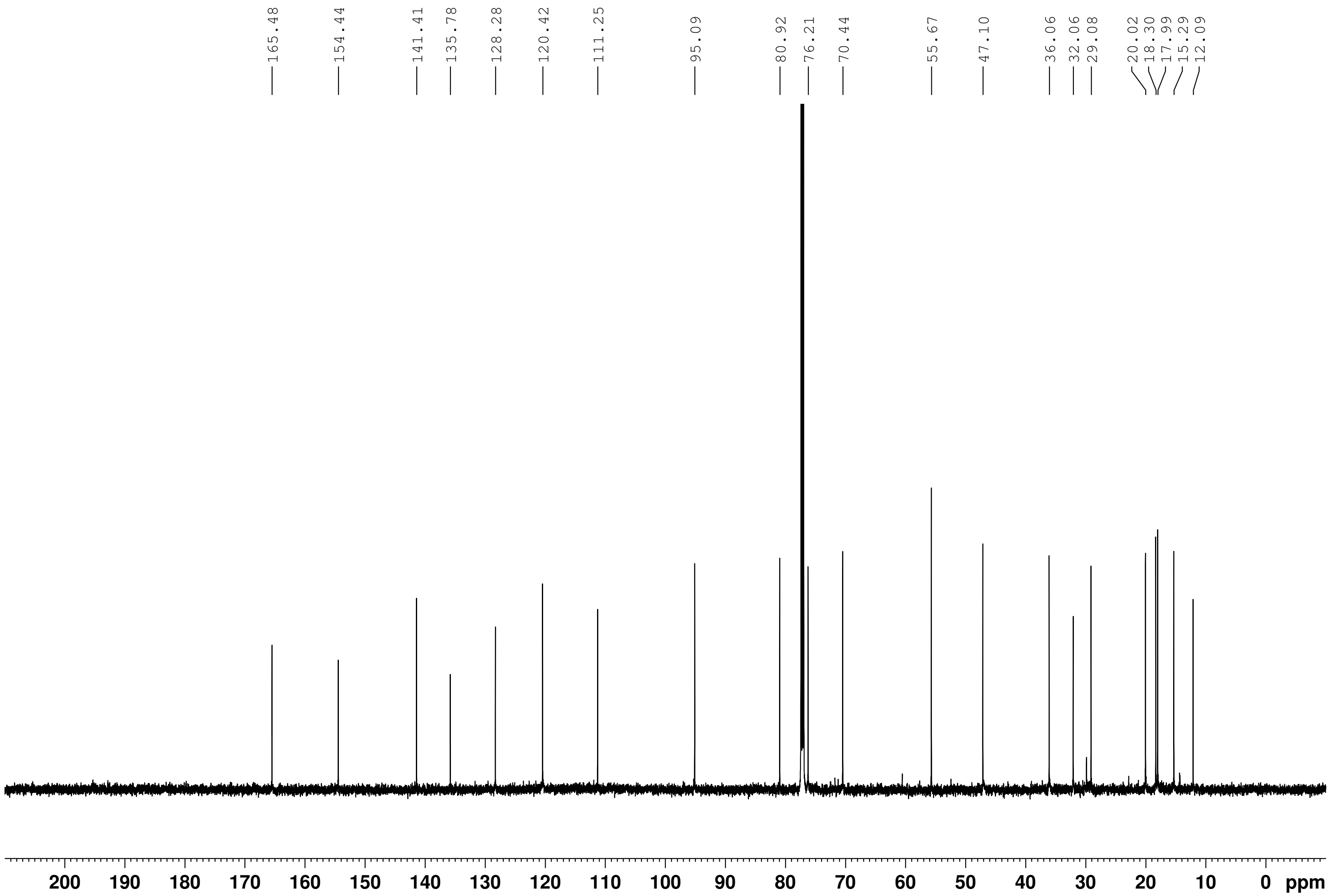

$^{13}C\{^1H\}$ NMR spectrum of (*S*,*Z*)-*N*-((2*R*,3*R*,5*S*,6*S*)-2,5-dimethyl-6-((*E*)-3-methylpenta-2,4-dien-1-yl)tetrahydro-2*H*-pyran-3-yl)-4-(methoxymethoxy)-3-methylpent-2-enamide **18** (150 MHz, $CDCl_3$, 296K)

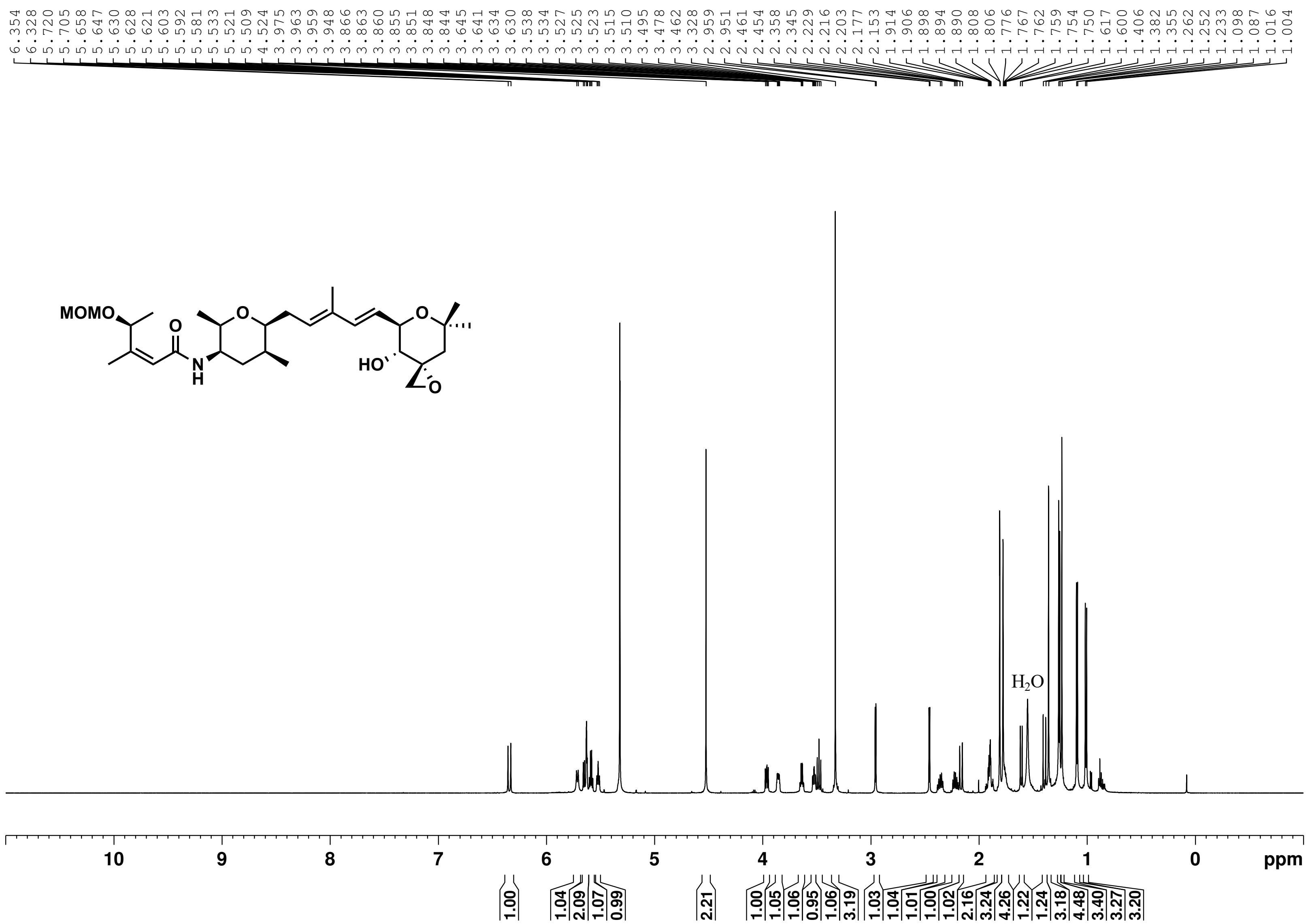

^{1}H NMR spectrum of 3'-Me meayamycin D (600 MHz, CD_2Cl_2, 296K)

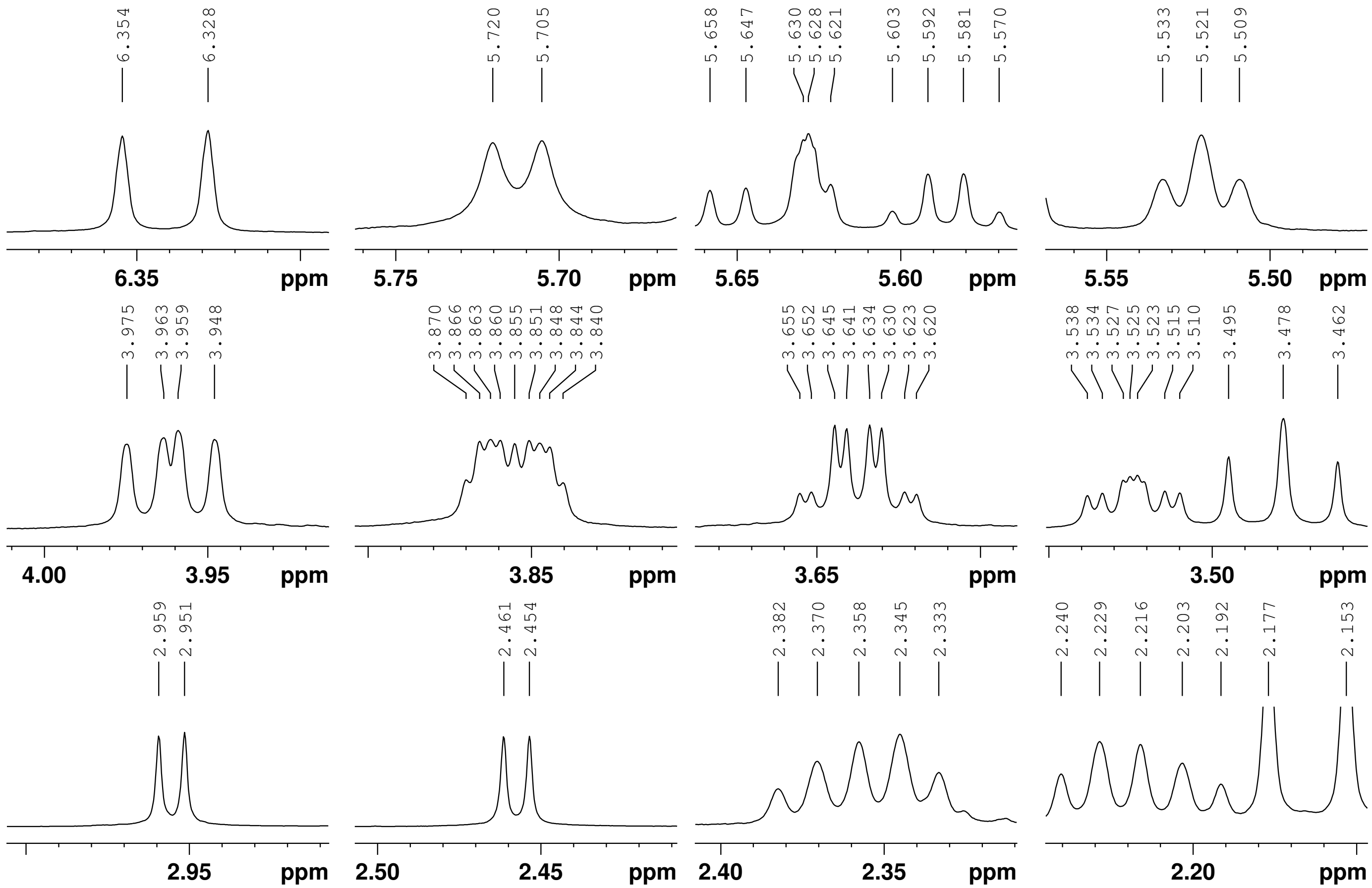

^{1}H NMR expansion of 3'-Me meayamycin D (600 MHz, CD_2Cl_2, 296K)

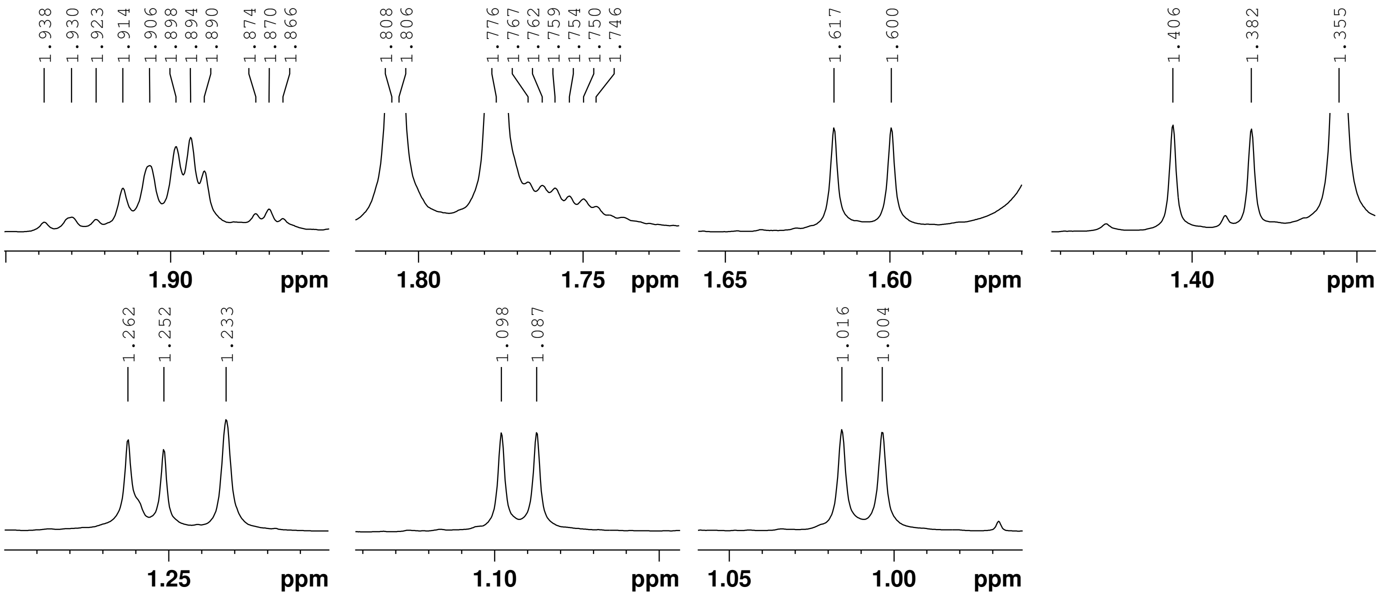

^{1}H NMR expansion of 3'-Me meayamycin D (600 MHz, CD_2Cl_2, 296K)

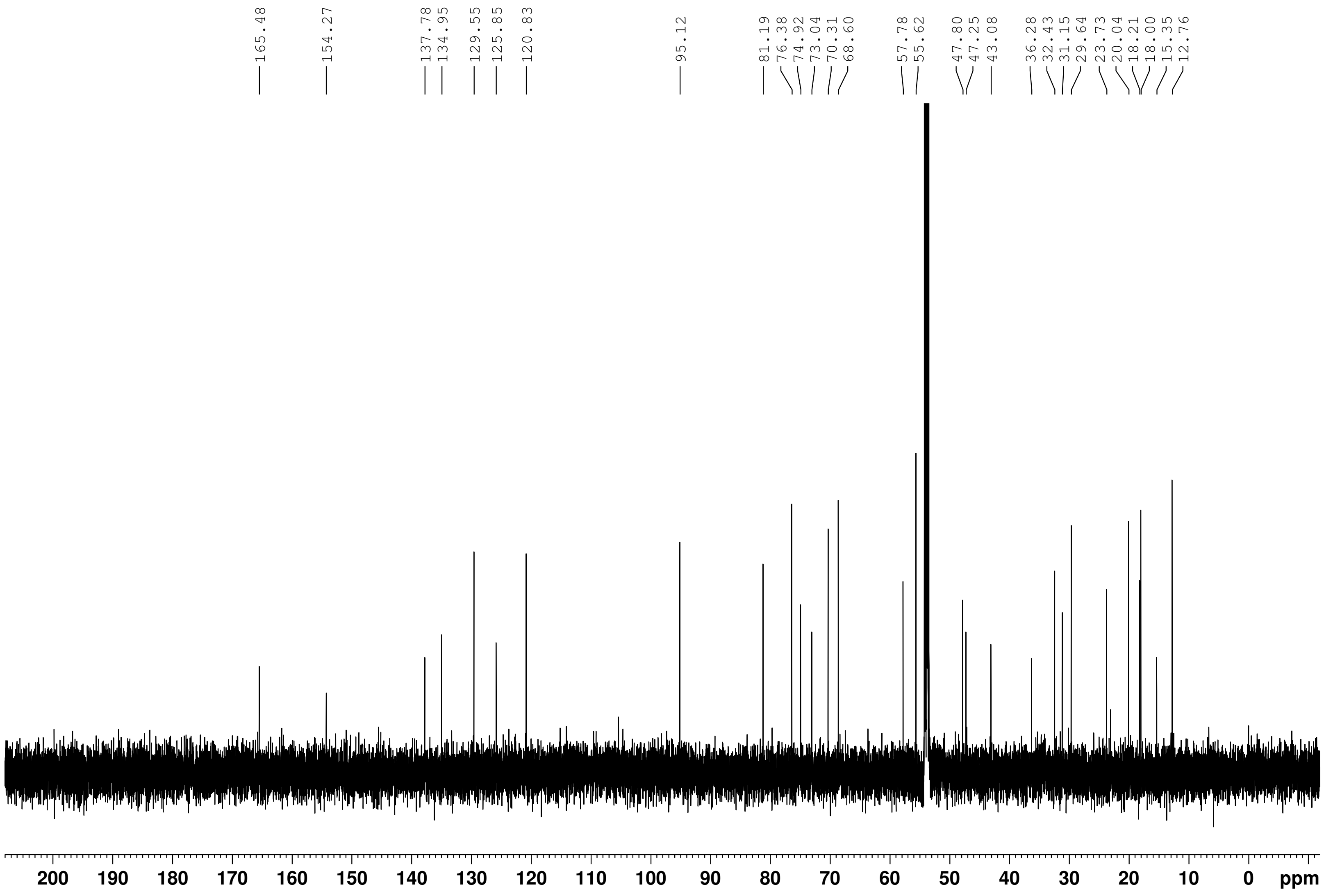

$^{13}C\{^1H\}$ NMR spectrum of 3'-Me meayamycin D (150 MHz, CD_2Cl_2, 296K)

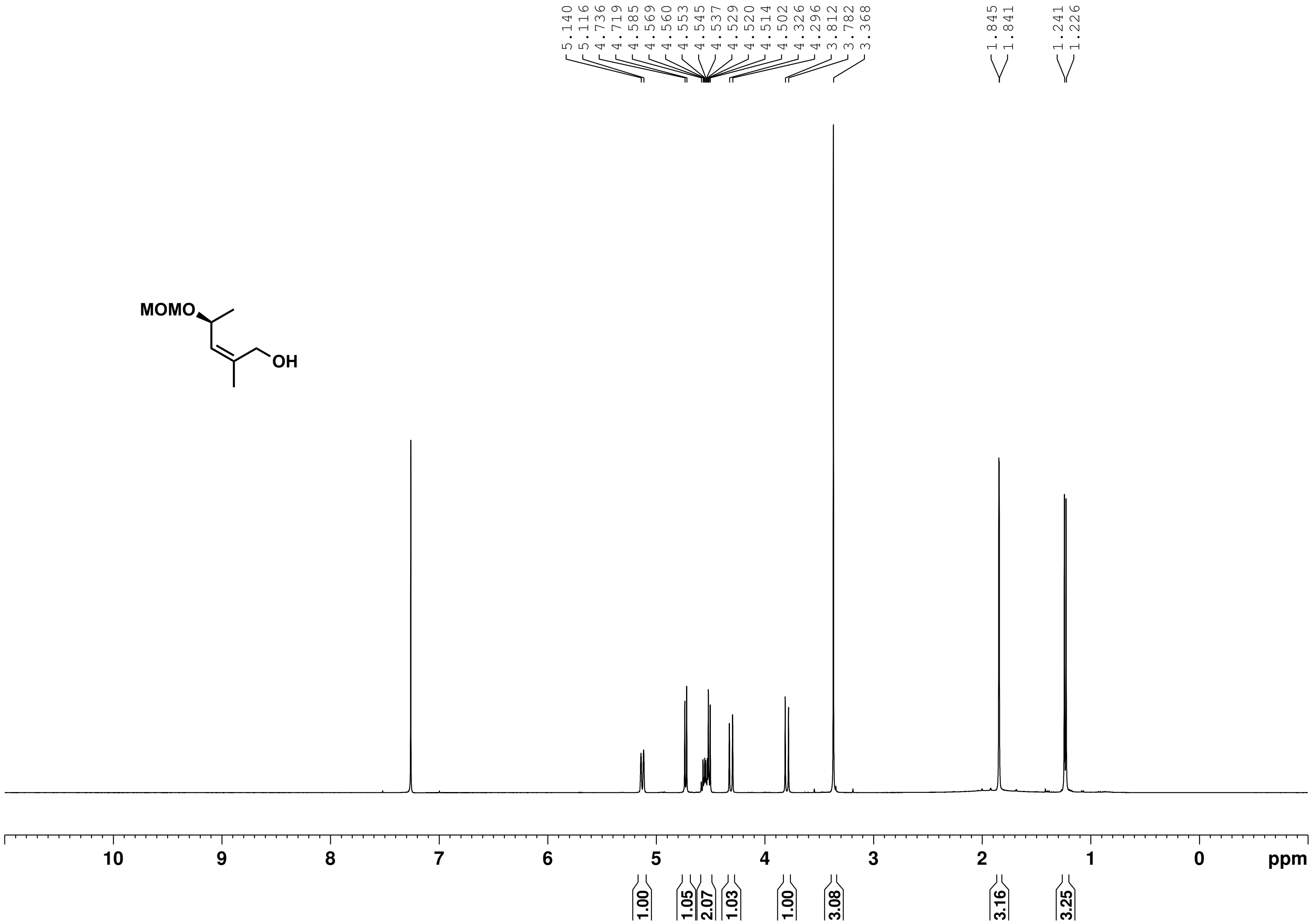

^{1}H NMR spectrum of (*S*,*Z*)-4-(methoxymethoxy)-2-methylpent-2-en-1-ol **8** (400 MHz, 1% CD_3OD in $CDCl_3$, 296K)

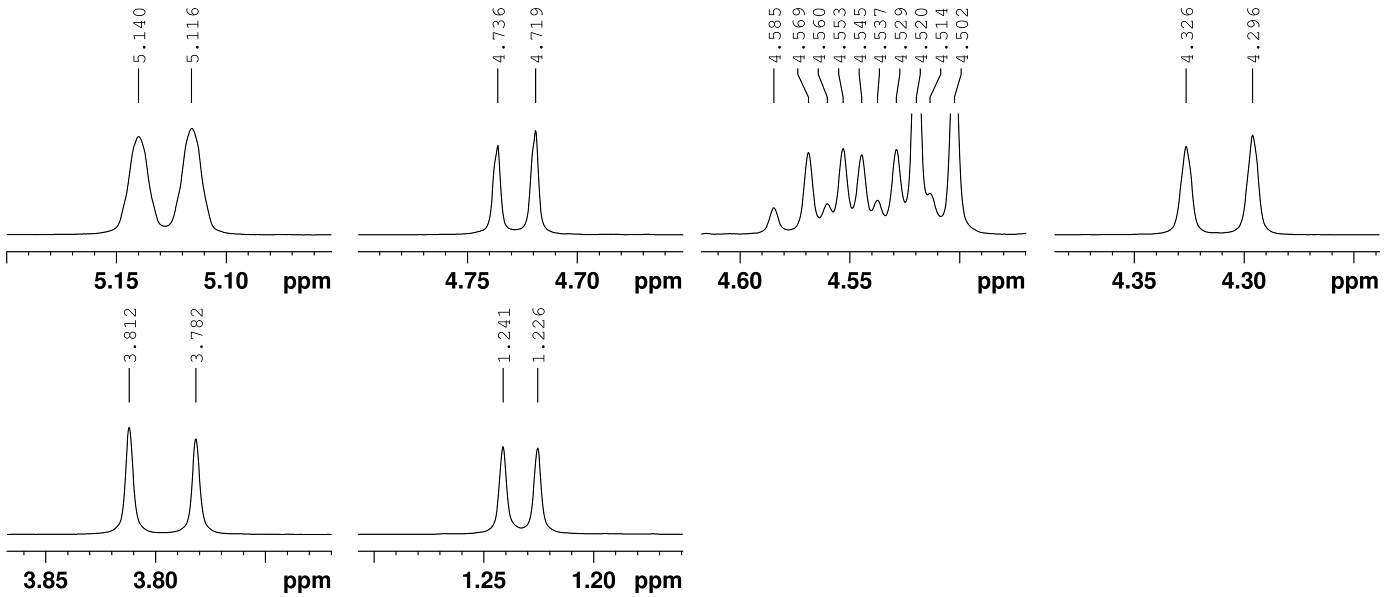

^{1}H NMR expansion of (*S*,*Z*)-4-(methoxymethoxy)-2-methylpent-2-en-1-ol **8** (400 MHz, 1% CD_3OD in $CDCl_3$, 296K)

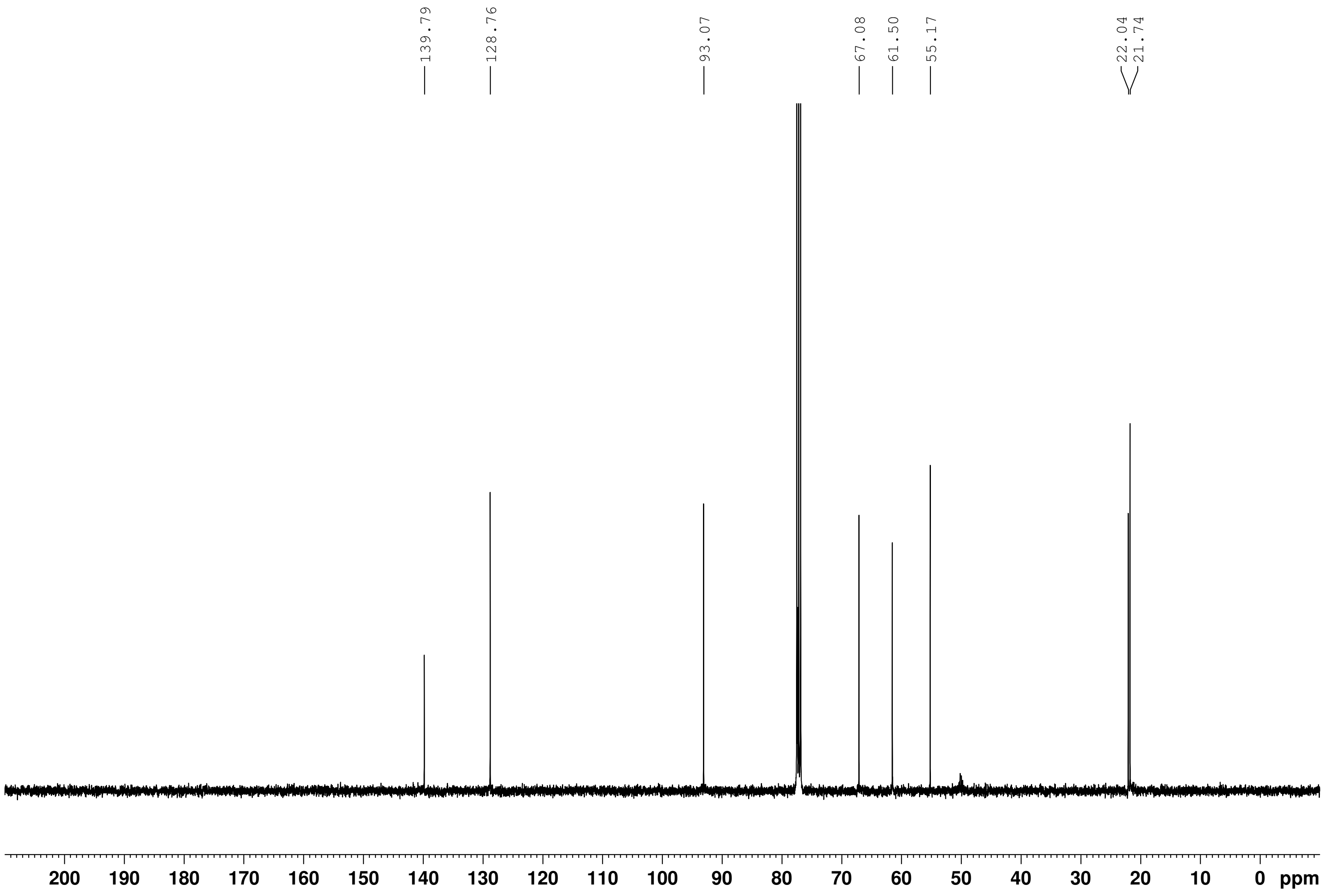

$^{13}C\{^{1}H\}$ NMR spectrum of (*S*,*Z*)-4-(methoxymethoxy)-2-methylpent-2-en-1-ol **8** (100 MHz, 1% CD_3OD in $CDCl_3$, 296K)

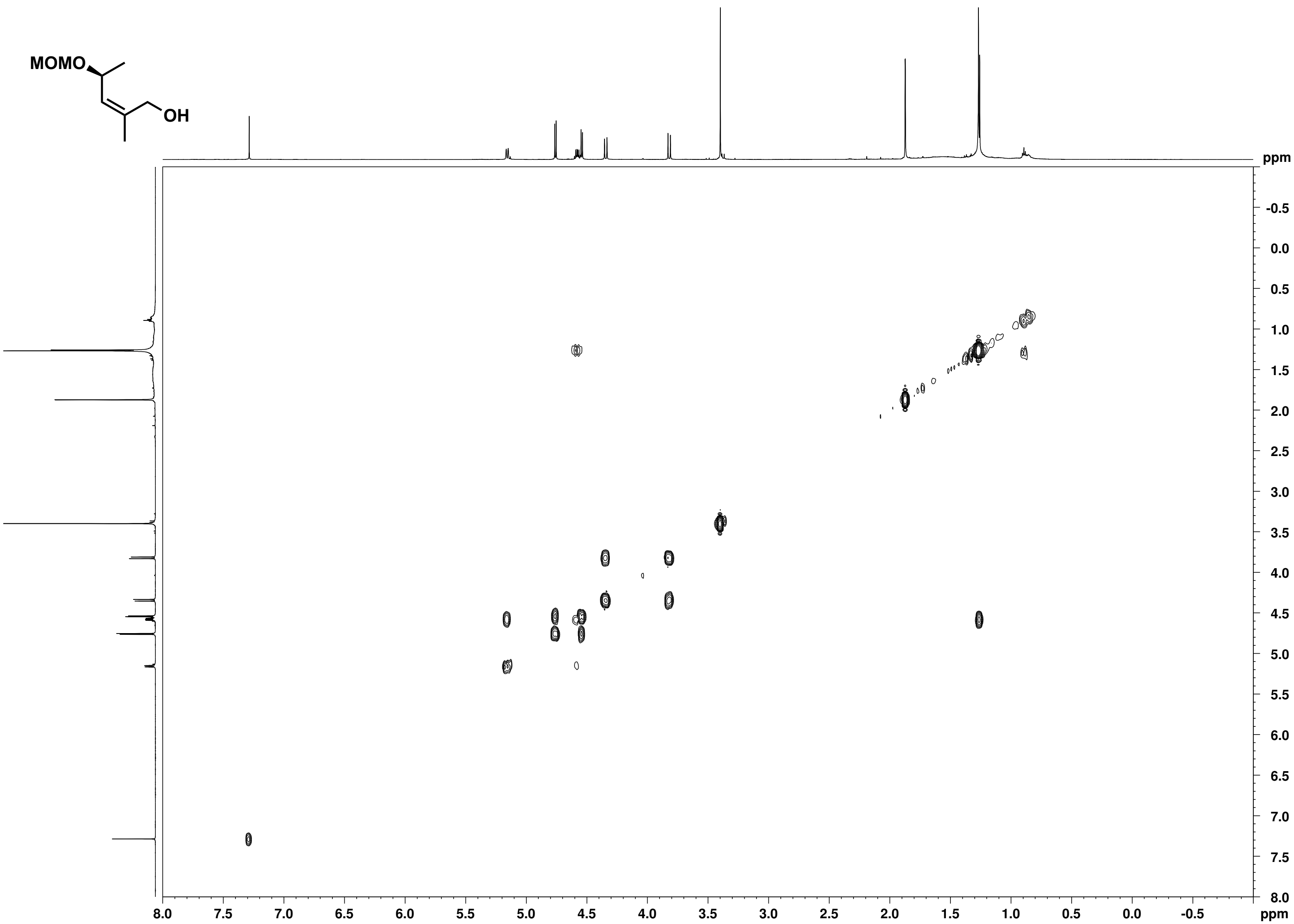

COSY NMR spectrum of (*S*,*Z*)-4-(methoxymethoxy)-2-methylpent-2-en-1-ol **8** (600 MHz, 1% CD_3OD in $CDCl_3$, 296K)

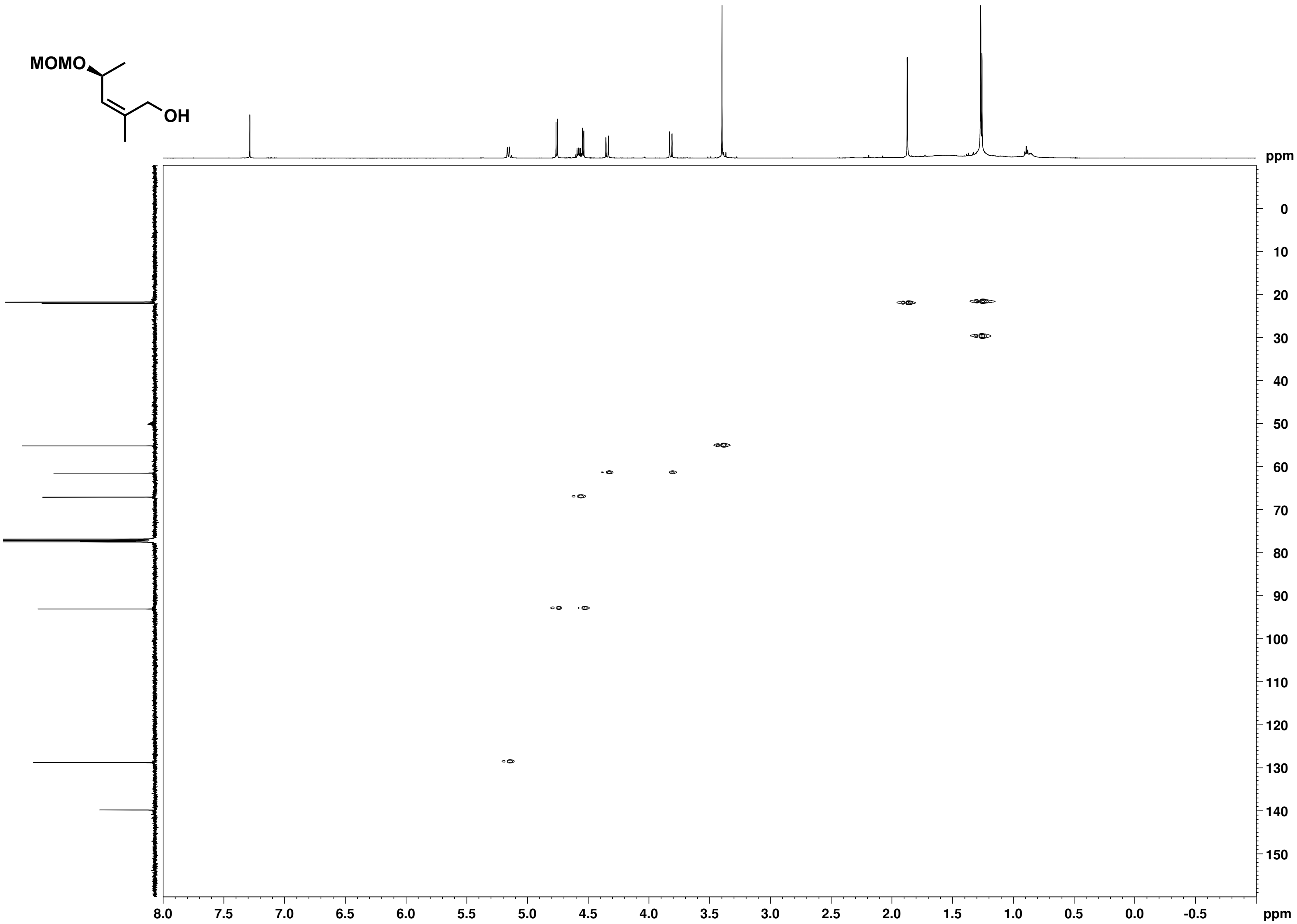

HSQC NMR spectrum of (*S*,*Z*)-4-(methoxymethoxy)-2-methylpent-2-en-1-ol **8** (600 MHz, 1% CD_3OD in $CDCl_3$, 296K)

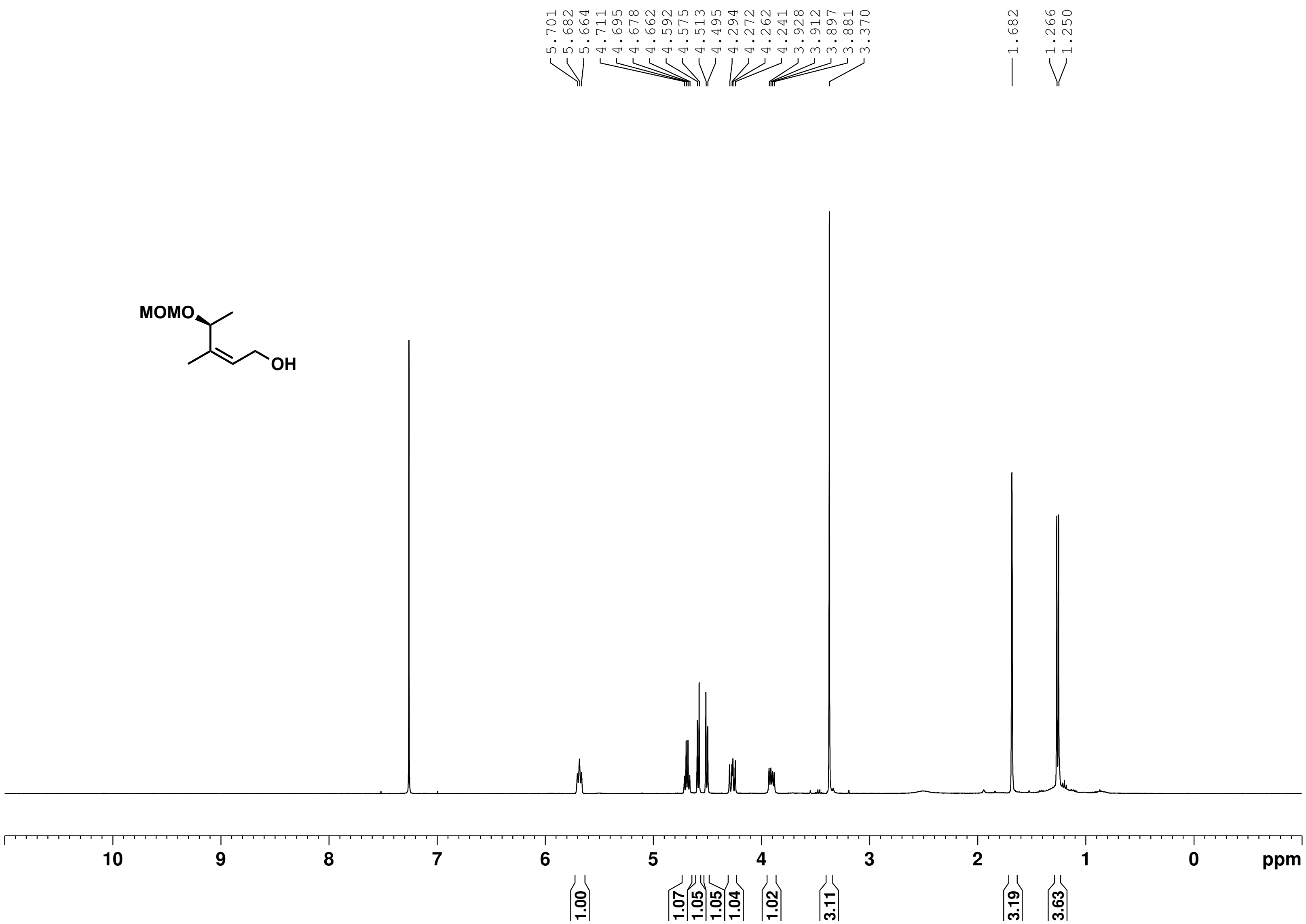

^{1}H NMR spectrum of (*S*,*Z*)-4-(methoxymethoxy)-3-methylpent-2-en-1-ol **19** (400 MHz, 1% CD_3OD in $CDCl_3$, 296K)

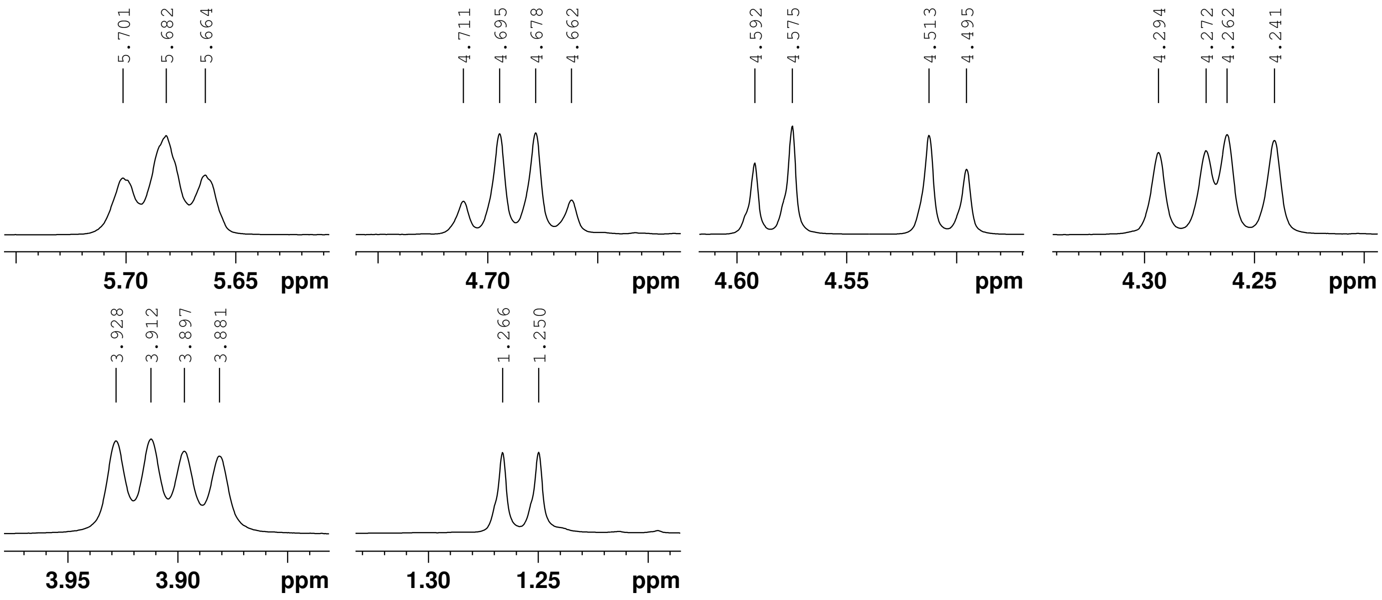

^{1}H NMR expansion of (*S*,*Z*)-4-(methoxymethoxy)-3-methylpent-2-en-1-ol **19** (400 MHz, 1% CD_3OD in $CDCl_3$, 296K)

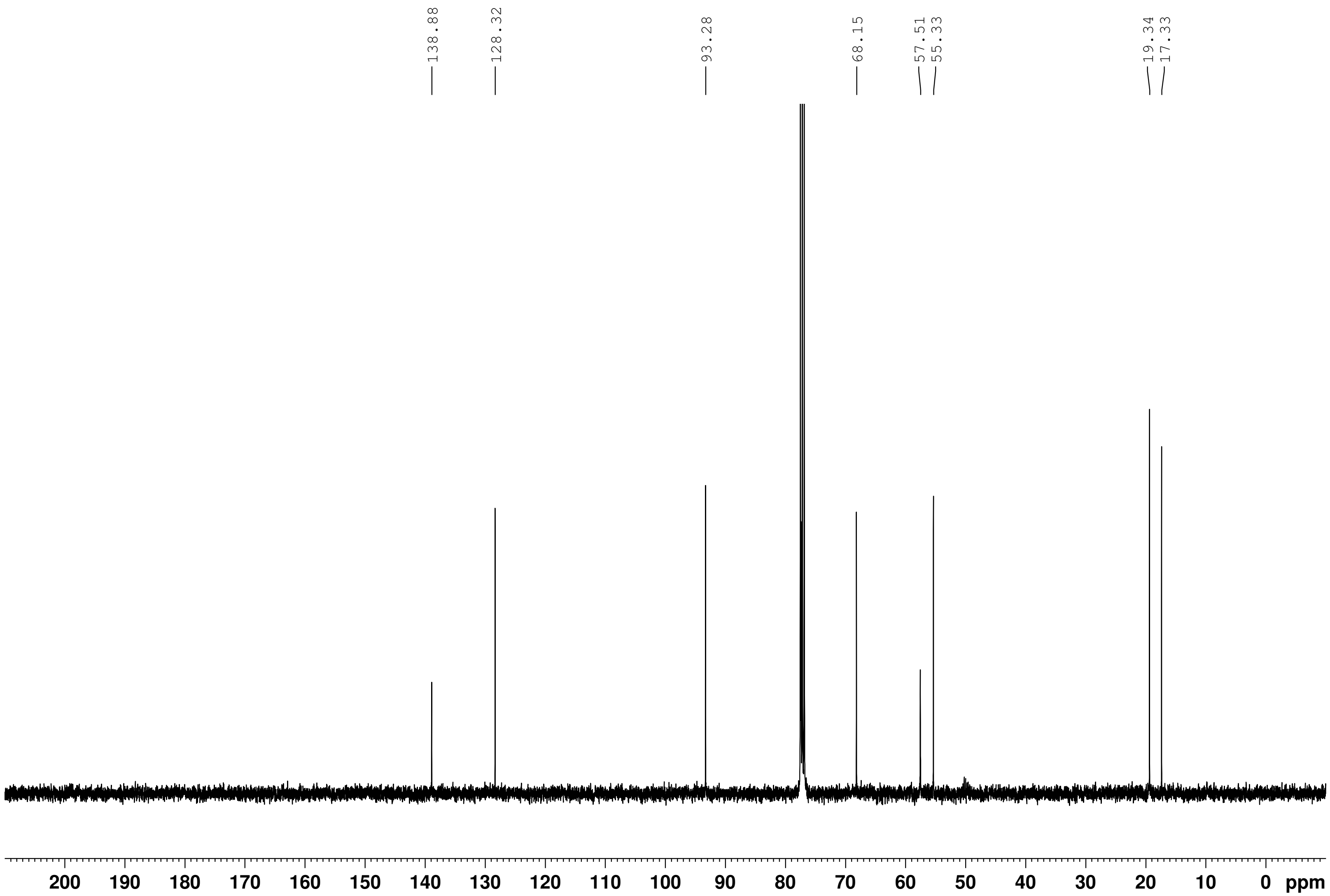

$^{13}C\{^{1}H\}$ NMR spectrum of (*S*,*Z*)-4-(methoxymethoxy)-3-methylpent-2-en-1-ol **19** (100 MHz, 1% CD_3OD in $CDCl_3$, 296K)

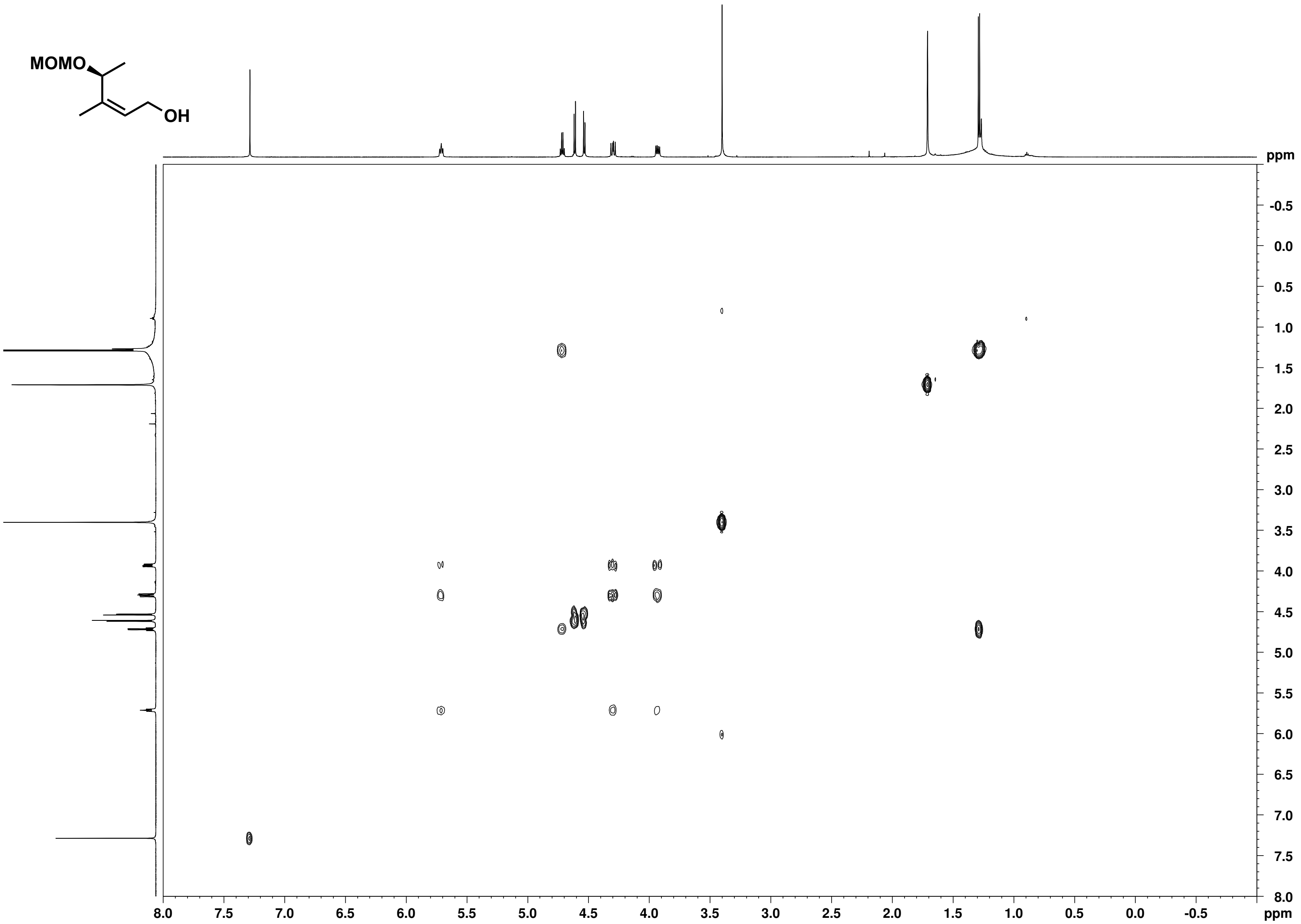

COSY NMR spectrum of (*S*,*Z*)-4-(methoxymethoxy)-3-methylpent-2-en-1-ol **19** (600 MHz, 1% CD_3OD in $CDCl_3$, 296K)

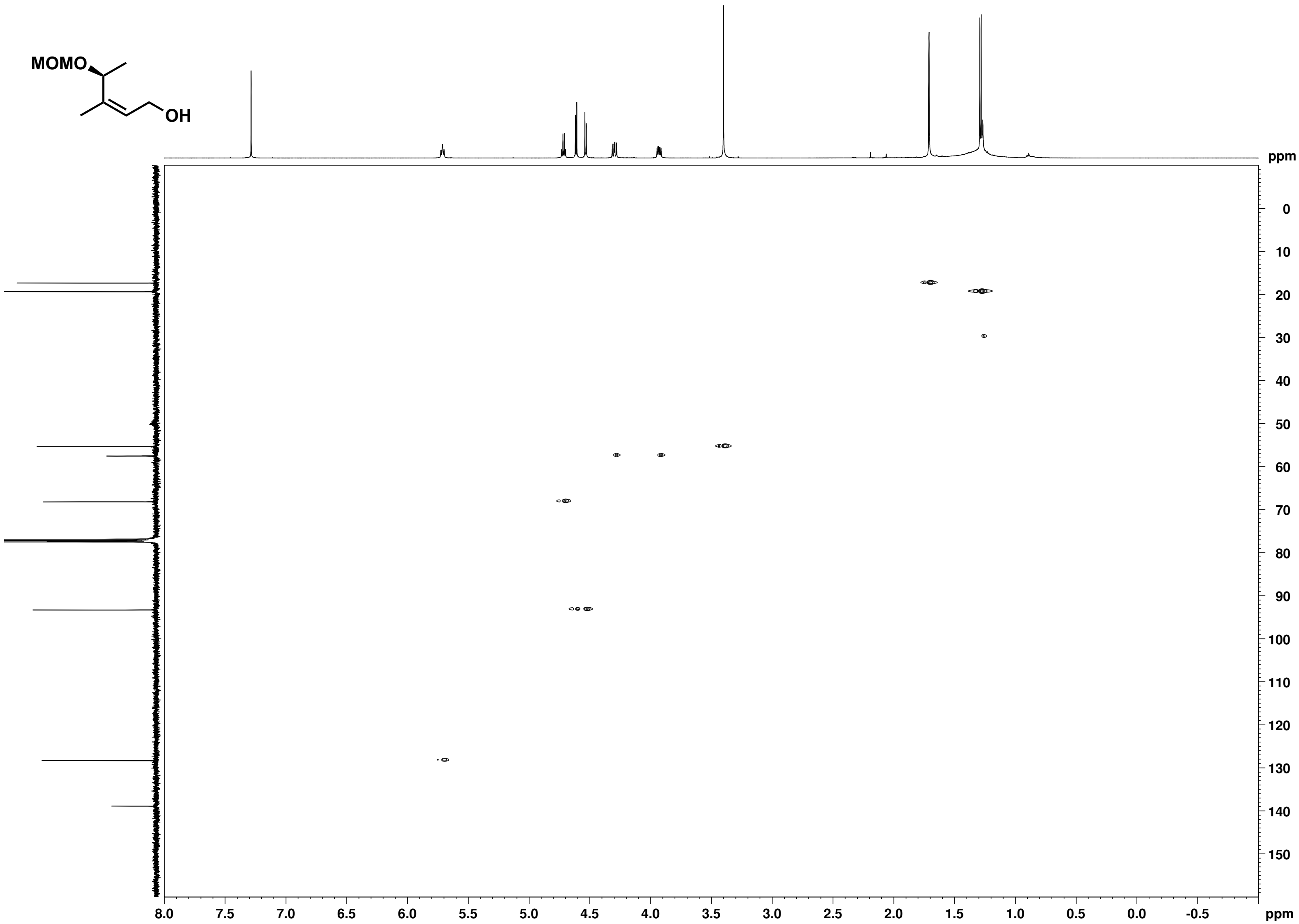

HSQC NMR spectrum of (*S*,*Z*)-4-(methoxymethoxy)-3-methylpent-2-en-1-ol **19** (600 MHz, 1% CD_3OD in $CDCl_3$, 296K)
